# Preclinical Lead Optimization of a 1,2,4-Triazole Based Tankyrase Inhibitor

**DOI:** 10.1021/acs.jmedchem.0c00208

**Published:** 2020-06-08

**Authors:** Jo Waaler, Ruben G. G. Leenders, Sven T. Sowa, Shoshy Alam Brinch, Max Lycke, Piotr Nieczypor, Sjoerd Aertssen, Sudarshan Murthy, Albert Galera-Prat, Eddy Damen, Anita Wegert, Marc Nazaré, Lari Lehtiö, Stefan Krauss

**Affiliations:** † Hybrid Technology Hub—Centre of Excellence, Institute of Basic Medical Sciences, University of Oslo, P.O. Box 1110 Blindern, 0317 Oslo, Norway; ‡ Department of Immunology and Transfusion Medicine, Oslo University Hospital, P.O. Box 4950 Nydalen, 0424 Oslo, Norway; § Mercachem BV, Kerkenbos 1013, 6546 BB Nijmegen, The Netherlands; ∥ Faculty of Biochemistry and Molecular Medicine, Biocenter Oulu, University of Oulu, P.O. Box 5400, 90014 Oulu, Finland; ⊥ Medicinal Chemistry, Leibniz-Forschungsinstitut für Molekulare Pharmakologie (FMP), Campus Berlin Buch, Robert-Roessle-Straße 10, 13125 Berlin, Germany

## Abstract

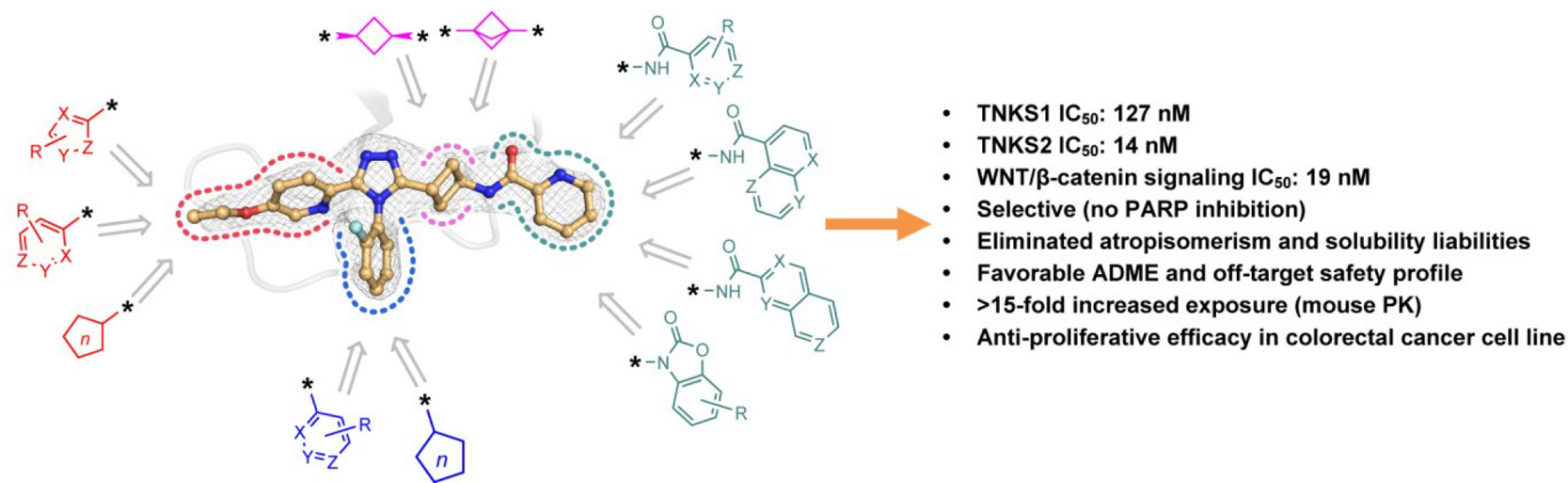

Tankyrases 1 and 2 are central biotargets in the WNT/β-catenin signaling and Hippo signaling pathways. We have previously developed tankyrase inhibitors bearing a 1,2,4-triazole moiety and binding predominantly to the adenosine binding site of the tankyrase catalytic domain. Here we describe a systematic structure-guided lead optimization approach of these tankyrase inhibitors. The central 1,2,4-triazole template and *trans-*cyclobutyl linker of the lead compound **1** were left unchanged, while side-group East, West, and South moieties were altered by introducing different building blocks defined as point mutations. The systematic study provided a novel series of compounds reaching picomolar IC_50_ inhibition in WNT/β*-*catenin signaling cellular reporter assay. The novel optimized lead **13** resolves previous atropisomerism, solubility, and Caco-2 efflux liabilities. **13** shows a favorable ADME profile, including improved Caco-2 permeability and oral bioavailability in mice, and exhibits antiproliferative efficacy in the colon cancer cell line COLO 320DM *in vitro.*

## Introduction

Tankyrase 1 and tankyrase 2 (TNKS1/2) are members of the PARP family of enzymes that control protein activities, interactions, and turnover through mono- or poly-ADP-ribosylation.^1^ TNKS1/2 regulate a number of target proteins, including AXIN1 and AXIN2 (AXIN1/2) in the β-catenin destruction complex resulting in WNT/β*-*catenin signaling pathway inhibition, and AMOT proteins in the Hippo signaling pathway resulting in YAP signaling inhibition.^1−3^ Tankyrases, through their ankyrin repeat clusters, bind to AXIN1/2, making AXIN1/2 accessible for ADP-ribosylation by the C-terminal TNKS1/2 catalytic domain.^1^ AXIN1/2 is subsequently targeted for proteasomal degradation through polyubiquitination of E3 ubiquitin ligase RNF146, recognizing the poly-ADP-ribose signal.^1,2^ Destabilization of AXIN1/2, being a structural protein in the β*-*catenin destruction complex, leads to increased β*-*catenin levels which can be counteracted by inhibition of TNKS1/2 catalytic activity.^1,2^ Similarly, TNKS1/2 control the stability of AMOT proteins via RNF146. Stabilization of AMOT proteins by inhibiting TNKS1/2 activity sequesters YAP to the cytoplasm and prevents target gene expression driven by YAP in the nucleus.^1,3^ TNKS1/2 catalytic activities also interfere with other biological mechanisms and cell signaling pathways such as vesicle transport, energy metabolism, telomere homeostasis, and mitotic spindle formation and affect components in AKT/PI3K and AMPK signaling pathways.^1,4−6^

Several groups of chemical substances have been identified that inhibit TNKS1/2 by binding to the substrate NAD^+^ binding site either by occupying a nicotinamide pocket, adenosine binding pocket or by addressing both of them.^2,7−20^ Although the catalytic domains of 17 human ARTD/PARP enzymes are homologous, unique features in the TNKS1/2 catalytic domain allow the development of tankyrase-selective chemical inhibitors.^1^ Despite this progress, there is currently no viable selective TNKS1/2 inhibitor in clinical testing or practice for any application including targeting the WNT/β*-*catenin and YAP signaling pathways in cancer therapy.^17,21−23^

It has been shown that TNKS1/2 inhibitors can exhibit anticancer efficacy in mouse models, either as monotherapy against colorectal cancer^8,24^ and osteosarcoma^25^ or in combination therapies together with PI3K and EGFR inhibitors against colorectal cancer^26^ or with PD-1 inhibition against melanoma.^27^

Two reports indicate intestinal toxicity^24^ and bone loss^28^ in mouse models upon treatment with early lead-stage tankyrase inhibitors, while other reports do not document signs of toxicity, intestinal injury, or body weight changes.^8,26,27,29^ Hence, there is a continued need for the development of safe drugs directed toward TNKS1/2 and the WNT/β*-*catenin signaling pathway with improved chemical and biophysical properties.^17,21−23^

The compound optimization described here is based on the understanding of the structure–activity relationship, crystallography, and physicochemical properties of our previous 1,2,4-triazole analogue series JW74,^30^ G007-LK,^9^ and OD336 (**1**).^11^ The optimization focused especially on the solubility and atropisomerism liabilities of the former G007-LK and **1** compounds, respectively. In our work we developed a novel series of compounds reaching picomolar IC_50_ activity in a cellular WNT/β*-*catenin signaling reporter assay. Lead compound OM-1700 (**13**) within the novel series displays high potency and specificity and has overall favorable ADME properties compared to benchmark tankyrase inhibitors.

## Results and Discussion

### Chemistry

**Figure 1:**
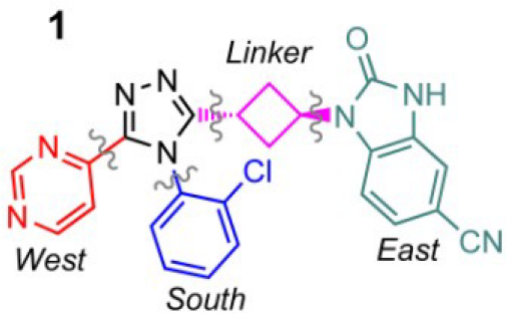
Lead compound **1** and the building blocks defined as West (red), South (blue), East (green), and linker (pink).

**Scheme 1:**
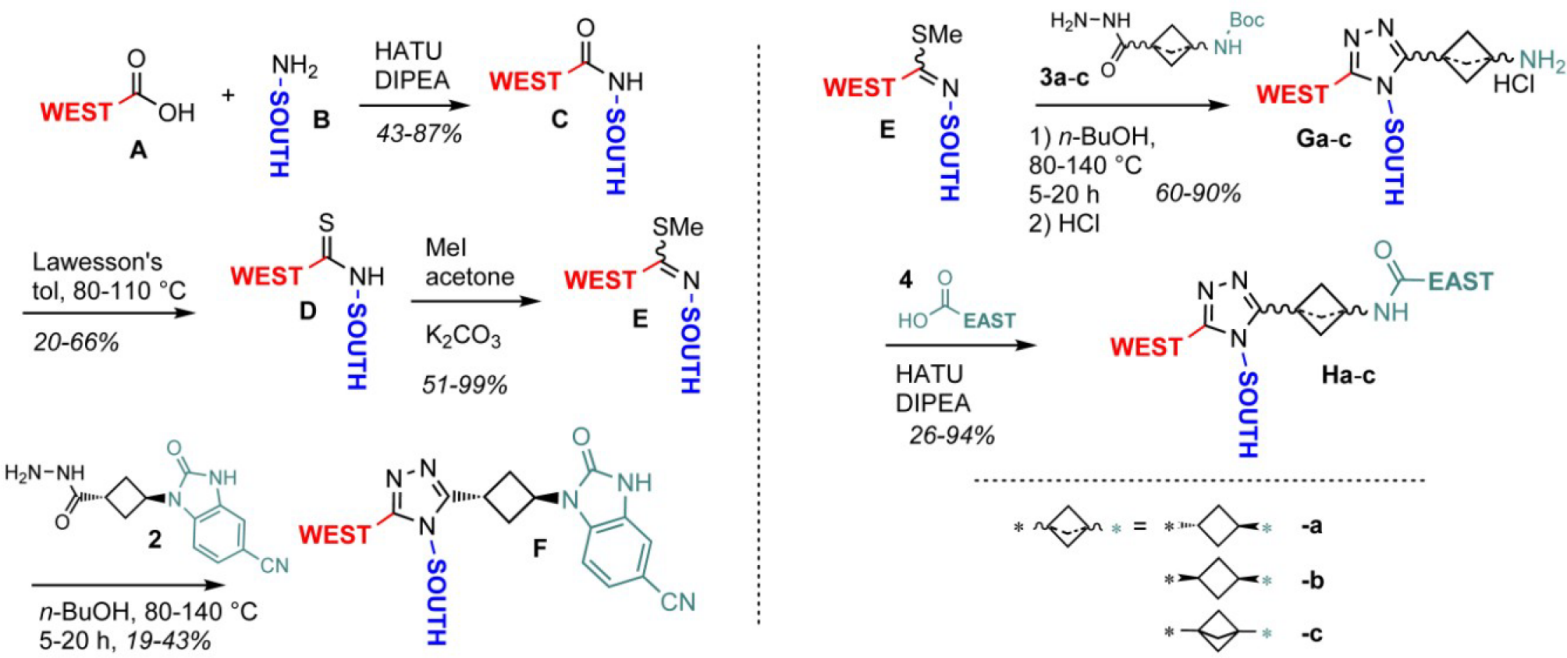
(a, Left) Synthesis of Targets with South and West Variations and (b, Right) Synthesis of Targets with East Variations

**Scheme 2:**
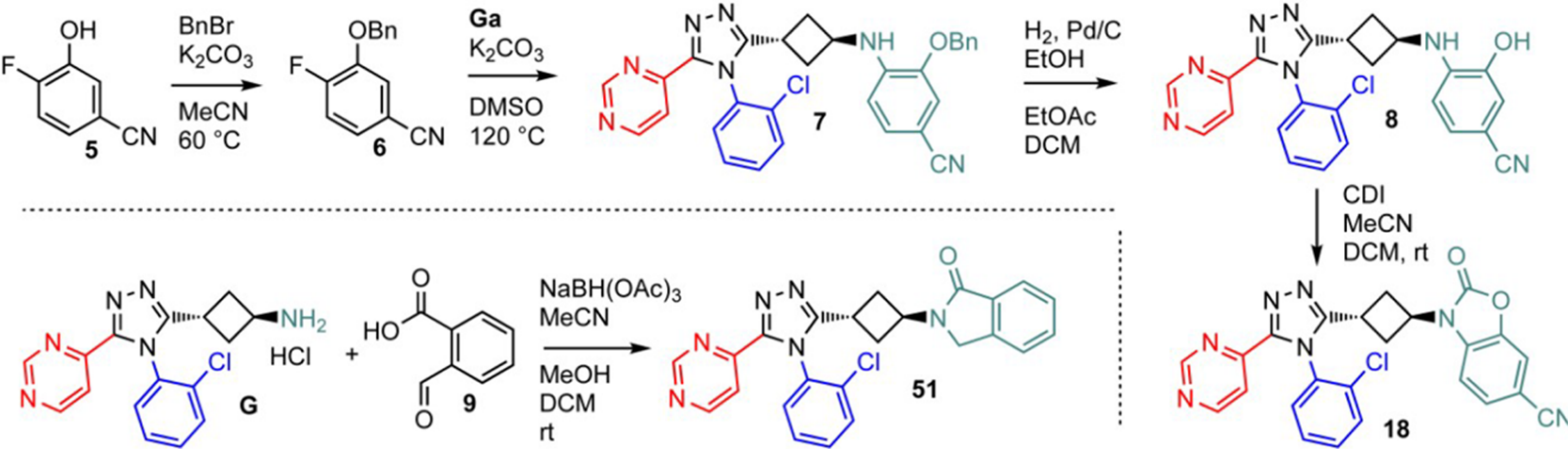
Synthesis of **18** and **51**

For the synthesis of novel structures in the optimization campaign, we embraced a building block approach (Figure 1). Herein we were able to prepare all compounds following the same synthetic route, simplifying synthesis efforts (Scheme 1). Cyclic amide/urea/carbamate East modifications, however, required a different route (Scheme 2). Since the 1,2,4-triazole as a central scaffold was well established in our previous research,^9^ it was left unchanged in the present lead optimization process. In the linker area between the 1,2,4-triazole and the East moieties, we synthesized a series of analogues with a bicyclo[1.1.1]pentane configuration and one compound with a *cis*-cyclobutane setup. These linker variations were synthesized according to the same scheme as for the default *trans* linker. For further optimization, the *trans-*cyclobutyl linker was left unchanged for the majority of the target molecules as it proved to be superior to the tested alternative linker iterations.^11^

For compounds having the benzimidazolone West moiety of **1**, synthesis was performed as depicted in Scheme 1a and described in our previous work.^11^ For East-side variations, a slightly different route enabling East variations in the last step departing from amine **G** was used (Scheme 1b).

As a first iteration, we replaced the East benzimidazolone group as in our experience this group can result in unwanted solubility, permeability, and efflux properties. When suitable East-amides were identified as a replacement for the benzimidazolone group, a wide range of further options for East-side iterations opened enabling fine-tuning of physicochemical properties. Next, we replaced the West-pyrimidine as this group renders the molecule vulnerable for CYP-mediated oxidation which was confirmed by Med-Id studies of **1**.

To synthesize a broad set of targets, we optimized the triazole-forming reaction from **E** to **G** (Scheme 1b) from the existing method (TFA, DMA, 120 °C, 14 h, 10–21% yield). Here, we found that heating of **E** and **3a** in 1-butanol at 80–140 °C for 5–20 h, depending on the actual substrate, typically resulted in 60–90% yield. Under these conditions, a broader scope of South and West moieties was tolerated in the reaction. All compounds in the present study were prepared accordingly except the benzisoxazolone **18** and lactam **51** (Scheme 2). For compounds with the *cis*-cyclobutane (**75**) and bicyclo[1.1.1]pentane (**19**) moieties, the corresponding hydrazides **3b** and **3c** respectively were used (Scheme 1b).

### Biological Evaluation

**Figure 2:**
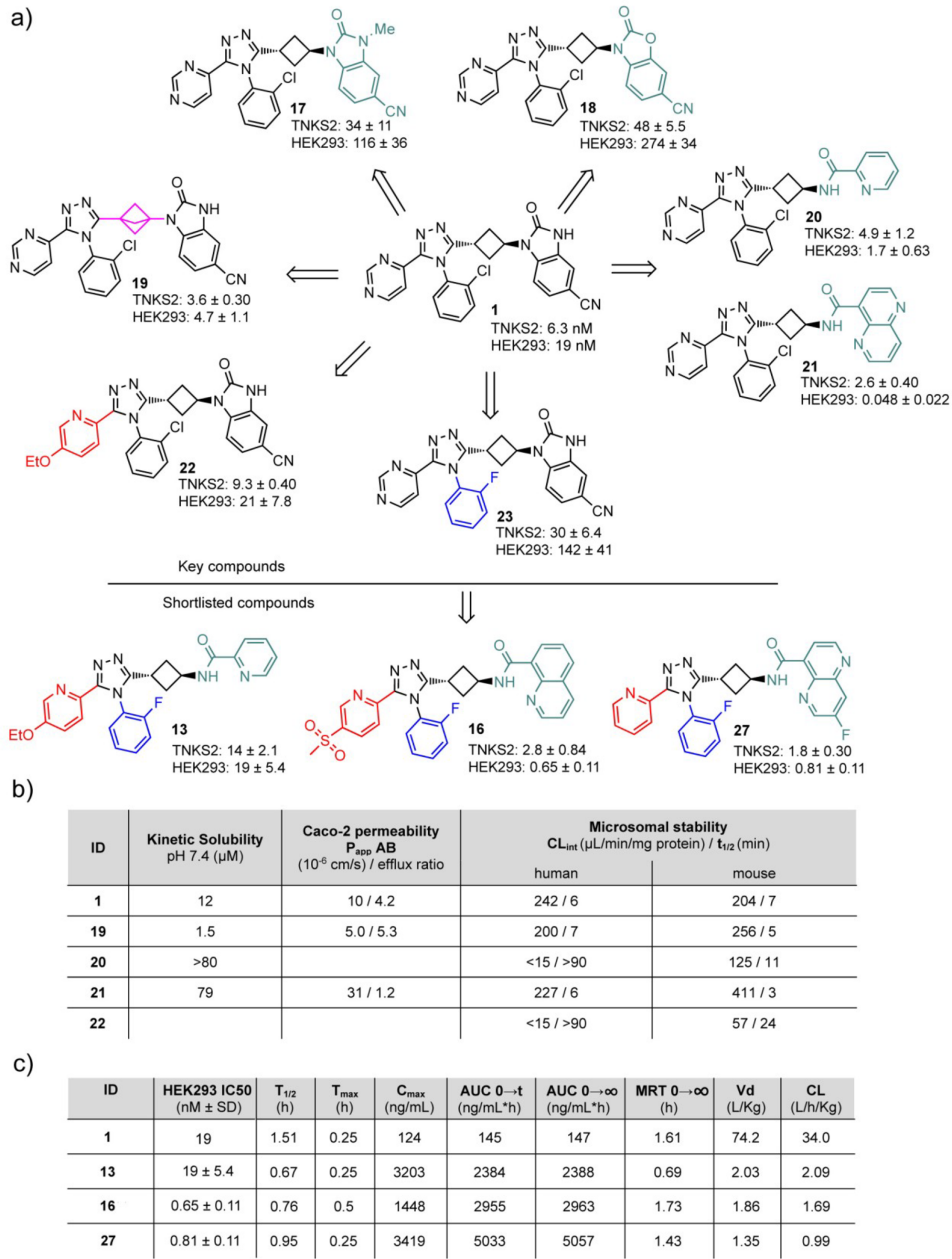
(a) Selected key compounds in the optimization campaign and respective TNKS2 and HEK293 IC_50_ values. Mean ± SD values for three independent experiments are shown. Moieties are in color when differing from **1**. (b) ADME data of key compounds. (c) Cellular efficacy and mouse po PK 5 mg/kg of the short-listed compounds.

All compounds were tested using a TNKS2 biochemical assay and a luciferase-based WNT/β*-*catenin signaling pathway reporter assay in human HEK293 cells.^9^ In the first round of the optimization campaign, we prepared single-point modifications changing any of the four regions in **1** (Figure 1) while leaving the other regions constant. In the following stages of the optimization campaign, additional East, West, and South moieties were also utilized, combining the best structural elements of the first single-point modification round. For the optimization, *in vitro* ADME properties and solubility of selected compounds were measured. Mouse pharmacokinetics, after oral dosing, was tested for the selected and short-listed compounds **13**, **16**, and **27** (Figure 2c and indicated by # in Tables 1 and 2).

**Table 1:**
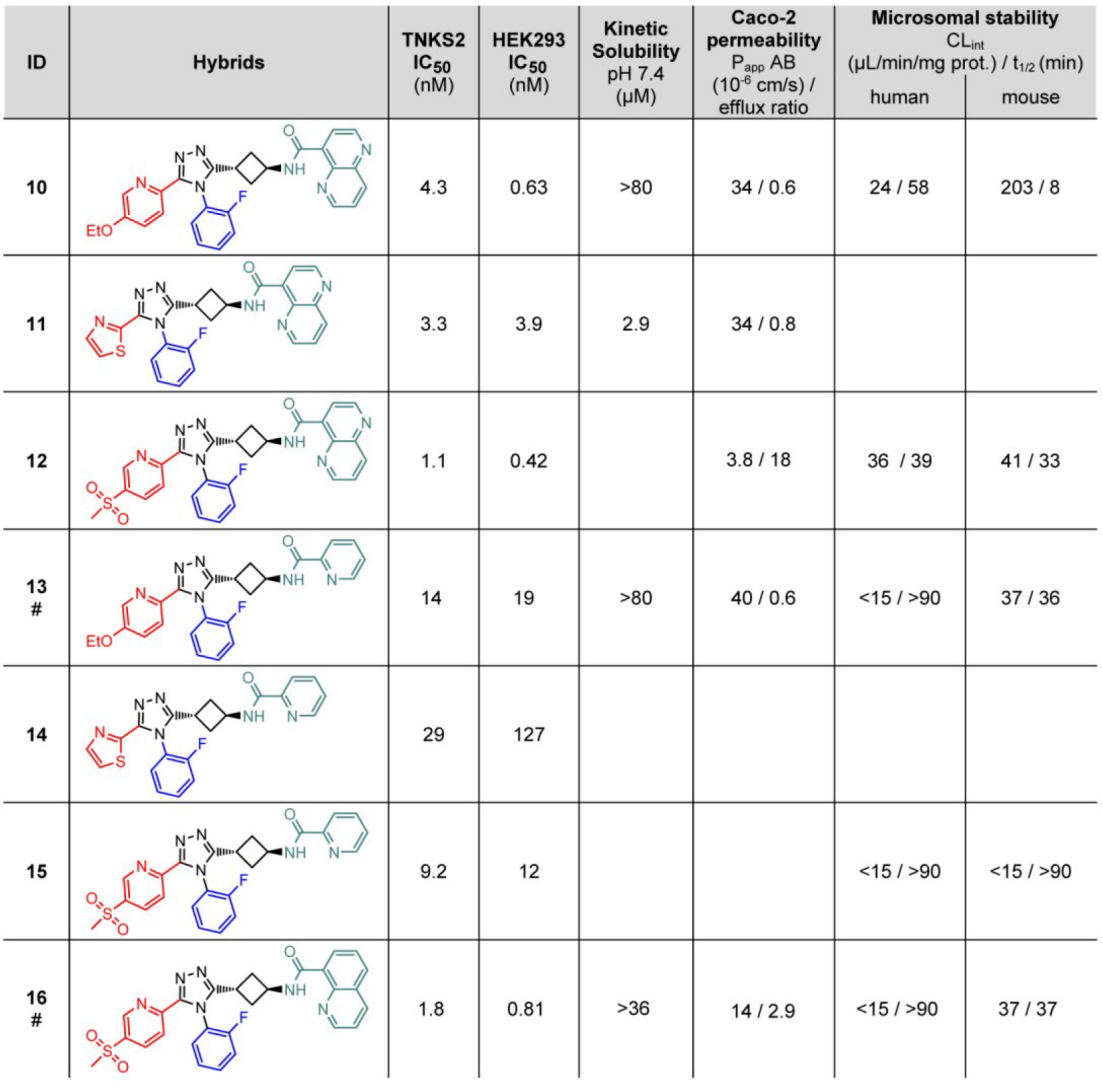
Hybrids Derived from **19** and **20**, **22**, and three West Moieties^a^

a # indicates that these compounds were evaluated in a mouse pharmacokinetics analysis.

**Table 2:**
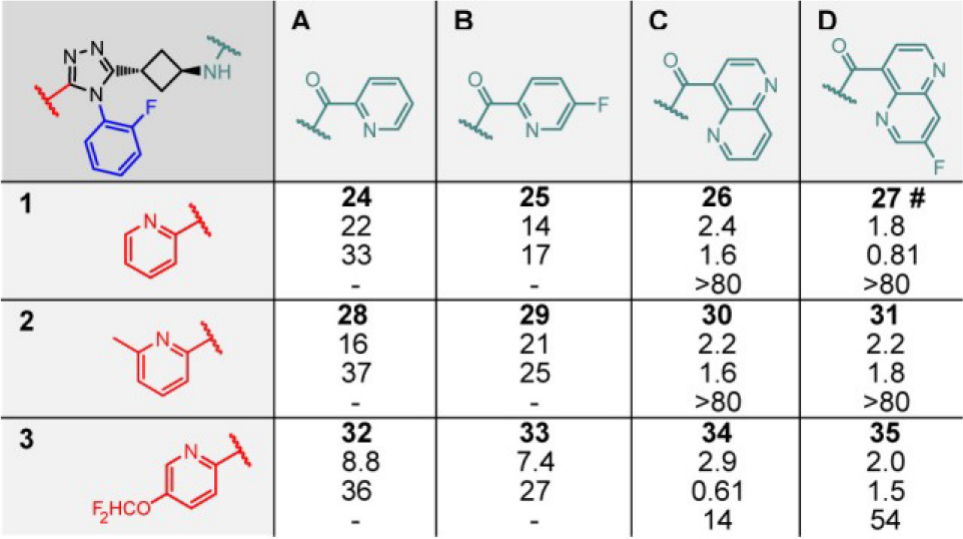
2D East (Green) and West Library (Red) with TNKS2 IC_50_ (nM), HEK293 IC_50_ (nM), and Solubility (μM) Values Depicted^a^

a # indicates that the compound was evaluated in a mouse pharmacokinetics analysis.

Since **1** displayed low solubility and high Caco-2 efflux,^11^ we substituted the benzimidazolone NH group by forming the *N*-Me variant **17**;^31^ however, this resulted in a 20-fold less efficacious compound (Figure 2a). Likewise, the oxygen-containing analogue **18** displayed decreased efficacy when compared to **1** (Figure 2a). We then replaced the benzimidazolone moiety,^10,20^ as this can inflict high efflux and low solubility by a series of East-positioned amides. From these amides, **20** and **21** turned out to be the most potent resulting in picomolar cellular inhibitory IC_50_ efficacies (Figure 2a). Solubility and Caco-2 cell permeation were completely restored in **21** (Figure 2b). The amide having the *N*-Me group (**44**) was inactive, whereas activity was restored in the cyclic version (**51**) (Supplementary Table 1a). Nonaromatic amides (**52**, **53**, and **54**) resulted in inactive compounds (Supplementary Table 1a).

To interrogate the West-side of the pharmacophore, pyridine and pyrimidine analogues were prepared. These compounds inhibited the cellular WNT/β-catenin signaling pathway reporter assay to a similar extent as lead **1**, except the 2-pyrimidyl substituted compound (**56**). The ethoxypyridyl derivative **22** was consequently selected as a starting point for further hybrid synthesis (Figure 2a and Supplementary Table 1b). Introduction of thiazoles to replace the six-membered heterocycle resulted in less efficacious compounds, with **59** displaying the most favorable properties in this cluster (Supplementary Table 1b). Introduction of aliphatic rings, such as cyclopentane in **63** and cyclopropane in **64**, indicated that aromatic ring systems are required in this position for maintaining potency (Supplementary Table 1b).

Next, in a series of synthesized South-aryl products, the 2-trifluoromethyl (**66**) showed comparable activities to the previous lead **1** (Supplementary Table 1b). The thiophenyl moeity as a bioisosteric replacement for the aryl group was less tolerated, while cycloalkyl replacements resulted in activities in the micromolar range (Supplementary Table 1c).

**Figure 3:**
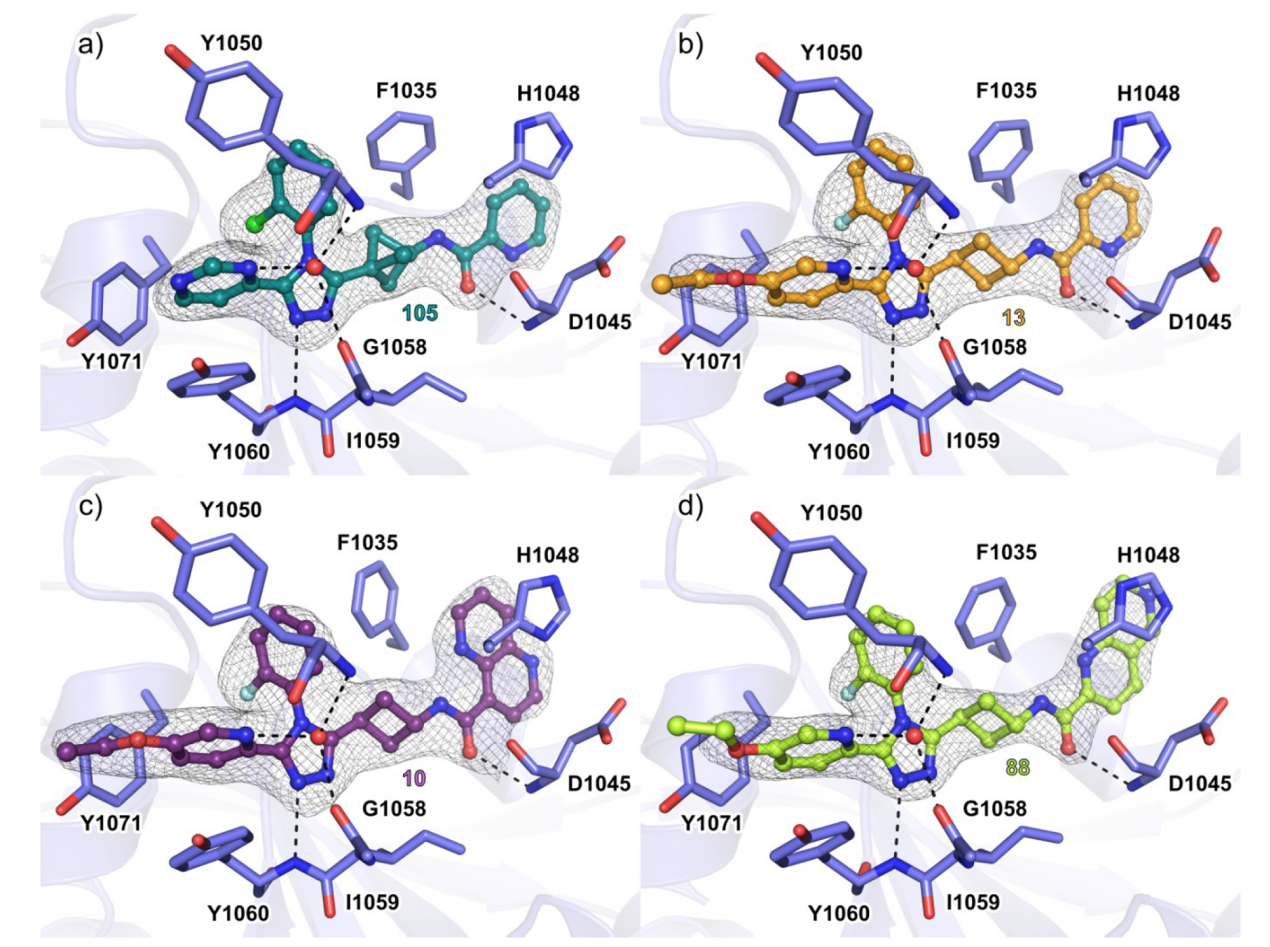
Cocrystal structures of TNKS2 with inhibitors. (a) Binding mode of **105** with TNKS2 catalytic domain (PDB code 6TKN). (b) Binding mode of **13** with TNKS2 catalytic domain (PDB code 6TG4). (c) Binding mode of **10** with TNKS2 catalytic domain (PDB code 6TKM). (d) Binding mode of **88** with TNKS2 catalytic domain (PDB code 6TKS). The dashed lines in black represent hydrogen bonds, and the red spheres represent water molecules. The σ_A_ weighted 2*F*_o_ – *F*_c_ electron density maps around the ligands are contoured at (1.4–1.7)σ. Structures were solved with molecular replacement using the structure of TNKS2 (PDB code 5NOB) as a starting model.

Linker variations were addressed with the *cis*-cyclobutyl linker and bicyclo[1.1.1]pentane linker **75** and **19**, respectively (Supplementary Table 1d). Compound **75** displayed decreased potency (Supplementary Table 1d), while compound **19** suffered from low solubility (Figure 2b). Despite the more rigid geometry of the bicyclo[1.1.1]pentane in comparison to *trans*-cyclobutyl of **1**, the cocrystal structure with TNKS2 showed a very similar binding mode at the NAD^+^ binding cleft (Figure 3). **105** occupied the adenosine subpocket and formed the typical hydrogen bonds to the backbone amides of Tyr1060 and Asp1045 (Figure 3a and Supplementary Figure 1a). A water molecule forms bridging interactions between the pyridine nitrogen and Gly1058 and Tyr1050, and the same applies to all the cocrystal structures described (Figure 3a).

**Figure 4:**
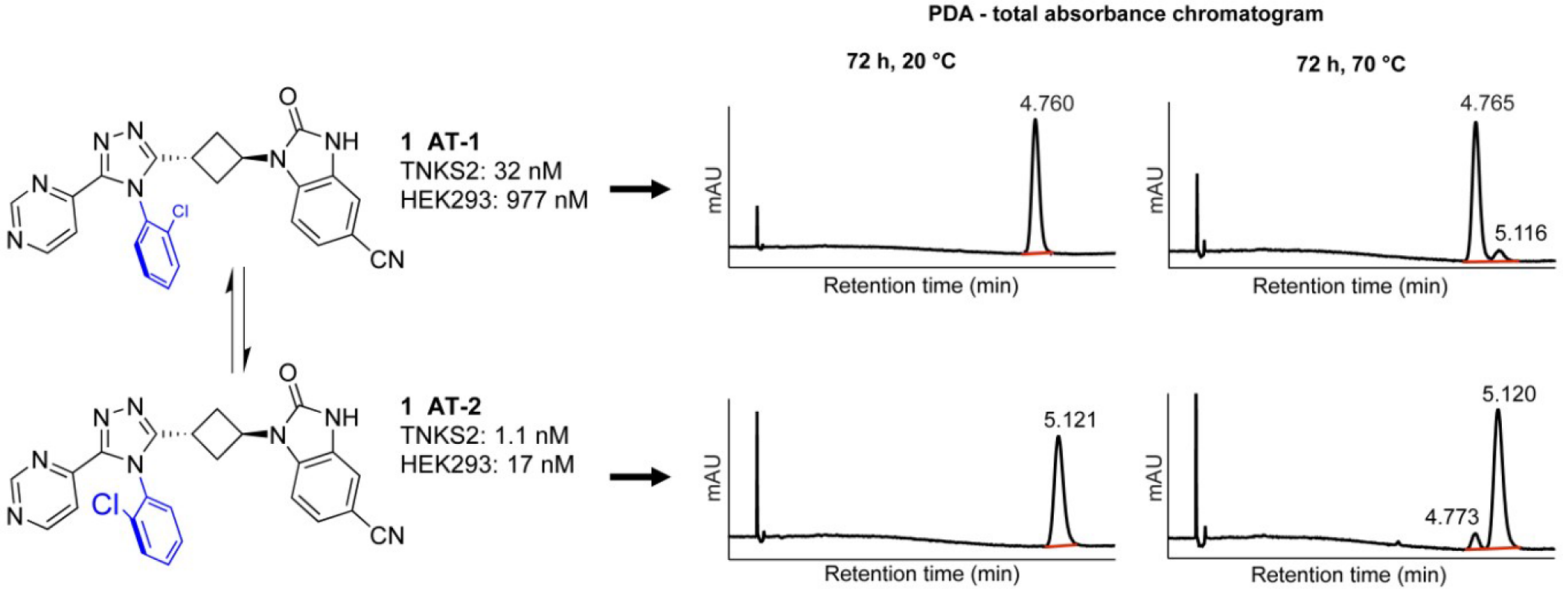
Atropisomerism of **1** (stereochemistry arbitrarily assigned) on chiral SFC and TNKS2 and HEK293 IC_50_ values.

Rotational isomerism (atropisomerism) is a known phenomenon for substituted triazoles and can potentially lead to complexity and challenges for the drug discovery and development processes as atropisomers might have differing biological activities toward a target, different off-target profiles, and different pharmacokinetic properties.^32,33^ Since **1** does not contain asymmetric centers, atropisomers are mirror images (enantiomers). Hence, on an achiral HPLC column, as well as in NMR, such atropisomers are indiscernible. In contrast, on a chiral SFC column, lead **1** showed two signals indicating rotational isomerism (Figure 4). When separated, these isomers did not interconvert at 20 °C for 72 h but showed a minor interconversion at 70 °C during 72 h (Figure 4). Interestingly, both isomers, **1**-AT-1 and **1**-AT-2, differed in potency and efficacy with a factor of 30 to almost 60, respectively (Figure 4). To investigate whether atropisomerism was induced by the South 2-chlorophenyl substituent, we analyzed all synthesized compounds with chiral SFC. All compounds with a South 2-chlorophenyl group showed atropisomers, while compounds without this group, including the symmetric 2,6-dichlorophenyl **67**, did not (Supplementary Table 1c). In addition, **66**, containing a bulky 2-trifluoromethyl group, also showed two signals on a chiral SFC column. No rotamers were observed for the 2-fluorophenyl South group at room temperature. Hence, this group was considered a viable substitution to avoid atropisomerism. As a consequence, in the following optimization campaign, the South 2-chlorophenyl moiety was replaced with the 2-fluorophenyl group of **23** resulting in acceptable efficacy compared to other substitutions (Figure 2a and Supplementary Table 1c).

The comparison of the binding modes of the 2-halogen-substituted compounds showed that the chlorine or fluorine atoms were pointing into the same direction in the TNKS2 active site (Figure 3 and Supplementary Figure 1b–e). This is in agreement with the clear difference in the measured potencies of the atropisomers (Figure 4). The South moiety without a halogen was less potent, had similar orientation in the binding pocket, but caused a conformational change in the Tyr1050 covering the active site (Supplementary Figure 1d).

Next, all possible hybrid combinations were synthesized employing the East moieties of compounds **20** and **21**, a South 2-fluorophenyl group, and three different West moieties revisiting the 2-pyridyl-4-methylsulfonyl moiety as well (Table 1).^9^ From these six molecules, compounds with the 1,5-naphthyridine moiety (**10**, **11**, and **12**) showed approximately 30-fold improved cellular inhibitory efficacy compared to their counterparts with the 2-pyridyl moiety (**13**, **14**, and **15**, respectively, Table 1). The binding mode of compound **13** in cocrystal structures was similar, and the hydrogen bonds to the backbone amides seen in the **1** cocrystal structures were retained (Figure 3 and Supplementary Figure 1b). The large East moieties 1,5- and 1,6-naphthyridine (e.g., see **87**, **88**, **106**, and **107**Supplementary Figure 4a) appeared to form a more efficient π–π-stacking interaction with His1048 and a hydrophobic interaction with Phe1035, causing the side chain to gradually rotate according to the form of the East moiety (Figure 3c,d, Supplementary Figure 1c–e, Supplementary Table 2). However, compounds with this naphthyridine moiety were less stable in microsomes relative to the corresponding East-pyridines, and consequently, **13** was short-listed for mouse pharmacokinetics studies. In addition, the quinoline-containing **16** displayed similar inhibition in the cellular WNT reporter assay compared to **12** and also possessed improved calculated physical–chemical properties (ChemAxon, cLogP = 3.0/3.7, tPSA = 128/141 for **16** and **12**, respectively) (Figure 2a and Table 1). On the basis of these results, **16** was short-listed for mouse pharmacokinetics analysis. Further optimization focused on pyridine-type West-side variations and discarded the West 2-thiazole (**11**) because of adverse solubility and low efficacy (**14**) (Table 1). In an East and West 2D library, fluorinated analogues of the 2-pyridyl and the 1,5-naphthyridine were introduced (Table 2 and Supplementary Table 2). From these compounds, **27** was short-listed for mouse pharmacokinetics analysis.

Compared to the initial benchmark lead compound **1**, the peroral mouse pharmacokinetics data of the selected and short-listed compounds showed significantly improved profiles exhibiting lower clearance and volume of distribution and 15–35 times higher exposure (Figure 2c). In due course of the study, **13** had been further characterized including selectivity toward other members of the PARP family, structural analysis of its binding mode, kinetic solubility, Caco-2 permeability and efflux, CYP3A4 inhibition, mouse plasma stability, mouse plasma protein binding, hERG inhibition, Ames test, and off-target safety panel, exhibiting overall favorable parameters (Figure 3, Table 3, Supplementary Table 3 and Supplementary Figure 2).

**Table 3:** Profiling of **1** and **13**^a^

parameter	**1**	**13**
MW (g/mol)	468.91	458.50
ALogP (LiveDesign 8.6)	4.7	3.6
AlogD (LiveDesign 8.6)	5.3	4.0
tPSA (LiveDesign 8.6) (Å^2^)	118.1	94.8
TNKS1 (IC_50_, nM)	29	127
TNKS2 (IC_50_, nM)	6.3	14
PARPs/ARTDs (IC_50_, μM), ARTD1/2/3/4/7/8/10/12	>10	>10
HEK293 cells (IC_50_, nM)	19	19
kinetic solubility, PBS, pH = 7 (μM)	12	95.7
Caco-2 A–B: *P*_ap__p_ (10^–6^ cm/s)	10.0	39.5
Caco-2 efflux ratio	4.17	0.610
microsomal stability, human/mouse, Cl_mt_ ((μL/min)/mg protein)	242/204	<15/37
hepatocyte stability, human/mouse/dog/rat/cynomolgus, Cl_int_ ((μL/min)/10^6^ cells)	9.8/ND/ND/ND/ND	<0.1/28/<0.1/3.8/<0.1
CYP3A4 inhibition (IC_50_, μM)	1.26	>25
mouse plasma stability *t*_1/2_ (min)		880
mouse PPB (%)		93.92
hERG inhibition (IC_50_, μM)		>25
Ames test		nongenotoxic
Cerep safety panel, 44 targets (10 μM)		clean (A2A, 53% inhib)
bioavailability *F* (%)	47	107
po PK mouse *t*_1/2_ (h)	1.5	0.67
po PK mouse Cl ((L/h)/kg)	34.0	2.09
po PK mouse Vd (L/kg)	74.2	2.03
po PK mouse AUC_0→*t*_	145	2384

a ND = not determined.

**Figure 5:**
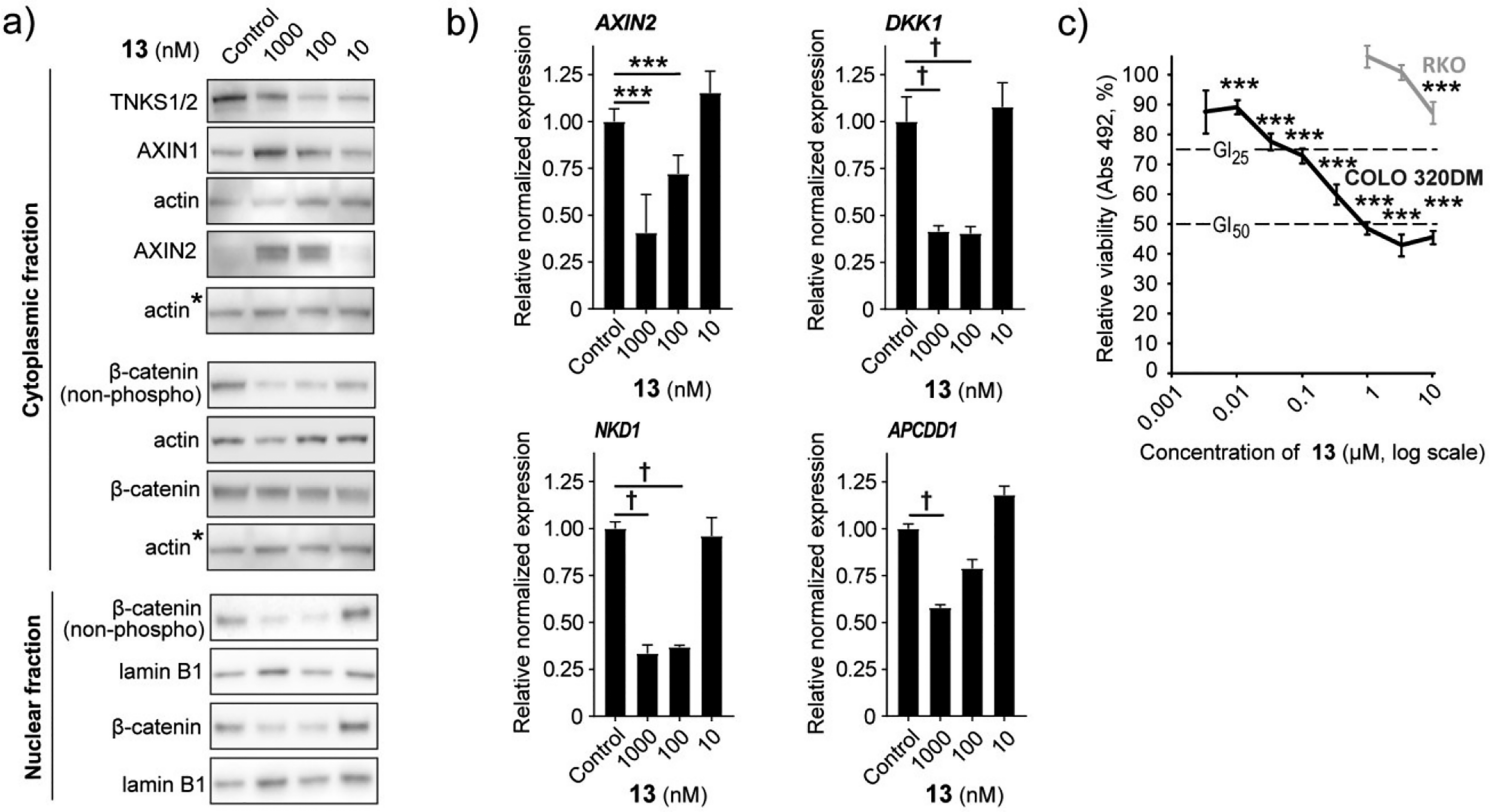
**13** can inhibit WNT/β*-*catenin signaling activity and act as an antiproliferative agent in COLO 320DM cells. (a) Representative immunoblots of cytoplasmic TNKS1/2, AXIN1, AXIN2 and cytoplasmic and nuclear transcriptionally active β*-*catenin (non-phospho) and β-catenin. Actin or lamin B1 document equals protein loading, and ∗ indicates that the same actin immunoblot is used as loading control for both AXIN2 and β*-*catenin. For (a) and (b), control = 0.01% DMSO. (b) Real-time RT-qPCR analyses of WNT/β*-*catenin signaling target genes (*AXIN2*, *DKK1*, *NKD1*, and *APCDD1*). For (b) and (c), ANOVA tests (Holm–Sidak method, versus control) are indicated by ∗∗∗ (*P* < 0.001), and ANOVA on ranks tests (Tukeys test versus control) are indicated by † (*P* < 0.05). Mean values ± standard deviations for combined data from a minimum of three independent experiments with three replicates each are shown. (c) MTS colorimetric cell growth assay for various doses of 13 in APC^mutated^ COLO 320DM (black) and APC^wild-type^ RKO (gray) cells. Control = 0. 1% DMSO. Dotted lines depict 50% (GI_50_) and 25% (GI_25_) growth inhibition levels. Mean values ± standard deviations for one representative experiment out of three independent experiments are shown.

Tankyrase inhibition can context-dependently antagonize proliferation and viability in cancer cell lines *in vitro* and *in vivo*, including in the colorectal adenocarcinoma cell line COLO 320DM harboring WNT/β*-*catenin signaling-inducing *APC* mutations.^5,22^ Hence, cultured COLO 320DM cells were treated with various doses of **13** to evaluate the efficacy in reducing canonical WNT/β*-*catenin signaling and the potential as an antiproliferative agent. As expected for a potent tankyrase inhibitor, treatment with **13** reduced TNKS1/2 protein levels, stabilized AXIN1 and AXIN2 proteins, and reduced the level of transcriptionally active β*-*catenin (nonphoshorylated) in both the cytoplasmic and nuclear fraction (Figure 5a and Supplementary Figure 3a,b). Administration of **13** also decreased transcription of the WNT/β*-*catenin signaling target genes *AXIN2*, *DKK1*, *NKD1*, and *APCDD1* (Figure 5b). Moreover, **13** exposure decreased proliferation and viability in COLO 320DM cells (GI_50_ = 650 nM and GI_25_ = 94 nM), while control *APC*^wild-type^ RKO colorectal cancer cells were only modestly affected by the treatment at a 10 μM compound concentration (Figure 5c). In conclusion, these results authenticate that **13** can both potently and specifically inhibit WNT/β*-*catenin signaling activity and act as an antiproliferative agent in COLO 320DM cells.

## Conclusion

In summary, through a systematic building-block-based and crystallography-guided structure–activity-relationship analysis, we identified novel 1,2,4-triazole-based optimized lead tankyrase inhibitors with low nanomolar and picomolar IC_50_ activities in a WNT/β*-*catenin signaling cellular reporter assay. The adverse chemical properties of the preceding lead compound **1**,^11^ displaying atropisomerism and solubility liabilities, were excluded in the here identified advanced lead compound **13**. Compound **13** shows high selectivity toward TNKS1/2, an overall favorable ADME, including highly improved Caco-2 permeability and microsomal stability, a clean off-target safety profile, a >15-fold increased exposure in a mouse pharmacokinetics analysis, and a robust inhibition of WNT/β*-*catenin signaling and proliferation in the colon cancer cell line COLO 320DM. Our work provides a considerably optimized compound for targeting TNKS1/2 and WNT/β*-*catenin signaling in cancer and other disease models.

## Experimental Section

### General Methods

All starting materials and dry solvents were commercially obtained. The synthesis of hydrazide **2** was scaled up employing a similar procedure reported in our earlier publication.^11^ Reactions were performed under an inert atmosphere of nitrogen when necessary. Microwave reactions were carried out in sealed vials. Column chromatography was carried out on silica gel cartridges (40 μm irregular), and TLC analysis was performed on silica gel 60 F_254_ plates.

### NMR

NMR spectra were recorded in chloroform-*d*, unless otherwise stated, on a 400 MHz spectrometer with tetramethylsilane as internal standards. Coupling constants are given in Hz. Peaks are reported as singlet (s), doublet (d), triplet (t), quartet (q), quintet (p), sextet (h), septet (hept), multiplet (m), or a combination thereof. br stands for broad.

### LC/MS

LC/MS chromatograms mass spectra were recorded using electrospray ionization in positive or negative ionization mode on Agilent 1260 Bin: pump, G1312B, degasser; autosampler; ColCom; DAD G1315C; MSD G6130B ESI; eluent A, acetonitrile; eluent B, 10 mM ammonium bicarbonate in water (base mode) or 0.1% formic acid in water (acid mode).

### HRMS

HRMS spectra were recorded with a LC-MS Q Exactive Focus spectrometer calibrated with the Pierce calibration solution both in positive and negative modes.

### GC/MS

Agilent 6890N, injection S/SL; injector 7683. MS: 5973 MS, EI-positive; carrier gas He.

### Analytical SFC

Waters UPC2, Bin pump ACQ-ccBSM; autosampler, column manager, PDA ACQ-PDA; QDA and isocratic pump ACQ-ISM; eluent A, CO_2_; eluent B, MeOH + 20 mM ammonia.

### Preparative SFC

Waters Prep 100 SFC UV/MS directed system; Waters 2998 photodiode array (PDA) detector; Waters Acquity QDa MS detector; Waters 2767 sample manager. Columns: Phenomenex Lux Amylose-1 (250 mm × 21 mm, 5 μm), Phenomenex Lux Cellulose-1 (250 mm × 21.2 mm, 5 μm), Phenomenex Lux Cellulose-2 (250 mm × 21.2 mm, 5 μm), Diacel Chiralpak IC for SFC (250 mm × 20 mm, 5 μm); column temp 35 °C; flow 70 mL/min; ABPR 120 bar; eluent A, CO_2_; eluent B, 20 mM ammonia in methanol. Linear gradient: *t* = 0 min 10% B, *t* = 5 min 50% B; *t* = 7.5 min 50% B. Detection: PDA (210–400 nm). Fraction collection is based on PDA TIC.

### Analytical SFC

Waters UPC2, Bin pump ACQ-ccBSM; autosampler, column manager; PDA ACQ-PDA; QDA and isocratic pump ACQ-ISM. Phenomenex Amylose-1 (100 mm × 4.6 mm, 5 μm); column temp 35 °C; flow 2.5 mL/min; BPR 170 bar; eluent A, CO_2_; eluent B, MeOH + 20 mM ammonia. Linear gradient: *t* = 0 min 5% B, *t* = 5 min 50% B; *t* = 6 min 50% B. Detection: PDA (210–320 nm).

### MPLC

Preparative base XSelect. Instrument type: Reveleris prep MPLC; column, Waters XSelect CSH C18 (145 mm × 25 mm, 10 μm); flow 40 mL/min; column temp, room temperature; eluent A, 10 mM ammonium bicarbonate in water (pH = 9.0); eluent B, 99% acetonitrile + 1% 10 mM ammonium bicarbonate in water. Gradient: *t* = 0 min 5% B, *t* = 1 min 5% B, *t* = 2 min 30% B, *t* = 17 min 70% B, *t* = 18 min 100% B, *t* = 23 min 100% B. Detection UV: 220, 254, 340 nm.

### MPLC

Preparative acid Luna. Instrument type: Reveleris prep MPLC; column, Phenomenex Luna C18(3) (150 mm × 25 mm, 10 μm); flow 40 mL/min; column temp, room temperature; eluent A, 0.1% (v/v) formic acid in water; eluent B, 0.1% (v/v) formic acid in acetonitrile. Gradient: *t* = 0 min 5% B, *t* = 1 min 5% B, *t* = 2 min 30% B, *t* = 17 min 70% B, *t* = 18 min 100% B, *t* = 23 min 100% B. Detection UV: 220, 254, 340 nm, ELSD.

All test compounds were found to be >95% pure by LCMS and H NMR analysis. Intermediates in the synthesis were >95% pure unless stated otherwise.

### 3-(*trans*-3-(5-(5-Ethoxypyridin-2-yl)-4-(2-fluorophenyl)-4*H*-1,2,4-triazol-3-yl)cyclobutyl)-1,5-naphthyridine-4-carboxamide (**10**)

The title compound was prepared according to general procedure F as a white solid (21.4 mg, 38% yield). LC/MS (ESI) *m*/*z* for C_28_H_24_FN_7_O_2_ 509 (calcd) 510 ([M + H]^+^, found). ^1^H NMR (400 MHz, chloroform-*d*) δ 11.31 (d, *J* = 5.9 Hz, 1H), 9.14 (d, *J* = 4.4 Hz, 1H), 8.98 (dd, *J* = 4.2, 1.8 Hz, 1H), 8.58–8.52 (m, 2H), 8.17 (d, *J* = 8.8 Hz, 1H), 7.88 (d, *J* = 2.9 Hz, 1H), 7.74 (dd, *J* = 8.6, 4.2 Hz, 1H), 7.48–7.40 (m, 1H), 7.26–7.14 (m, 4H), 4.84 (h, *J* = 6.9 Hz, 1H), 4.04 (q, *J* = 7.0 Hz, 2H), 3.53 (tt, *J* = 10.3, 5.6 Hz, 1H), 3.14–3.02 (m, 2H), 2.62–2.48 (m, 2H), 1.40 (t, *J* = 7.0 Hz, 3H).

### 3-(*trans*-3-(4-(2-Fluorophenyl)-5-(thiazol-2-yl)-4*H*-1,2,4-triazol-3-yl)cyclobutyl)-1,5-naphthyridine-4-carboxamide (**11**)

The title compound was prepared according to general procedure F as a white solid (6.6 mg, 23% yield). LC/MS (ESI) *m*/*z* for C_24_H_18_FN_7_OS 471 (calcd) 472 ([M + H]^+^, found). ^1^H NMR (400 MHz, chloroform-*d*) δ 11.33 (d, *J* = 6.0 Hz, 1H), 9.15 (d, *J* = 4.5 Hz, 1H), 8.98 (dd, *J* = 4.2, 1.8 Hz, 1H), 8.59–8.52 (m, 2H), 7.75 (dd, *J* = 8.5, 4.2 Hz, 1H), 7.65 (d, *J* = 3.2 Hz, 1H), 7.57–7.47 (m, 1H), 7.37 (d, *J* = 3.2 Hz, 1H), 7.34–7.22 (m, 3H), 4.92–4.80 (m, 1H), 3.60–3.49 (m, 1H), 3.15–3.02 (m, 2H), 2.68–2.51 (m, 2H).

### 3-(*trans*-3-(4-(2-Fluorophenyl)-5-(5-(methylsulfonyl)pyridin-2-yl)-4*H*-1,2,4-triazol-3-yl)cyclobutyl)-1,5-naphthyridine-4-carboxamide (**12**)

The title compound was prepared according to general procedure F as a white solid (14.8 mg, 53% yield). LC/MS (ESI) *m*/*z* for C_27_H_22_FN_7_O_3_S 543 (calcd) 544 ([M + H]^+^ found). ^1^H NMR (400 MHz, chloroform-*d*) δ 11.31 (br d, *J* = 6.1 Hz, 1H), 9.15 (d, *J* = 4.5 Hz, 1H), 8.97 (dd, *J* = 4.2, 1.8 Hz, 1H), 8.73 (dd, *J* = 2.5, 0.8 Hz, 1H), 8.57 (pseudo d, *J* = 1.6 Hz, 1H), 8.55 (pseudo d, *J* = 4.2 Hz, 2H), 8.29 (dd, *J* = 8.3, 2.3 Hz, 1H), 7.74 (dd, *J* = 8.5, 4.2 Hz, 1H), 7.55–7.46 (m, 1H), 7.30–7.19 (m, 3H), 4.89 (h, *J* = 7.1 Hz, 1H), 3.55 (tt, *J* = 10.0, 5.5 Hz, 1H), 3.17–3.02 (m, 2H), 3.08 (s, 3H), 2.69–2.52 (m, 2H).

### 3-(*trans*-3-(5-(5-Ethoxypyridin-2-yl)-4-(2-fluorophenyl)-4*H*-1,2,4-triazol-3-yl)cyclobutyl)picolinamide (**13**)

The title compound was prepared according to general procedure F as a white solid (16.8 mg, 46% yield). LC/MS (ESI) *m*/*z* for C_25_H_23_FN_6_O_2_ = 458 (calculated), 459 ([M + H]^+^, found). ^1^H NMR (400 MHz, chloroform-*d*) δ 8.53 (dt, *J* = 4.7, 1.3 Hz, 1H), 8.21 (d, *J* = 7.0 Hz, 1H), 8.19–8.12 (m, 2H), 7.88 (d, *J* = 2.8 Hz, 1H), 7.83 (td, *J* = 7.7, 1.7 Hz, 1H), 7.48–7.38 (m, 2H), 7.24–7.15 (m, 4H), 4.76 (h, *J* = 7.0 Hz, 1H), 4.04 (q, *J* = 6.9 Hz, 2H), 3.47 (tt, *J* = 10.0, 5.1 Hz, 1H), 3.09–2.97 (m, 2H), 2.52–2.36 (m, 2H), 1.40 (t, *J* = 7.0 Hz, 3H). HRMS 459.19393 ([M + H]^+^, calculated), 459.19353 ([M + H]^+^, found), Δ = −0.87 ppm.

### 3-(*trans*-3-(4-(2-Fluorophenyl)-5-(thiazol-2-yl)-4*H*-1,2,4-triazol-3-yl)cyclobutyl)picolinamide (**14**)

The title compound was prepared according to general procedure F as a white solid (21.3 mg, 84% yield). LC/MS (ESI) *m*/*z* for C_21_H_17_FN_6_OS 420 (calcd) 421 ([M + H]^+^, found). ^1^H NMR (400 MHz, chloroform-*d*) δ 8.53 (dq, *J* = 4.5, 0.9 Hz, 1H), 8.23 (br d, *J* = 7.0 Hz, 1H), 8.17 (dt, *J* = 7.8, 1.1 Hz, 1H), 7.84 (td, *J* = 7.7, 1.7 Hz, 1H), 7.64 (d, *J* = 3.2 Hz, 1H), 7.57–7.49 (m, 1H), 7.43 (ddd, *J* = 7.8, 4.7, 1.3 Hz, 1H), 7.37 (d, *J* = 3.2 Hz, 1H), 7.33–7.27 (m, 2H), 7.26–7.21 (m, 1H), 4.78 (h, *J* = 7.0 Hz, 1H), 3.54–3.43 (m, 1H), 3.11–2.96 (m, 2H), 2.56–2.39 (m, 2H). HRMS 421.12413 ([M + H]^+^, calculated), 421.12330 ([M + H]^+^, found), Δ = −0.84 ppm.

### 3-(*trans*-3-(4-(2-Fluorophenyl)-5-(5-(methylsulfonyl)pyridin-2-yl)-4*H*-1,2,4-triazol-3-yl)cyclobutyl)picolinamide (**15**)

The title compound was prepared according to general procedure F as a white solid (6.1 mg, 40% yield). LC/MS (ESI) *m*/*z* for C_24_H_21_FN_6_O_3_S 492 (calcd) 493 ([M + H]^+^ found). ^1^H NMR (400 MHz, chloroform-*d*) δ 8.72 (d, *J* = 2.3 Hz, 1H), 8.58–8.51 (m, 2H), 8.28 (dd, *J* = 8.4, 2.3 Hz, 1H), 8.23 (br d, *J* = 7.0 Hz, 1H), 8.17 (dt, *J* = 7.7, 1.1 Hz, 1H), 7.84 (td, *J* = 7.7, 1.7 Hz, 1H), 7.55–7.47 (m, 1H), 7.43 (ddd, *J* = 7.6, 4.8, 1.2 Hz, 1H), 7.29–7.17 (m, 3H), 4.80 (h, *J* = 7.1 Hz, 1H), 3.53–3.44 (m, 1H), 3.07 (s, 3H), 3.14–2.97 (m, 2H), 2.57–2.40 (m, 2H).

### 3-(*trans*-3-(4-(2-Fluorophenyl)-5-(5-(methylsulfonyl)pyridin-2-yl)-4*H*-1,2,4-triazol-3-yl)cyclobutyl)quinoline-8-carboxamide (**16**)

The title compound was prepared according to general procedure F as a white solid (24.9 mg, 91% yield). LC/MS (ESI) *m*/*z* for C_28_H_23_FN_6_O_3_S = 542 (calculated), 543 ([M + H]^+^ found). ^1^H NMR (400 MHz, chloroform-*d*) δ 11.56 (d, *J* = 5.8 Hz, 1H), 8.91 (dd, *J* = 4.3, 1.8 Hz, 1H), 8.83 (dd, *J* = 7.4, 1.6 Hz, 1H), 8.73 (d, *J* = 2.3 Hz, 1H), 8.56 (d, *J* = 8.4 Hz, 1H), 8.28 (dt, *J* = 8.4, 2.5 Hz, 2H), 7.96 (dd, *J* = 8.1, 1.6 Hz, 1H), 7.67 (t, *J* = 7.7 Hz, 1H), 7.49 (dt, *J* = 8.3, 5.0 Hz, 2H), 7.26–7.18 (m, 3H), 4.85 (apparent dq, *J* = 13.4, 6.8 Hz, 1H), 3.57 (apparent tt, *J* = 10.1, 5.8 Hz, 1H), 3.16–3.01 (m, 2H), 3.08 (s, 3H), 2.67–2.50 (m, 2H). HRMS 543.16091 ([M + H]^+^, calculated), 543.16007 ([M + H]^+^, found), Δ = −0.84 ppm.

### 3-(*trans*-3-(4-(2-Chlorophenyl)-5-(pyrimidin-4-yl)-4*H*-1,2,4-triazol-3-yl)cyclobutyl)-2-oxo-2,3-dihydrobenzo[*d*]oxazole-6-carbonitrile (**18**)

Crude hydroxybenzonitrile (**8**) (10 mg, 0.014 mmol, ∼60% pure) was dissolved in DCM (dried, 3.0 mL) under a nitrogen atmosphere. Acetonitrile (anhydrous, 1.0 mL) was also added followed by CDI (11 mg, 0.070 mmol), and the mixture was stirred for 60 h at ambient temperature. The solvent was evaporated, and the residue was purified by preparative SFC. After freeze-drying the purified fractions from acetonitrile/water, 2.8 mg (42% yield) of a white powder of the target compound (**18**) was obtained. LC/MS (ESI) *m*/*z* for C_24_H_16_ClN_7_O_2_ = 469/471 (calculated), 470/472 ([M + H]^+^, found). ^1^H NMR (400 MHz, chloroform-*d*) δ 8.86–8.82 (m, 2H), 8.28 (dd, *J* = 5.3, 1.2 Hz, 1H), 7.60–7.50 (m, 3H), 7.51–7.42 (m, 2H), 7.30 (dd, *J* = 7.8, 1.4 Hz, 1H), 7.18 (d, *J* = 8.2 Hz, 1H), 5.20 (p, *J* = 8.3 Hz, 1H), 3.44–3.34 (m, 2H), 3.34–3.25 (m, 1H), 2.98 (td, *J* = 8.5, 4.1 Hz, 1H), 2.93–2.83 (m, 1H).

### 1-(3-(4-(2-Chlorophenyl)-5-(pyrimidin-4-yl)-4*H*-1,2,4-triazol-3-yl)bicyclo[1.1.1]pentan-1-yl)-2-oxo-2,3-dihydro-1*H*-benzo[*d*]imidazole-5-carbonitrile (**19**)

Under a nitrogen atmosphere an impure batch of 3-amino-4-((3-(4-(2-chlorophenyl)-5-(pyrimidin-4-yl)-4*H*-1,2,4-triazol-3-yl)bicyclo[1.1.1]pentan-1-yl)amino)benzonitrile **19b** (36 mg, 0.060 mmol, ∼75% pure; see Supporting Information) was dissolved in dry DCM (6.0 mL), and it was treated at ambient temperature with several portions of CDI (in total 9 equiv) over a period of 24 h until the complete conversion was reached. The mixture was evaporated to dryness and the residue was first flashed on a 12 g silica gel cartridge eluted with a gradient of methanol (0% via 3% to 10%) in DCM. Then it was submitted to purification by preparative SFC isolating 19.0 mg (65% yield) of a white powder. LC/MS (ESI) *m*/*z* for C_25_H_17_ClN_8_O = 480/482 (calculated), 481/483 ([M + H]^+^, found). ^1^H NMR (400 MHz, DMSO-*d*_6_) δ 11.36 (s, 1H), 8.95 (d, *J* = 5.3 Hz, 1H), 8.90 (d, *J* = 1.2 Hz, 1H), 8.23 (dd, *J* = 5.3, 1.3 Hz, 1H), 7.79 (dd, *J* = 7.8, 1.5 Hz, 1H), 7.76 (dd, *J* = 8.0, 1.2 Hz, 1H), 7.66 (td, *J* = 7.8, 1.6 Hz, 1H), 7.58 (td, *J* = 7.6, 1.3 Hz, 1H), 7.44 (dd, *J* = 8.3, 1.4 Hz, 1H), 7.40–7.30 (m, 2H), 2.61–2.52 (m-distorted q, 6H). HRMS 481.12866 ([M + H]^+^, calculated), 481.12796 ([M + H]^+^, found), Δ = −1.46 ppm.

### 3-(*trans*-3-(4-(2-Chlorophenyl)-5-(pyrimidin-4-yl)-4*H*-1,2,4-triazol-3-yl)cyclobutyl)picolinamide (**20**)

The title compound was prepared according to general procedure F and obtained as a white solid (41.1 mg, 94% yield). LC/MS (ESI) *m*/*z* for C_22_H_18_ClN_7_O = 431/433 (calculated), 432/434 ([M + H]^+^, found). ^1^H NMR (400 MHz, chloroform-*d*) δ 8.82 (s, 1H), 8.81 (d, *J* = 3.3 Hz, 1H), 8.53 (qd, *J* = 4.8, 0.6 Hz, 1H), 8.28 (dd, *J* = 5.3, 1.3 Hz, 1H), 8.23 (d, *J* = 6.6 Hz, 1H), 8.17 (d, *J* = 7.8 Hz, 1H), 7.84 (td, *J* = 7.7, 1.7 Hz, 1H), 7.54 (dd, *J* = 8.0, 1.5 Hz, 1H), 7.49 (td, *J* = 7.7, 1.6 Hz, 1H), 7.46–7.38 (m, 2H), 7.30 (dd, *J* = 7.8, 1.5 Hz, 1H), 4.86–4.72 (m, 1H), 3.49–3.36 (m, 1H), 3.11–3.00 (m, 2H), 2.53–2.38 (m, 2H). HRMS 432.13341 ([M + H]^+^, calculated), 432.13272 ([M + H]^+^, found), Δ = −1.61 ppm.

### 3-(*trans*-3-(4-(2-Chlorophenyl)-5-(pyrimidin-4-yl)-4*H*-1,2,4-triazol-3-yl)cyclobutyl)-1,5-naphthyridine-4-carboxamide (**21**)

The title compound was prepared according to general procedure F and obtained as a white solid (12.7 mg, 26% yield). LC/MS (ESI) *m*/*z* for C_25_H_19_ClN_8_O = 482/484 (calculated), 483/485 ([M + H]^+^, found). ^1^H NMR (400 MHz, chloroform-*d*) δ 11.32 (d, *J* = 5.8 Hz, 1H), 9.15 (d, *J* = 4.4 Hz, 1H), 8.98 (dd, *J* = 4.2, 1.7 Hz, 1H), 8.85–8.79 (m, 2H), 8.60–8.52 (m, 2H), 8.29 (dd, *J* = 5.3, 1.3 Hz, 1H), 7.75 (dd, *J* = 8.5, 4.2 Hz, 1H), 7.54 (dd, *J* = 7.9, 1.5 Hz, 1H), 7.48 (td, *J* = 7.7, 1.6 Hz, 1H), 7.42 (td, *J* = 7.6, 1.6 Hz, 1H), 7.32 (dd, *J* = 7.8, 1.5 Hz, 1H), 4.88 (h, *J* = 7.2 Hz, 1H), 3.49 (tt, *J* = 9.7, 5.4 Hz, 1H), 3.17–3.04 (m, 2H), 2.65–2.50 (m, 2H). HRMS 483.14431 ([M + H]^+^, calculated), 483.14350 ([M + H]^+^, found), Δ = −0.81 ppm.

### 3-(*trans*-3-(4-(2-Chlorophenyl)-5-(5-ethoxypyridin-2-yl)-4*H*-1,2,4-triazol-3-yl)cyclobutyl)-2-oxo-2,3-dihydro-1*H*-benzo[*d*]imidazole-5-carbonitrile (**22**)

The title compound was prepared according to general procedure D from (1*r*,3*r*)-3-(5-cyano-2-oxo-2,3-dihydro-1*H*-benzo[*d*]imidazol-1-yl)cyclobutane-1-carbohydrazide **2**(11) (60.2 mg, 0.222 mmol) and methyl *N-*(2-chlorophenyl)-5-ethoxypyridine-2-carbimidothioate **E2** (68 mg, 0.222 mmol) affording 49.9 mg (42% yield) of a white solid. LC/MS (ESI) *m*/*z* for C_27_H_22_ClN_7_O_2_ = 511 (calculated), 512 ([M + H]^+^, found). ^1^H NMR (400 MHz, chloroform-*d*) δ 8.69 (s, 1H), 8.17 (d, *J* = 8.8 Hz, 1H), 7.87 (d, *J* = 2.8 Hz, 1H), 7.51 (distorted dd, *J* = 7.8, 1.8 Hz, 1H), 7.46 (td, *J* = 7.6, 1.8 Hz, 1H), 7.44–7.38 (m, 2H), 7.31 (distorted dd, *J* = 7.7, 1.7 Hz, 2H), 7.25–7.18 (m, 2H), 5.28 (t, *J* = 8.4 Hz, 1H), 4.04 (q, *J* = 7.0 Hz, 2H), 3.45–3.29 (m, 3H), 3.00–2.84 (m, 2H), 1.40 (t, *J* = 7.0 Hz, 3H). HRMS 512.15963 ([M + H]^+^, calculated), 512.15900 ([M + H]^+^, found), Δ = −1.23 ppm.

### 3-(*trans*-3-(4-(2-Fluorophenyl)-5-(pyrimidin-4-yl)-4*H*-1,2,4-triazol-3-yl)cyclobutyl)-2-oxo-2,3-dihydro-1*H*-benzo[*d*]imidazole-5-carbonitrile (**23**)

The title compound was prepared according to general procedure D from (1*r*,3*r*)-3-(5-cyano-2-oxo-2,3-dihydro-1*H*-benzo[*d*]imidazol-1-yl)cyclobutane-1-carbohydrazide **2**(11) (43.9 mg, 0.162 mmol) and methyl *N-*(2-fluorophenyl)pyrimidine-4-carbimidothioate **E3** (40 mg, 0.162 mmol) as a white powder (14 mg, 19% yield). LC/MS (ESI) *m*/*z* for C_24_H_17_FN_8_O = 452 (calculated), 453 ([M + H]^+^, found). ^1^H NMR (400 MHz, chloroform-*d*) δ 8.87 (d, *J* = 1.4 Hz, 1H), 8.85 (d, *J* = 5.2 Hz, 1H), 8.71 (br s, 1H), 8.29 (dd, *J* = 5.3, 1.4 Hz, 1H), 7.61–7.53 (m, 1H), 7.43 (dd, *J* = 8.3, 1.5 Hz, 1H), 7.35–7.29 (m, 2H), 7.26–7.22 (m, 2H), 7.19 (d, *J* = 8.3 Hz, 1H), 5.27 (p, *J* = 8.7 Hz, 1H), 3.54–3.44 (m, 2H), 3.39 (apparent q, *J* = 10.3 Hz, 1H), 3.05–2.97 (m, 1H), 2.88–2.78 (m, 1H). HRMS 453.15821 ([M + H]^+^, calculated), 453.15769 ([M + H]^+^, found), Δ = −1.16 ppm.

### 3-(*trans*-3-(4-(2-Fluorophenyl)-5-(pyridin-2-yl)-4*H*-1,2,4-triazol-3-yl)cyclobutyl)-7-fluoro-1,5-naphthyridine-4-carboxamide (**27**)

The title compound was prepared according to general procedure F as a white solid (17.2 mg, 70% yield). LC/MS (ESI) *m*/*z* for C_26_H_19_F_2_N_7_O = 483 (calculated), 484 ([M + H]^+^, found). ^1^H NMR (400 MHz, chloroform-*d*) δ 10.83 (d, *J* = 6.0 Hz, 1H), 9.15 (d, *J* = 4.5 Hz, 1H), 8.91 (d, *J* = 2.8 Hz, 1H), 8.52 (d, *J* = 4.4 Hz, 1H), 8.26 (dt, *J* = 7.9, 1.0 Hz, 1H), 8.24–8.16 (m, 2H), 7.77 (td, *J* = 7.8, 1.8 Hz, 1H), 7.50–7.42 (m, 1H), 7.25–7.15 (m, 4H), 4.87 (ht, *J* = 6.8, 1.6 Hz, 1H), 3.53 (tt, *J* = 9.3, 5.6 Hz, 1H), 3.15–3.02 (sym. m, 2H), 2.63–2.47 (sym. m, 2H). HRMS 484.16919 ([M + H]^+^, calculated), 484.16833 ([M + H]^+^, found), Δ = −1.78 ppm.

### Preparation of 3-(*trans*-3-(4-(2-Chlorophenyl)-5-(pyrimidin-4-yl)-4*H*-1,2,4-triazol-3-yl)cyclobutyl)isoindolin-1-one (**51**)

2-Formylbenzoic acid (**9**) (33 mg, 0.22 mmol) was suspended in DCM (dry, 1.0 mL) under a nitrogen atmosphere, and a solution of *trans*-3-(4-(2-chlorophenyl)-5-(pyrimidin-4-yl)-4*H*-1,2,4-triazol-3-yl)cyclobutan-1-amine (**Ga**) (0.20 mmol) in acetonitrile (anhydrous, 1.0 mL) and acetic acid (13 μL, 0.22 mmol) were added. The resulting solution was stirred for 17 h at ambient temperature. Then, two portions of sodium triacetoxyborohydride (each of 85 mg, 0.40 mmol) were added and the mixture was stirred for 3 h at ambient temperature. Extra methanol was added (extra dry, 1.0 mL) for better dissolution. The mixture was then heated at 50 °C overnight for 40 h while two extra portions of sodium triacetoxyborohydride were added (each of 85 mg, 0.40 mmol). The mixture was evaporated to dryness, and the residue was quenched with water and a few mL of 1 N aqueous HCl. DCM was added, the aqueous phase was basified with aqueous sodium bicarbonate, and the crude product was extracted with DCM (three times). The extracts were dried over sodium sulfate, filtered, and evaporated to dryness. The crude material was purified on a 12 g silica gel cartridge eluted with a gradient of methanol (0% to 5%) in DCM. The product fraction was lyophilized from acetonitrile/water providing a white powder (34.1 mg, 38% yield) of the target compound. LC/MS (ESI) *m*/*z* for C_24_H_19_ClN_6_O = 442/444 (calculated) 443/445 ([M + H]^+^, found). ^1^H NMR (400 MHz, chloroform-*d*) δ 8.86–8.78 (m, 2H), 8.29 (dd, *J* = 5.3, 1.2 Hz, 1H), 7.81 (d, *J* = 7.4 Hz, 1H), 7.58–7.47 (m, 3H), 7.47–7.39 (m, 3H), 5.09 (p, *J* = 8.2 Hz, 1H), 4.44 (s, 2H), 3.42–3.31 (m, 1H), 2.98–2.80 (m, 4H).

### Separation of Atropisomers of **1** and Investigation of Their Interconversion

An amount of 40 mg of racemic **1**(11) (40 mg, 0.085 mmol) was separated into pure atropisomers by preparative chiral SFC (see Supporting Information) providing, respectively, 16.1 and 11.8 mg of off-white solids. Two sets of two samples dissolved in an acetonitrile/methanol mixture were prepared for each atropisomers. One set was heated in a reaction block at 70 °C, while the other was kept at ambient temperature. Samples were analyzed after 24, 48, and 72 h to check for the interconversion of atropisomers. After 24 h no interconversion was observed at ambient temperature, while at 70 °C 2.5% of the opposite isomers were detected in both samples. After 48 h of heating about 5–6% interconversion was observed at 70 °C. After 72 h, the isomers showed no interconversion at ambient temperature, whereas at 70 °C about 8–9% of the other atropisomer was observed by chiral SFC.

### Biochemical Assays

Human tankyrase active constructs for TNKS1 (residues 1030–1317) and TNKS2 (residues 873–1162) and other ARTD/PARP enzymes used in the assays were expressed and purified as previously described.^34,35^ The enzymatic assay is based on the measurement of unreacted NAD^+^, which is chemically converted into a fluorescent compound.^36^ The fluorescence intensity was measured with excitation/emission wavelengths of 372 and 444 nm, respectively, using Tecan Infinity M1000 Pro. The compounds were prepared in half log dilution series and the reactions were done in quadruplicates with protein and compound controls to exclude the effect of compound autofluorescence. All reactions were done at ambient temperature. 20 nM TNKS1 or 5 nM TNKS2 was incubated for 20 h in assay buffer (50 mM BTP, pH 7.0, 0.5 mM TCEP, 0.01% Triton-X100) with compound and 10 μM or 500 nM NAD^+^, respectively. 5 nM ARTD1/PARP1 or 20 nM ARTD2/PARP2 was incubated for 30 min with compound and 500 nM NAD^+^ in assay buffer (50 mM Tris, pH 8.0, 5 mM MgCl_2_, 10 μg/mL activated DNA) supplemented with 0.2 or 0.1 mg/mL BSA, respectively. For ARTD3/PARP3, 20 nM enzyme was incubated for 4 h with compound and 500 nM NAD^+^ in assay buffer (50 mM PIPES, pH 7.0, 5 mM MgCl_2_, 20 μg/mL activated DNA, 0.2 mg/mL BSA). The assay conditions for the other ARTD enzymes were used as previously described.^37^

### WNT/β-Catenin in Signaling Reporter Assay

The luciferase-based WNT/β*-*catenin signaling pathway reporter assay in human HEK293 cells, as well as IC_50_ and GI_50_/GI_25_ calculations, was performed as previously described.^9^

### In vitro ADME assays

Kinetic solubility assay was performed following standard protocols of Mercachem. The following assays were performed according to the standard protocols of Cyprotex: Caco-2 permeability, dog microsomal stability, hepatocyte stability (human, mouse, dog, rat, and cynomolgus), CYP3A4 inhibition, mouse plasma stability, mouse plasma protein binding, and Ames test.

### Microsomal Stability Assay

Test compounds in DMSO (10 mM) were further diluted to 100 μM in acetonitrile. Human or mouse liver microsomes (BioIVT) from selected species are incubated in duplicate with the test compound at a final concentration of 1 μM in 0.1 M potassium phosphate buffer (pH 7.4) containing 3.3 mM MgCl_2_, 0.5 mg/mL microsomal protein, in the presence or absence of NADPH (1 mM). Incubations were performed at 37 °C in a total volume of 500 μL. Control incubations with reference substances were included for each experiment. At *t* = 0, 5, 15, 30, 45 min, an amount of 50 μL of the incubation mixture was transferred to a quench plate containing acetonitrile and internal standard (200 nM labetalol) cooled at 4 °C. After the last time point, the quench plates are mixed thoroughly and centrifuged for 15 min at 3700 rpm and 10 °C (Eppendorf 5804R). The supernatant was transferred to a 96-well plate and analyzed by LCMS (Vanquish Horizon UHPLC equipped with a diode array detector coupled to a Q Exactive focus hybrid quadrupole-Orbitrap mass spectrometer). The metabolic stability is evaluated by plotting the natural logarithm of the percentage test compound remaining versus time and performing linear regression.

### Safety Panel and hERG Inhibition

Inhibition of 44 selected targets (*n* = 2) including hERG (SafetyScreen 44) using 10 μM **13** was performed by Eurofins (Cerep-Panlabs).

### Mouse Pharmacokinetical Analysis

The pharmacokinetical analyses in mice were performed according to the standard protocols of Medicilon and, as previously described,^9^ using 3 animals per treatment group using 5% DMSO, 50% PEG400 (both Sigma-Aldrich) and 45% saline as vehicle.

### Crystallography

Compounds **105**, **13**, **10**, **106**, **107**, **87**, **88** (Supplementary Table 4) were cocrystallized with the catalytic domain of human TNKS2 (residues 952–1161) in the presence of chymotrypsin (1:100) based on crystallization efforts previously described.^11,20^ Protein (0.2 mM, 5.6 mg/mL) was mixed with 0.4 mM compound from a 10 mM DMSO stock solution. The crystallization was set up at 22 °C using sitting-drop vapor diffusion method by mixing 200 nL of protein with 100 nL of precipitant solution (0.1 M Bicine, pH 8.5–9.0, 7.5–25% PEG6000). Rod-shaped crystals appeared within 24 h and were cryoprotected using the precipitant solution containing 25% PEG6000 and 20% glycerol. Data were collected at ESRF Grenoble on beamlines ID30B, ID23-1, and ID30A-1 or at Diamond Light Source on beamline I04. Diffraction data were processed using the XDS package.^38^ All structures were solved using molecular replacement with PHASER^39^ using the structure of TNKS2 (PDB code 5NOB) as a starting model. Coot^40^ and Refmac5^41^ were used for model building and refinement, respectively. The images of the structures were prepared using PyMOL (The PyMOL Molecular Graphics System, version 1.8.4.0, Schrödinger, LLC.).

### Western Blot Analysis

Western blot analysis of nuclear and cytoplasmic lysates from compound-treated COLO 320DM cells was performed as previously described^26^ using the following primary antibodies: tankyrase-1/2 (TNKS1/2, E-10, sc-365897, Santa Cruz Biotechnology), AXIN1 (C7B12, 3323, Cell Signaling Technology), AXIN2 (76G6, 2151, Cell Signaling Technology), non-phospho (active) β-catenin (D13A1, 8814, Cell Signaling Technology), β-catenin (610153,1:500, BD Biosciences). Actin (A2066, Sigma-Aldrich) and lamin B1 (ab16048, Abcam) were used as loading controls. Primary antibodies were visualized with HRP-conjugated secondary antibodies (mouse anti-rabbit IgG, sc-2357, Santa Cruz Biotechnology or donkey anti-rabbit IgG, 711-035-152, Jackson ImmunoResearch) using ChemiDoc Touch Imaging System (Bio-Rad).

### RNA Isolation and Real-Time qRT-PCR

RNA isolation and real-time qRT-PCR were performed as previously described^26^ using the following probes (all from Applied Biosystems): AXIN2 (Hs00610344_m1), DKK1 (Hs00183740_m1), NKD1 (Hs01548773_m1), APCDD1 (Hs00537787_m1), and GAPDH (Hs02758991_g1).

### Proliferation Assay

1000 COLO 320DM cells per well were seeded in 96-well plates. The day after, cell culture medium was changed to contain various doses of **13** (*n* = 4) or vehicle (DMSO, Sigma-Aldrich) and the plates were incubated at 37 °C for 5 days. The cells were incubated for 1 h at 37 °C with CellTiter 96 AQueous nonradioactive cell proliferation assay (MTS, Promega) according to the supplier’s recommendations. Abs_490_ was measured (Wallac 1420 Victor2 microplate reader, PerkinElmer) and compared to Abs_490_ (*t*_0_) using the following formula to determine single well values relative to the DMSO vehicle control: (sample *A*_490_ – average *A*_490_*t*_0_) × 100/(average *A*_490_ [for 0.01% DMSO controls] – average *A*_490_*t*_0_).

## Supporting Information Available

General and specific synthetic procedures and spectra (PDF)
Additional figures and tables for inhibition data and crystallography (PDF)
Molecular formula strings and some data (CSV)

## Accession Codes

Atomic coordinates and structure factors have been deposited to Protein Data Bank with accession codes 6TG4, 6TKM, 6TKN, 6TKP, 6TKQ, 6TKR, and 6TKS.

## Abbreviations Used

ADME: absorption, distribution, metabolism, and excretion
ADP: adenosine 5′-diphosphate
AKT: serine/threonine kinase
AMOT: angiomotin
AMP: adenosine monophosphate
AMPK: AMP-activated protein kinase
APC: adenomatous polyposis coli
APCDD1: APC down-regulated 1
ARTD: diphtheria toxin-like ADP-ribosyltransferase
AT: atropisomer
AUC: area under the curve
AXIN: axis inhibition protein
Boc: *tert*-butyloxycarbonyl
CDI: *N*,*N*′-carbonyldiimidazole
Cl: clearance
DIPEA: diisopropylethylamine
DKK1: dickkopf WNT signaling pathway inhibitor 1
DMA: *N*,*N*-dimethylacetamide
DMF: *N*,*N*-dimethylformamide
DMSO: dimethyl sulfoxide
EGFR: epidermal growth factor receptor
equiv: equivalents
GSK3β: glycogen synthase kinase 3β
HATU: hexafluorophosphate azabenzotriazole tetramethyluronium
HEK293: human embryonic kidney 293
hERG: human ether-à-go-go-related gene
HPLC: high pressure liquid chromatography
LATS1/2: large tumor suppressor kinase 1 and 2
LC/MS: liquid chromatography/mass spectroscopy
MPLC: medium pressure liquid chromatography
MRT: mean residence time
MTS: 3-(4,5-dimethylthiazol-2-yl)-5-(3-carboxymethoxyphenyl)-2-(4-sulfophenyl)-2*H*-tetrazolium
NKD1: NKD inhibitor of WNT signaling pathway 1
NAD: nicotinamide adenine dinucleotide
NMR: nuclear magnetic resonance
PARP: poly-ADP-ribose polymerase
PARsylate: poly(ADP-ribose)sylate
PDA: photodiode array
PI3K: phosphoinositide-3-kinase
PK: pharmacokinetics
po: per oral
PPB: plasma protein binding
RT-qPCR: reverse transcription quantitative polymerase chain reaction
RNF146: ring finger protein 146
SD: standard deviation
SFC: supercritical fluid chromatography
*t*_1/2_: half-life
TAZ: tafazzin
TEAD: TEA domain transcription factor
TFA: trifluoroacetic acid
TLC: thin layer chromatography
TNKS: telomeric repeat factor (TRF1)-interacting ankyrin-related ADP-ribose polymerases, tankyrase
TNKS1/2: tankyrase 1 and tankyrase 2
tPSA: total polar surface area
Vd: volume of distribution
WNT: wingless-type mammary tumor virus integration site
YAP: yes associated protein 1
