# Supplementary material for: Preclinical Lead Optimization of a 1,2,4-Triazole Based Tankyrase Inhibitor
Source: J Med Chem. 2020 Jun 8;63(13):6834–46. doi: 10.1021/acs.jmedchem.0c00208 (PMC8008393; doi:10.1021/acs.jmedchem.0c00208)
Supplement: Supplementary file 1 — jm0c00208_si_001.pdf [file jm0c00208_si_001.pdf]

Supplementary Information

Pre-clinical lead optimization of a 1,2,4-triazole based tankyrase inhibitor

Jo Waaler,[*,†,‡,#] Ruben G. G. Leenders,[§,#] Sven T. Sowa,[|,#] Shoshy Alam Brinch,[†,‡] Max Lycke,[†,‡] Piotr Nieczypor,[§] Sjoerd Aertssen,[§] Sudarshan Murthy,[|] Albert Galera-Prat,[|] Eddy Damen,[§] Anita Wegert,[§] Marc Nazaré,[||] Lari Lehtiö,[|] and Stefan Krauss[†,‡]

[†]Hybrid Technology Hub - Centre of Excellence, Institute of Basic Medical Sciences, University of Oslo, P.O. Box 1110 Blindern, 0317, Oslo, Norway.
[‡]Department of Immunology and Transfusion Medicine, Oslo University Hospital, P.O. Box 4950 Nydalen, 0424, Oslo, Norway.
[§]Mercachem BV, Kerkenbos 1013, 6546 BB Nijmegen, the Netherlands.
[|]Faculty of Biochemistry and Molecular Medicine, Biocenter Oulu, University of Oulu, PO Box 5400, 90014 Oulu, Finland.
[||]Medicinal Chemistry, Leibniz-Forschungsinstitut für Molekulare Pharmakologie (FMP), Campus Berlin Buch, Robert-Roessle-Str. 10, 13125, Berlin, Germany.

Contents

Supplementary Table 1. East, West, South and Linker variations.

Supplementary Figure 1. Co-crystal structures of TNKS2 with inhibitors.

Supplementary Table 2. East-side variations of compound **13**.

Supplementary Table 3. Inhibition of ARTDs/PARPs.

Supplementary Figure 2. Exemplary dose-response measurements of **13** with TNKS1 and TNKS2.

Supplementary Figure 3. Quantification of immunoblots shown in Fig. 5a.

Supplementary Table 4. Data collection and refinement statistics for the crystal structures.

a)

East variation	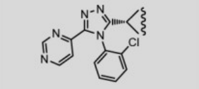	Biochemical IC_{50} TNKS2 (nM)	Cellular IC_{50} HEK293 (nM)
1		6.3	19
17		34	116
18		48	274
36		5.5	3
37		6.4	30
38		6.7	40
39		4.1	30
40		1700	7880
20		4.9	1.7
41		18	50
42		57	70
43		3.5	4
44		25000	>100000
45		2.7	2.4
46		37	57
47		3.0	6.7
48		5.4	1
21		2.6	0.048
49		350	750
50		680	1300
51		6.6	32
52		6300	>10000
53		1700	2260
54		2300	8900

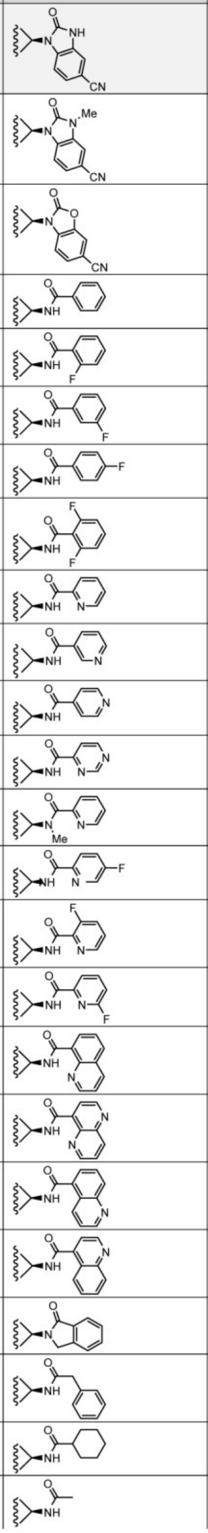

b)

West variation	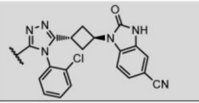	Biochemical IC_{50} TNKS2 (nM)	Cellular IC_{50} HEK293 (nM)
1		6.3	19
55		3.9	40
56		8.4	330
57		18	43
58		5.7	51
22		9.3	21
59		2.3	140
60		23	290
61		30	400
62		120	6400
63		220000	>10000
64		2600	8410

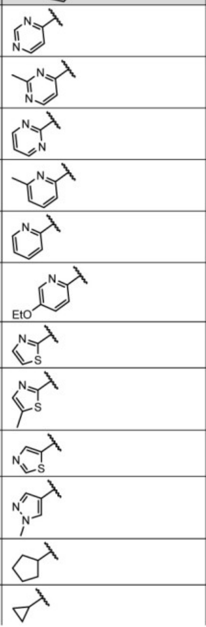

c)

South variation	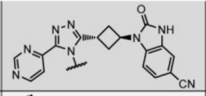	Biochemical IC_{50} TNKS2 (nM)	Cellular IC_{50} HEK293 (nM)
1		6.3	19
65		15	200
66		5.4	30
67		19	260
23		30	142
68		23	310
69		50	590
70		25	290
71		49	460
72		170	300
73		1700	1050
74		5600	>10000

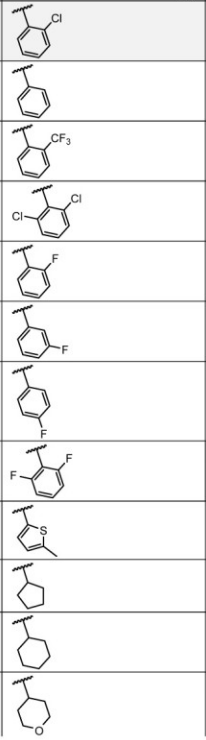

d)

Linker variation	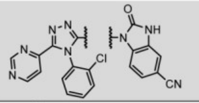	Biochemical IC_{50} TNKS2 (nM)	Cellular IC_{50} HEK293 (nM)
1		6.3	19
75		44	220
19		3.6	4.7

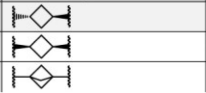

Supplementary Table 1. (a) East, (b) West, (c) South and Linker (d) variations.

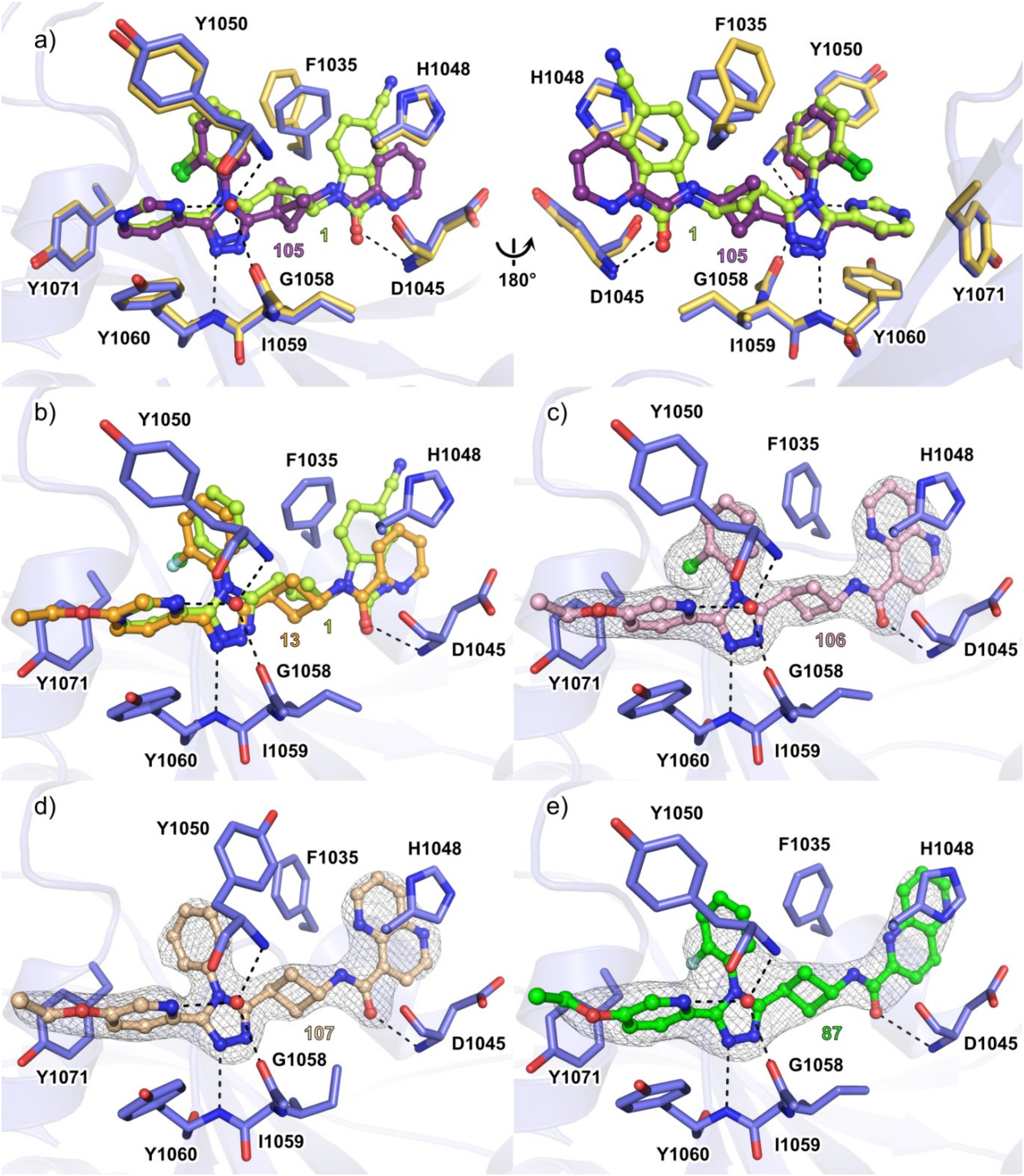

Supplementary Figure 1. Co-crystal structures of TNKS2 with inhibitors. (a) Superposition of **105** and **1** co-crystal structures (PDB codes 6TKN, 5NOB) showing the compounds and TNKS2 proteins for the TNKS2-**105** co-crystal structure (blue) and TNKS2- **1** co-crystal structure (yellow). Hydrogen bonds (dashed lines) and a water molecule (red sphere) are shown for the TNKS2-**105** co-crystal structure. (b) Superposition of **13** and **1** co-crystal structures showing the compounds and TNKS2 protein from the TNKS2- **13** co-crystal structure (PDB codes 6TG4, 5NOB). (c) Binding mode of **106** with TNKS2 catalytic domain (PDB code 6TKP). (d) Binding mode of **107** with TNKS2 catalytic domain (PDB code 6TKQ). (e) Binding mode of **87** with TNKS2 catalytic domain (PDB code 6TKR). The σ_A weighted $2F_o{-}F_c$ electron density maps around the ligands are contoured at 1.4-1.7σ. Crystal structures were solved with molecular replacement using the structure of TNKS2 (PDB code: 5NOB) as a starting model.

ID	East point mutations	Biochemical IC_{50} TNKS2 (nM)	Cellular IC_{50} HEK293 (nM)<br>**Solubility** (μM)
	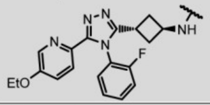		
**76**	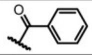	9.5	73
**77**	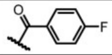	10	62
**13**	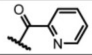	14	19<br>(>80)
**78**	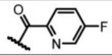	4.2	14
**79**	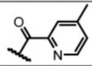	4.5	25
**80**	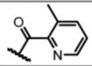	190	919
**81**	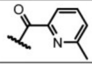	5.0	39
**82**	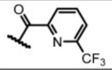	28	70
**83**	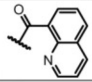	2.1	1.1<br>(3.4)
**10**	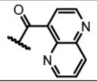	4.3	0.63<br>(>80)
**84**	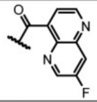	2	0.17<br>(3.1)
**85**	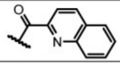	6.4	14<br>(14)
**86**	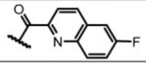	9.1	12
**87**	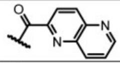	1.5	5.0<br>(<2)
**88**	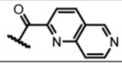	2.8	7.5<br>(>80)
**89**	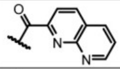	19	78<br>(<2)
**90**	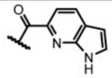	4.9	14<br>(<2)
**91**	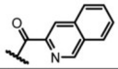	5.4	18<br>(13)
**92**	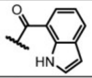	10	57
**93**	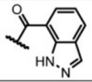	21	67
**94**	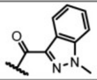	13	36
**95**	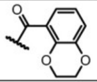	4.5	47
**96**	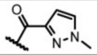	18	65
**97**	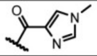	87	550
**98**	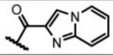	12	100
**99**	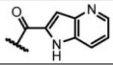	5.0	8.0
**100**	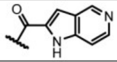	4.4	34
**101**	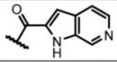	4.1	27
**102**	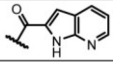	19	144
**103**	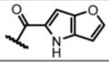	14	25
**104**	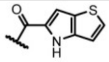	7.4	27

Supplementary Table 2. East-side variations of compound **13**.

	1**	13
**ARTD1/PARP1** (µM)	>100	29
**ARTD2/PARP2** (µM)	>100	26
**ARTD3/PARP3** (µM)	>100	75
**ARTD4/PARP4** (µM)	>100	>100
**ARTD5/TNKS1** (nM)	29 (7.54±0.007)	127 (6.90±0.05)
**ARTD6/TNKS2** (nM)	6.3 (8.20±0.03)	14 (7.85±0.04)
**ARTD7/PARP15**$^{\Delta}$(µM)	> 10	>>10
**ARTD8/PARP14** (µM)	> 10	>100
**ARTD10/PARP10** (µM)	> 10	>> 10
**ARTD12/PARP12**$^{\Delta}$(µM)	> 10	>>10

>> no inhibition detected
> below 50% inhibition
Δ concentration limited by DMSO tolerance
** *reference 11*

Supplementary Table 3. Inhibition of ARTDs/PARPs (IC_{50} [pIC_{50}±SEM]).

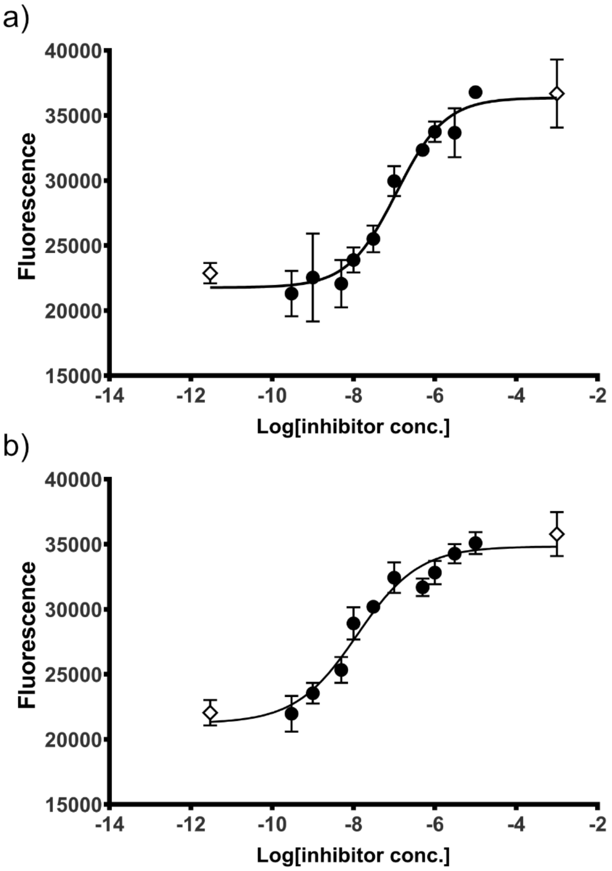

Supplementary Figure 2. Exemplary dose-response measurements of **13** with TNKS1 (a) and TNKS2 (b). The measurements were fitted using 4-parameters with GraphPad Prism. As compound showed no fluorescence interference, raw values were used for the fit. Controls were placed 2-logarithm units below or above the highest, and lowest compound concentrations (open diamonds).

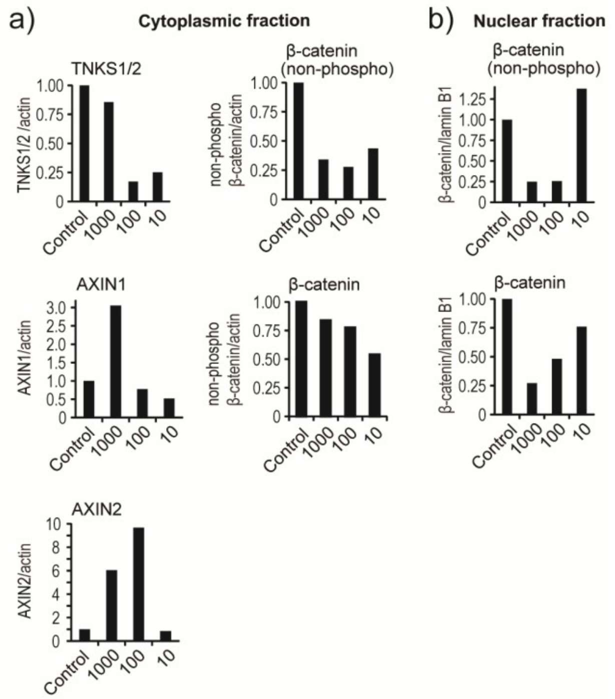

Supplementary Figure 3. Quantification of immunoblots (protein/actin or lamin B1 loading controls) relative to controls (0.01% DMSO = 1) shown in Fig. 5a. (a) Cytoplasmic TNKS1/2, AXIN1, AXIN2, transcriptionally active β-catenin (non-phospho) and β-catenin. (b) Nuclear active β-catenin and β-catenin.

a)

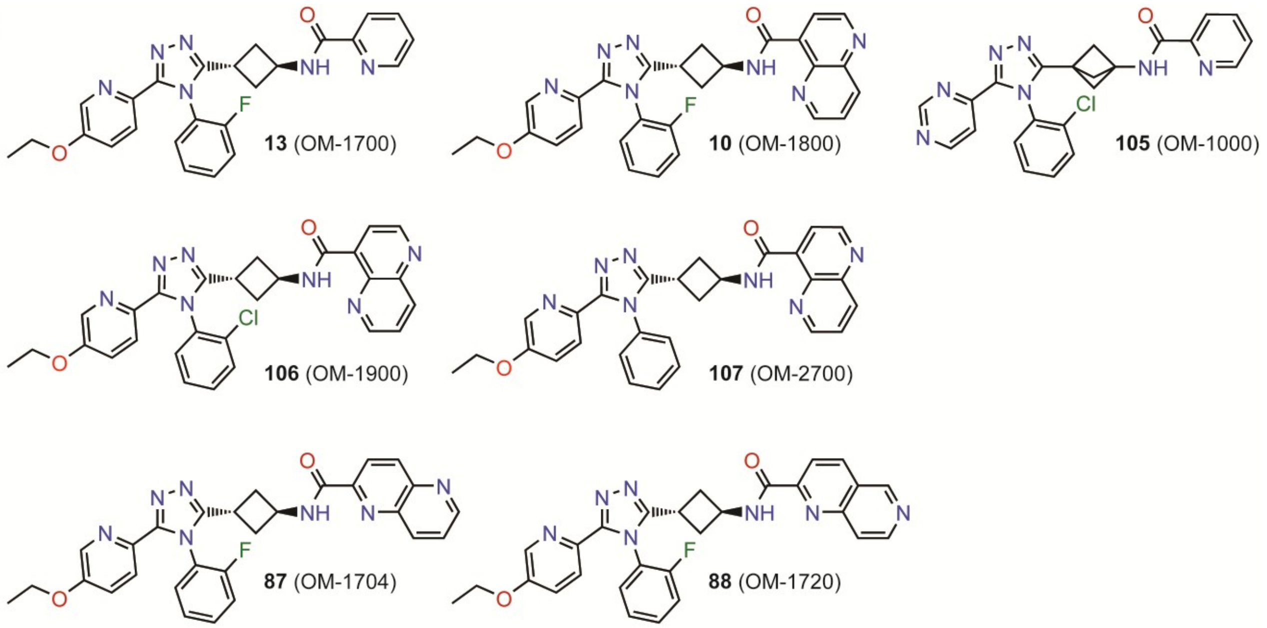

b)

**Compound**	**13**	**10**	**105**	**106**	**107**	**87**	**88**
**PDB code**	**6TG4**	**6TKM**	**6TKN**	**6TKP**	**6TKQ**	**6TKR**	**6TKS**
Beam line	ESRF ID30B	DLS I04	ESRF ID30B	ESRF ID23-1	ESRF ID23-1	DLS I04	ESRF ID30A-1
Wavelength (Å)	0.9677	0.9795	0.97625	1.03285	0.97625	0.9795	0.966
Space group	$P2_12_12_1$	$P2_12_12_1$	$P2_12_12_1$	$P2_12_12_1$	$P2_12_12_1$	$P2_12_12_1$	$P2_12_12_1$
Cell dimensions	42.68,	42.32,	41.73,	42.01,	42.12,	41.99,	41.60,
a, b, c (Å)	77.9,	77.41,	76.76,	77.08,	76.84,	76.76,	76.42,
	149.52	148.68	147.91	148.36	148.8	148.12	148.19
Resolution (Å)	37 - 2.76	29.7 - 2.7	38.4 - 2.5	41.6 - 2.4	41.7 - 2.5	41.5 - 2.75	42 - 2.50
	(2.86-2.76)	(2.80- 2.7)	(2.59- 2.5)	(2.49- 2.4)	(2.59- 2.5)	(2.85- 2.75)	(2.56-2.50)
R_{merge}	29.0 (153.7)	24.4 (114.4)	22.0 (151.7)	12.1 (87.4)	19.7 (85.1)	30.1 (200.1)	10.1 (72.5)
$I / \sigma I$	5.33 (1.06)	5.09 (1.21)	7.64 (1.10)	11.03 (1.93)	8.37 (2.04)	5.66 (1.12)	9.91 (2.03)
Completeness (%)	99.3 (99.6)	98.4 (99.9)	99.8 (99.6)	99.8 (99.9)	99.7 (99.5)	98.3 (99.5)	99.3 (98.7)
Redundancy	6.2 (5.8)	3.4 (3.5)	6.3 (6.5)	6.5 (6.3)	6.5 (6.7)	6.4 (6.5)	5.4 (5.7)
**Refinement**							
R_{work} / R_{free}	0.226/0.270	0.237/0.265	0.201/ 0.244	0.202/ 0.231	0.188/0.229	0.228/0.282	0.214/0.259
**B-factors**							
Protein	49	42	45	44	35	55	58
Inhibitor	42	31	38	36	24	41	51
**R.m.s.d.**							
Bond lengths (Å)	0.013	0.013	0.013	0.013	0.013	0.013	0.013
Bond angles (°)	1.72	1.64	1.72	1.64	1.79	1.72	1.66
**Ramachandran plot (%)**							
Favored regions	97.22	97.24	96.03	99.5	99	96.02	97.01
Additionally allowed regions	2.78	2.76	3.97	0.5	1	3.98	2.99

Supplementary Table 4. (a) Structures for compounds shown in (b). (b) Data collection and refinement statistics for the crystal structures.
