# Supplementary material for: Preclinical Lead Optimization of a 1,2,4-Triazole Based Tankyrase Inhibitor
Source: J Med Chem. 2020 Jun 8;63(13):6834–46. doi: 10.1021/acs.jmedchem.0c00208 (PMC8008393; doi:10.1021/acs.jmedchem.0c00208)

Supplementary Experimentals

Pre-clinical lead optimization of a 1,2,4-triazole based tankyrase inhibitor

Jo Waaler,[*,†,‡,#] Ruben G. G. Leenders,[§,#] Sven T. Sowa,[|,#] Shoshy Alam Brinch,[†,‡] Max Lycke,[†,‡] Piotr Nieczypor,[§] Sjoerd Aertssen,[§] Sudarshan Murthy,[|] Albert Galera-Prat,[|] Eddy Damen,[§] Anita Wegert,[§] Marc Nazaré,[||] Lari Lehtiö,[|] and Stefan Krauss[†,‡]

[†]Hybrid Technology Hub - Centre of Excellence, Institute of Basic Medical Sciences, University of Oslo, P.O. Box 1110 Blindern, 0317, Oslo, Norway.
[‡]Department of Immunology and Transfusion Medicine, Oslo University Hospital, P.O. Box 4950 Nydalen, 0424, Oslo, Norway.
[§]Mercachem BV, Kerkenbos 1013, 6546 BB Nijmegen, the Netherlands.
[|]Faculty of Biochemistry and Molecular Medicine, Biocenter Oulu, University of Oulu, PO Box 5400, 90014 Oulu, Finland.
[||]Medicinal Chemistry, Leibniz-Forschungsinstitut für Molekulare Pharmakologie (FMP), Campus Berlin Buch, Robert-Roessle-Str. 10, 13125, Berlin, Germany.

General synthetic procedures:

General Procedure A: amide preparation

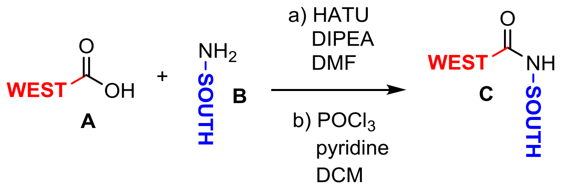

Method a): To a solution of an appropriate acid **A** (1.0 equiv.) and DIPEA (1.2 equiv.) in dried DMF (0.2–0.5 M) was added HATU (1.1 equiv.) under an inert atmosphere. The reaction was stirred for 1 hour before the suitable amine **B** (1.1 equiv.) was added. The stirring was continued for 2 to 24 hours and then evaporated to dryness. The residue was either first extracted (treated with a diluted aqueous sodium bicarbonate and DCM) or directly purified by flash column chromatography on silica gel (gradient of ethyl acetate in heptane, usually 10% to 100%) to afford the target amides **C**.

Method b): An equimolar mixture of the starting acid and amine were dissolved in a 5:1 mixture of DCM and pyridine (reaction molarity 0.2-0.5 M), the solution was cooled in an ice-bath and treated by a dropwise addition of 1.0-1.1 equiv. of phosphorous oxychloride. The cooling bath was removed and the mixture was stirred at ambient temperature for 1 to 18 hours. After an acidic extractive work-up, drying and chromatography, the desired amides were obtained.

General Procedure B: thioamide preparation

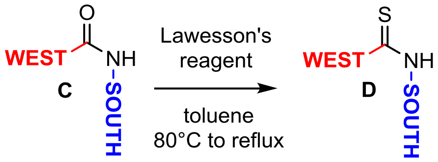

The amide **C** (1.0 equiv.) was suspended under a nitrogen atmosphere in anhydrous toluene (0.10 to 0.25 M). Lawesson's reagent (1 equiv.) was added and the mixture was heated at temperature between 80 °C and reflux during 2 to 24 hours. After the reaction mixture was concentrated, the residue was extracted with DCM from aqueous phase or directly purified by flash column chromatography on silica gel (usually a gradient of ethyl acetate in heptane was used, sometimes a gradient of DCM in heptane) to afford a batch of desired thioamide **D**.

General Procedure C: methylation of thioamide

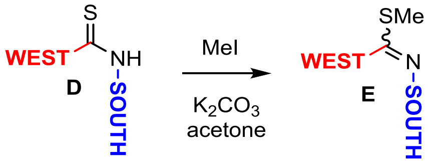

To a solution of thioamide **D** (1.0 equiv.) and iodomethane (1.1-1.3 equiv.) in acetone (0.10 to 0.30 M) was added potassium carbonate (1.3–1.5 equiv.). The suspension was stirred at room temperature until the reaction completion (from 2 hours up to overnight). After solvent evaporation, the reaction mixture was either extracted from aqueous solution with DCM affording the crude product **E** (as a mixture of E/Z isomers) that could be used without any purification in the next step. For better results in the next step, the crude product **E** might be flashed over silica gel column eluted with a gradient of ethyl acetate (5% to 30%) in heptane.

General Procedure D: triazole cyclisation

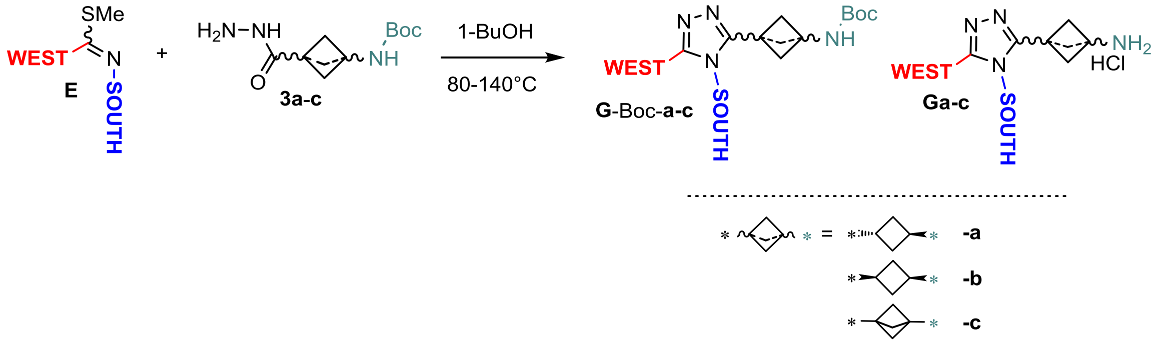

A suspension of carbimidothioate **E** (1.0-1.1 equiv.) and an appropriate hydrazide **3a-c** (1.0-1.1 equiv.) in 1-butanol (0.10 to 0.30 M) was placed into a microwave vial and closed with a cap. The mixture was irradiated (or heated in an oil bath) at a temperature ranging from 80 to 140 °C until the completion of the reaction (typically 5 to 20 hours). After evaporating to dryness, the residue was purified by flash column chromatography on silica gel (gradient of ethyl acetate in heptane as eluent) to afford 1,2,4-triazole derivatives Boc protected **G**-Boc-**a** to **G**-Boc-**c** or **Ga** to **Gc**, depending on the reaction temperature.

General Procedure E: Boc removal

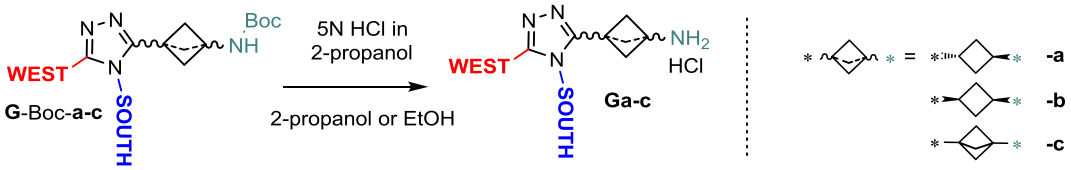

To a solution or suspension of the Boc-protected 1,2,4-triazole derivate **G**-Boc (1.0 equiv.) in absolute ethanol or 2-propanol (0.05 to 0.25 M) was added hydrogen chloride as a 5 N solution in 2-propanol (10-40 equiv.). The reaction was stirred at ambient or slightly elevated (50-60 °C) temperature during 2 to 18 hours. After reaction completion (if needed, extra portions of HCl solution were added) the solvents were removed *in vacuo*, sometimes stripping the residue with acetonitrile. The crude salt **G**, or a dihydrochloride depending on the actual South and West moieties. This was used as such in the final step.

General Procedure F: amide coupling towards the target molecules

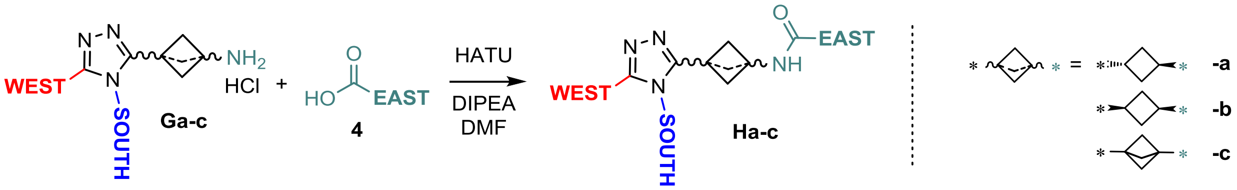

To a suspension or solution of the appropriate acid **4** (1.1 equiv.) and HATU (1.2 equiv.) in anhydrous acetonitrile or DMF (0.02 to 0.10 M) was added DIPEA (4.0 equiv.) and the mixture was stirred from 30 to 60 minutes, preferably under an inert atmosphere before amine **G** (1.0 equiv.) was added. The coupling was complete mostly within 1-3 hours, when the mixture was concentrated to dryness. The residue was submitted to purification by preparative SFC or by flash silica gel chromatography (0% via 3-5% to 10% gradient of methanol in DCM) followed by basic mode reversed-phase column (PoraPak Rxn RP, gradient acetonitrile in 10 mM aqueous ammonium bicarbonate). Final compounds **H** were obtained mostly as a white powder after lyophilisation from acetonitrile / water.

Preparation of tert-butyl (*trans*-3-(hydrazinecarbonyl)cyclobutyl)carbamate (3a)

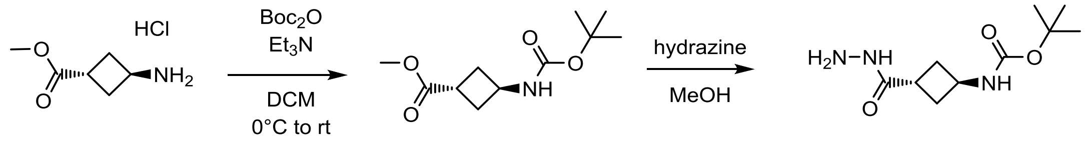

Step (a): Methyl *trans*-3-amino-cyclobutanecarboxylate hydrochloride (39.94 g, 241 mmol) was suspended in DCM (400 mL). The solution was cooled to 0 °C and triethylamine (4 equiv., 134 mL, 965 mmol) and Boc anhydride (1.2 equiv., 63.2 g, 289 mmol) were added while stirring. The mixture was allowing to warm up to room temperature and after 20 hours, the salts were filtered off and the filtrate was rinsed three times with water. The organic layer was dried over sodium sulphate, filtered and concentrated *in vacuo*. The residue was triturated in heptane, the solid was filtered off and air dried to give methyl *trans*-3-((tert-butoxycarbonyl)amino)cyclobutane-1-carboxylate as a white solid (46.3 gram, 80% yield). LC/MS (ESI) m/z for $C_{11}H_{19}NO_4$ = 229 (calculated), 215 ($[M-Me]^+$, found). ^1H NMR (400 MHz, chloroform-d) δ 4.73 (br s, 1H), 4.31 (br s, 1H), 3.70 (s, 3H), 3.01 (pseudo hept, J = 9.6, 1H), 2.62 (ddd, J = 12.9, 7.8, 3.7 Hz, 2H), 2.27 – 2.10 (m, 2H), 1.44 (s, 9H).

Step (b): Methyl *trans*-3-((tert-butoxycarbonyl)amino)cyclobutane-1-carboxylate (2.50 g, 8.72 mmol) was suspended in methanol (60 mL) and hydrazine hydrate (2.54 mL, 52.3 mmol) was added. The mixture was stirred at ambient temperature during 18 hours. The resulting suspension was filtered over a glass filter. The white solids were rinsed twice with water and twice with diethyl ether. The white solid was air dried to yield the title compound (**3a**) as a white solid (4.75 gram, 95% yield). LC/MS (ESI) m/z for $C_{10}H_{19}N_3O_3$ = 229 (calculated), 174 ($[M\text{-}t\text{-}Bu]^+$, found). ^1H NMR (400 MHz, DMSO-d6) δ 8.88 (br s, 1H), 7.13 (d, J = 8.1 Hz, 1H), 4.25 – 4.06 (m, 3H), 2.71 (tt, J = 9.0, 3.7 Hz, 1H), 2.32 – 2.17 (m, 2H), 2.12 – 1.97 (m, 2H), 1.36 (s, 9H).

Preparation of tert-butyl (*cis*-3-(hydrazinecarbonyl)cyclobutyl)carbamate (3b)

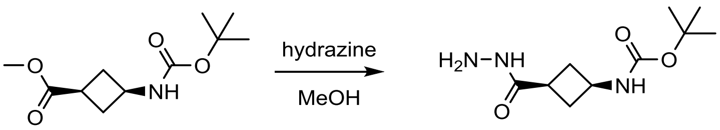

Methyl cis-3-((tert-butoxycarbonyl)amino)cyclobutane-1-carboxylate (0.115 g, 0.50 mmol) was dissolved in methanol (2.0 ml) and the solution was treated with an excess of hydrazine monohydrate (0.122 ml, 2.500 mmol). The colourless mixture was stirred for 65 hours at room temperature, then evaporated to dryness and stripped twice with acetonitrile providing 127 mg (~90% pure, quant.) of a white solid of the crude title product (**3b**). LC/MS (ESI) m/z for $C_{10}H_{19}N_3O_3$ = 229 (calculated), 174 ($[M\text{-}t\text{-}Bu]^+$, found).

Preparation of tert-butyl (3-(hydrazinecarbonyl)bicyclo[1.1.1]pentan-1-yl)carbamate (3c)

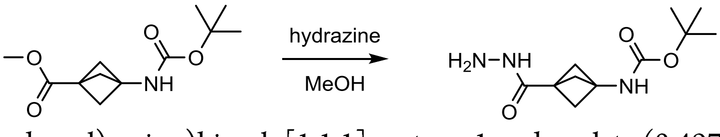

Methyl 3-((tert-butoxycarbonyl)amino)bicyclo[1.1.1]pentane-1-carboxylate (0.497 g, 2.00 mmol) was suspended in methanol (10 mL) and hydrazine hydrate (0.974 mL, 20.00 mmol) was added. The mixture was stirred at room temperature for 18 hours. After completion the mixture was evaporated to dryness, stripped twice with methanol and acetonitrile to give the title compound (**3c**) as an off-white solid (482 mg, 97% yield). LC/MS (ESI) m/z for $C_{11}H_{19}N_3O_3$ = 241 (calculated), 242 ($[M+H]^+$, found). ^1H NMR (400 MHz, DMSO-d6) δ 9.01 (s, 1H), 7.56 (br s, 1H), 4.18 (s, 2H), 2.02 (s, 6H), 1.37 (s, 9H).

Preparation of 3-(*trans*-3-(4-(2-chlorophenyl)-5-(pyrimidin-4-yl)-4H-1,2,4-triazol-3-yl)cyclobutyl)-2-oxo-2,3-dihydrobenzo[d]oxazole-6-carbonitrile (18)

Step (a): To 4-fluoro-3-hydroxybenzonitrile (**5**) (0.206 g, 1.5 mmol) and potassium carbonate (0.207 g, 1.500 mmol) under nitrogen, acetonitrile (anhydrous, 3 mL) was added followed by (bromomethyl)benzene (0.196 mL, 1.650 mmol) and the white suspension was heated at 60 °C for 3 hours. The suspension was evaporated to dryness, re-dissolved in a mixture of water and DCM and extracted three times with DCM. After drying over sodium sulphate, filtration and thorough evaporation, a batch of 0.33 g (~100% yield) of a white solid of 3-(benzyloxy)-4-fluorobenzonitrile (**6**) was isolated and employed as such in the follow up experiment. LC/MS (ESI) m/z for $C_{14}H_{10}FNO$ = 227 (calculated), 228 ($[M+H]^+$, found)

Step (b): A 0.5-2 mL microwave vial was charged 3-(benzyloxy)-4-fluorobenzonitrile (**6**) (0.040 g, 0.176 mmol) and potassium carbonate (0.055 g, 0.400 mmol) and *trans*-3-(4-(-2-chlorophenyl)-5-(pyrimidin-4-yl)-4H-1,2,4-triazol-3-yl)cyclobutan-1-amine hydrochloride (**Ga** HCl) (0.065 g, 0.16 mmol) in dimethyl sulfoxide (dry, 1.6 mL) was added. The suspension was then irradiated for 1 hour at 120 °C and at 150 °C for 3 hours in a microwave, then it was heated conventionally for 80 hours at 120 °C. The mixture was then diluted with water and extracted three times with DCM. After drying over sodium sulphate, the crude product was subjected to silica gel column chromatography eluting with a gradient of methanol (0 to 5%) in DCM, followed by a second column with a gradient of ethyl acetate (0 to 100%) in DCM. About 12 mg (13% yield) of a colourless glass of the intermediate 3-(benzyloxy)-4-((*trans*-3-(4-(2-chlorophenyl)-5-(pyrimidin-4-yl)-4H-1,2,4-triazol-3-yl)cyclobutyl)amino)benzonitrile (7) was obtained. LC/MS (ESI) m/z for $C_{30}H_{24}ClN_7O$ = 533 / 535 (calculated), 534 / 536 ($[M+H]^+$, found)

Step (c): Benzonitrile (7) (12 mg, 0.023 mmol) was dissolved in a mixture of ethanol (0.50 mL), ethyl acetate (0.50 mL) and DCM (0.50 mL). Palladium on carbon (4.9 mg, 4.60 µmol, 10%) was added and the mixture was stirred under 1 bar of hydrogen for 17 hours. Then an extra portion of palladium on carbon (4.9 mg, 4.60 µmol) was added and the hydrogenolysis was continued for six hours. The mixture was then filtered through celite rinsed with EtOH, DCM and ethyl acetate. The off-white residue of crude product (**8**) of 4-((*trans*-3-(4-(2-chlorophenyl)-5-(pyrimidin-4-yl)-4H-1,2,4-triazol-3-yl)cyclobutyl)amino)-3-hydroxybenzonitrile, (~10 mg, ~60% purity) was directly employed in the final stage. LC/MS (ESI) m/z for $C_{23}H_{18}ClN_7O$ = 443 / 445 (calculated), 444 / 446 ($[M+H]^+$, found)

Step (d): See the main manuscript for the final step.

Preparation of (1S,3r)-3-(4-(2-chlorophenyl)-5-(pyrimidin-4-yl)-4H-1,2,4-triazol-3-yl)cyclobutan-1-amine (Ga)

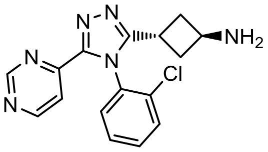

Step (a): *N*-(2-chlorophenyl)pyrimidine-4-carboxamide was prepared according to the General Procedure **A**, method a) as a purple solid (16.4 g, 87% yield). LC/MS (ESI) m/z for $C_{11}H_8ClN_3O$ = 233 / 235 (calculated) 234 / 236 ($[M+H]^+$, found). ^{1}H NMR (400 MHz, DMSO-d6) δ 10.59 (s, 1H), 9.46 (d, J = 1.5 Hz, 1H), 9.19 (d, J = 5.1 Hz, 1H), 8.26 (dd, J = 8.0, 1.6 Hz, 1H), 8.18 (dd, J = 5.1, 1.5 Hz, 1H), 7.60 (dd, J = 8.0, 1.4 Hz, 1H), 7.45 (td, J = 7.8, 1.5 Hz, 1H), 7.27 (td, J = 7.7, 1.6 Hz, 1H).

Step (b): *N*-(2-chlorophenyl)pyrimidine-4-carbothioamide was prepared following the General Procedure **B** and obtained as an orange solid (18.0 g, 99% yield). LC/MS (ESI) m/z for $C_{11}H_8ClN_3S$ = 249 / 251 (calculated) 250 / 252 ($[M+H]^+$, found). ^{1}H NMR (400 MHz, chloroform-d) δ 12.39 (s, 1H), 9.31 (s, 1H), 9.08 (dd, J = 8.2, 1.5 Hz, 1H), 9.01 (d, J = 5.3 Hz, 1H), 8.64 (dd, J = 5.1, 1.3 Hz, 1H), 7.54 (dd, J = 8.0, 1.5 Hz, 1H), 7.40 (td, J = 7.8, 1.5 Hz, 1H), 7.27 (td, J = 7.8, 1.6 Hz, 1H).

Step (c): methyl *N*-(2-chlorophenyl)pyrimidine-4-carbimidothioate (**E1**) was prepared according to the General Procedure **C** as an orange oil (13.2 g, ~90% pure, 89% yield). LC/MS (ESI) m/z for $C_{12}H_{10}ClN_3S$ = 263 / 265 (calculated) 264 / 266 ($[M+H]^+$, found). ^{1}H NMR (400 MHz, chloroform-*d*, broadened signals) δ 9.29 (s, 1H), 8.77 (br s, 1H), 7.38 (br d, J = 6.9 Hz, 1H), 7.15 (br s, 1H), 7.03 (br s, 2H), 6.78 (br s, 1H), 2.43 (br s, 3H).

Step (d): tert-butyl *trans*-3-(4-(2-chlorophenyl)-5-(pyrimidin-4-yl)-4H-1,2,4-triazol-3-yl)cyclobutyl)carbamate was prepared according to the General Procedure **D** as an off-white solid (961 mg, 90% pure, 86% yield). LC/MS (ESI) m/z for $C_{21}H_{23}ClN_6O_2$ = 426 / 428 (calculated) 427 / 429 ($[M+H]^+$, found). ^{1}H NMR (400 MHz, chloroform-d) δ 8.80 (pseudo d, J = 5.3 Hz, 2H), 8.26 (dd, J = 5.2, 1.3 Hz, 1H), 7.54 (dd, J = 7.9, 1.2 Hz, 1H), 7.49 (td, J = 7.3, 1.4 Hz, 1H), 7.42 (td, J = 7.6, 1.4 Hz, 1H), 7.27 (dd, J = 7.7, 1.5 Hz, 1H), 4.75 (br s, 1H), 4.35 (h, J = 6.8 Hz, 1H), 3.32 – 3.20 (m, 1H), 2.94 – 2.82 (m, 2H), 2.26 (br s, 2H), 1.42 (s, 9H).

Step (e): The title compound (**Ga**) was prepared similarly to the General Procedure **E**, but employing HCl in dioxane and methanol as main solvent. The free amine was liberated by re-dissolving the HCl salt in water and basification with aqueous potassium carbonate. After extraction with DCM, the organic layer was dried over sodium sulphate, filtered and evaporated to dryness giving the title compound as an orange oil (623 mg, 92% pure, 87% yield). This material was re-dissolved in acetonitrile and used as a 0.20 molar solution in the final step. LC/MS (ESI) m/z for $C_{16}H_{15}ClN_6$ = 326 / 328 (calculated) 327 / 328 ($[M+H]^+$, found). ^{1}H NMR (400 MHz, chloroform-d) δ 8.84 – 8.76 (m, 2H), 8.26 (dd, J = 5.3, 1.3 Hz, 1H), 7.53 (dd, J = 8.0, 1.6 Hz, 1H), 7.48 (td, J = 7.7, 1.6 Hz, 1H), 7.42 (td, J = 7.6, 1.6 Hz, 1H), 7.31 – 7.23 (m, 1H), 3.92 (p, J = 6.4 Hz, 1H), 3.27 (tt, J = 9.4, 5.0 Hz, 1H), 2.87 – 2.73 (m, 2H), 2.06 – 1.88 (m, 2H), 1.45 (br s, 2H).

Preparation of methyl *N*-(2-chlorophenyl)-5-ethoxypyridine-2-carbimidothioate (E2)

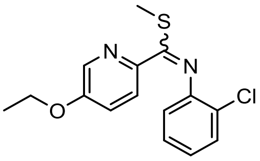

Step (a): *N*-(2-chlorophenyl)-5-ethoxypicolinamide was prepared according to the General Procedure **A**, method a) as an off-white solid (596 mg, 72% yield). LC/MS (ESI) m/z for $C_{14}H_{13}ClN_2O_2$ = 276 / 278 (calculated) 277 / 279 ($[M+H]^+$, found). ^{1}H NMR (400 MHz, chloroform-d) δ 10.52 (br s, 1H), 8.64 (dd, J = 8.3, 1.5 Hz, 1H), 8.30 (d, J = 2.8 Hz, 1H), 8.23 (d, J = 8.6 Hz, 1H), 7.42 (dd, J = 8.0, 1.5 Hz, 1H), 7.36 – 7.29 (m, 2H), 7.06 (td, J = 7.7, 1.5 Hz, 1H), 4.17 (q, J = 7.0 Hz, 2H), 1.49 (t, J = 7.0 Hz, 3H).

Step (b): *N*-(2-chlorophenyl)-5-ethoxypyridine-2-carbothioamide was prepared following the General Procedure **B** and obtained as a yellow solid (447 mg, 80% yield). LC/MS (ESI) not acquired. ^{1}H NMR (400 MHz, chloroform-d) δ 12.25 (br s, 1H), 9.00 (dd, J = 8.3, 1.5 Hz, 1H), 8.74 (d, J = 8.8 Hz, 1H), 8.23 (d, J = 2.8 Hz, 1H), 7.50 (dd, J = 8.1, 1.5 Hz, 1H), 7.37 (td, J = 7.8, 1.5 Hz, 1H), 7.30 (dd, J = 8.9, 2.9 Hz, 1H), 7.21 (td, J = 7.7, 1.6 Hz, 1H), 4.18 (q, J = 7.0 Hz, 2H), 1.49 (t, J = 7.0 Hz, 3H).

Step (c): the title compound (**E2**) was prepared according to the General Procedure **C** as a yellow oil solidifying upon standing (401 mg, 85% yield). LC/MS (ESI) m/z for $C_{15}H_{15}ClN_2OS$ = 306 / 308 (calculated) 307 / 309 ($[M+H]^+$, found). ^{1}H NMR (400 MHz, chloroform-d) δ 8.31 (d, J = 2.8 Hz, 1H), 7.37 (d, J = 7.9 Hz, 1H), 7.20 – 7.02 (br m, 2H), 6.98 (t, J = 8.0 Hz, 1H), 6.75 (br s, 1H), 4.08 (q, J = 7.0 Hz, 2H), 2.41 (br s, 3H), 1.44 (t, J = 6.9 Hz, 3H).

Preparation of methyl *N*-(2-fluorophenyl)pyrimidine-4-carbimidothioate (E3)

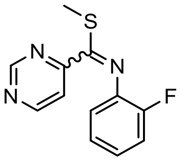

Step (a): *N*-(2-fluorophenyl)pyrimidine-4-carboxamide was prepared according to the General Procedure **A**, method a) from pyrimidine-4-carboxylic acid (500 mg, 4.03 mmol) and 2-fluoroaniline (0.428 mL, 4.43 mmol) as a white solid (381 mg, 43% yield). LC/MS (ESI) m/z for $C_{11}H_8FN_3O$ = 217 (calculated) 218 ($[M+H]^+$, found). ^{1}H NMR (400 MHz, chloroform-d) δ 10.20 (br s, 1H), 9.35 (d, J = 1.4 Hz, 1H), 9.06 (d, J = 5.0 Hz, 1H), 8.54 (td, J = 8.1, 1.7 Hz, 1H), 8.23 (dd, J = 5.0, 1.4 Hz, 1H), 7.25 – 7.11 (m, 3H).

Step (b): *N*-(2-fluorophenyl)pyrimidine-4-carbothioamide was prepared following the General Procedure **B** as an orange solid (317 mg, 77% yield). GC/MS (EI) m/z for $C_{11}H_8FN_3S$ = 233 (calculated) 233 ([M], found). 1H NMR (400 MHz, chloroform-d) δ 12.13 (br s, 1H), 9.29 (d, J = 1.4 Hz, 1H), 9.06 (td, J = 8.1, 2.3 Hz, 1H), 9.00 (d, J = 5.2 Hz, 1H), 8.63 (dd, J = 5.2, 1.4 Hz, 1H), 7.34 – 7.20 (m, 3H).

Step (c): The title compound (**E3**) was prepared according to the General Procedure **C** as a yellow oil (298 mg, 89% yield). LC/MS (ESI) m/z for $C_{12}H_{10}FN_3S$ = 247 (calculated) 248 ($[M+H]^+$, found). ^{1}H NMR (400 MHz, chloroform-d, broadened signals and partial integrals) δ 9.27 (br s, ~1H), 8.87 (br s, ~0.5H), 8.66 (br s, ~0.5H), 7.91 (br s, ~0.5H), 7.03 (br s, ~4H), 2.59 (br d, 3H).

Preparation of 1-(3-(4-(2-chlorophenyl)-5-(pyrimidin-4-yl)-4H-1,2,4-triazol-3-yl)bicyclo[1.1.1]pentan-1-yl)-2-oxo-2,3-dihydro-1H-benzo[d]imidazole-5-carbonitrile (19)

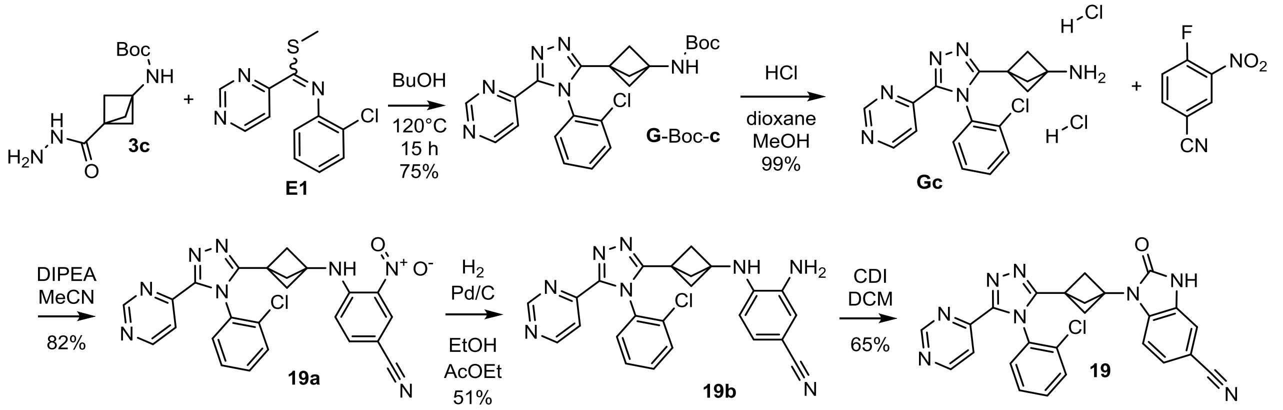

Step (a): tert-butyl (3-(4-(2-chlorophenyl)-5-(pyrimidin-4-yl)-4H-1,2,4-triazol-3-yl)bicyclo[1.1.1]pentan-1-yl)carbamate (**G**-Boc-**c**) was prepared from 124 mg (0.50 mmol) of tert-butyl (3-(hydrazinecarbonyl)bicyclo[1.1.1]pentan-1-yl)carbamate (**3c**) and 153 mg (0.55 mmol) of methyl *N*-(2-chlorophenyl)pyrimidine-4-carbimidothioate (**E1**) according to the General Procedure **D** as a yellow glass (167 mg, 75% yield). LC/MS (ESI) m/z for $C_{22}H_{23}ClN_6O_2$ = 438 / 440 (calculated) 439 / 441 ($[M+H]^+$ found). ^{1}H NMR (400 MHz, chloroform-d) δ 8.81 (d, J = 1.2 Hz, 1H), 8.79 (d, J = 5.3 Hz, 1H), 8.24 (dd, J = 5.2, 1.2 Hz, 1H), 7.58 – 7.47 (m, 2H), 7.43 (td, J = 7.5, 1.7 Hz, 1H), 7.34 (dd, J = 7.8, 1.2 Hz, 1H), 4.94 (s, 1H), 2.22 (br d, J = 3.9 Hz, 6H), 1.40 (s, 9H).

Step (b): crude 3-(4-(2-chlorophenyl)-5-(pyrimidin-4-yl)-4H-1,2,4-triazol-3-yl)bicyclo[1.1.1]pentan-1-amine dihydrochloride (**Gc**) was prepared according to the General Procedure **E** as a yellow glass (153 mg, 99% yield) of a presumed di-HCl salt based on mass balance. LC/MS (ESI) m/z for $C_{17}H_{15}ClN_6$ = 338 / 340 (calculated) 339 / 341 ($[M+H]^+$ found)

Step (c): A mixture of crude amine dihydrochloride (**Gc**) (0.153 g, 0.38 mmol) and 4-fluoro-3-nitrobenzonitrile (0.063 g, 0.380 mmol) was suspended in acetonitrile (3.8 mL). DIPEA (0.199 mL, 1.140 mmol) was added and the resulting solution was stirred overnight at ambient temperature. After evaporation of the solvent, the yellow residue was purified by silica flash chromatography (gradient to 5% methanol in DCM) resulting in 160 mg (95% pure, 82% yield) of a yellow foam of 4-((3-(4-(2-chlorophenyl)-5-(pyrimidin-4-yl)-4H-1,2,4-triazol-3-yl)bicyclo[1.1.1]pentan-1-yl)amino)-3-nitrobenzonitrile (**19a**). LC/MS (ESI) m/z for $C_{24}H_{17}ClN_8O_2$ = 484 / 486 (calculated) 485 / 487 ($[M+H]^+$ found). ^{1}H NMR (400 MHz, chloroform-d) δ 8.84 (s, 1H), 8.83 (d, J = 5.4 Hz, 1H), 8.60 (s, 1H), 8.49 (d, J = 1.9 Hz, 1H), 8.28 (dd, J = 5.2, 1.2 Hz, 1H), 7.65 – 7.53 (m, 3H), 7.49 (td, J = 7.5, 1.8 Hz, 1H), 7.40 (dd, J = 7.6, 1.3 Hz, 1H), 7.11 (d, J = 9.0 Hz, 1H), 2.51 – 2.39 (m - pseudo q, 6H).

Step (d): In a 50 mL flask nitrobenzonitrile (**19a**) (0.158 g, 0.31 mmol) was suspended in a mixture of ethyl acetate (6.0 mL) and ethanol (3.0 mL), 10% palladium on carbon (33 mg) was added and the mixture was placed under 1 bar of hydrogen atmosphere (a balloon). After 20 hours an additional portion of 10% palladium on carbon (33 mg) was added, stirring was continued for 3 hours. The mixture was filtered through a pad of celite rinsed with ethanol and ethyl acetate. The crude material was purified on silica gel eluting with a gradient of methanol (0% to 5%) in DCM. Two fractions of 3-amino-4-((3-(4-(2-chlorophenyl)-5-(pyrimidin-4-yl)-4H-1,2,4-triazol-3-yl)bicyclo[1.1.1]pentan-1-yl)amino)benzonitrile (**19b**) were collected: 36 mg of a pink glass of (~75% pure on LC/MS) and 82 mg of a red glass (~55% pure on LC/MS), 51% combined yield. LC/MS (ESI) m/z for $C_{24}H_{19}ClN_8$ = 454 / 456 (calculated) 455 / 457 ($[M+H]^+$ found).

Step (e): See the main manuscript for the final step.

Preparation of (1S,3r)-3-(4-(2-fluorophenyl)-5-(5-(methylsulfonyl)pyridin-2-yl)-4H-1,2,4-triazol-3-yl)cyclobutan-1-amine dihydrochloride (Ga-2)

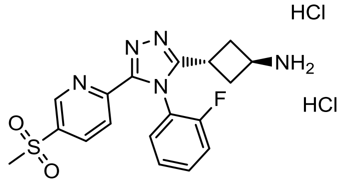

Step (a): *N*-(2-fluorophenyl)-5-(methylsulfonyl)picolinamide was prepared following the General Procedure **A**, method b) as a white solid (347 mg, 55% yield).
LC/MS (ESI) m/z for $C_{13}H_{11}FN_2O_3S$ = 294 (calculated) 295 ($[M+H]^+$, found).

Step (b): *N*-(2-fluorophenyl)-5-(methylsulfonyl)pyridine-2-carbothioamide was prepared according to the General Procedure **B** as an orange solid (270 mg, 78% yield). LC/MS (ESI) m/z for $C_{13}H_{11}FN_2O_2S_2$ = 310 (calculated) 311 ($[M+H]^+$, found). ^{1}H NMR (400 MHz, chloroform-d) δ 12.10 (br s, 1H), 9.13 (dd, J = 2.2, 0.8 Hz, 1H), 9.02 (td, J = 8.1, 2.1 Hz, 1H), 8.97 (dd, J = 8.4, 0.9 Hz, 1H), 8.41 (dd, J = 8.4, 2.3 Hz, 1H), 7.34 – 7.20 (m, 3H), 3.16 (s, 3H).

Step (c): methyl *N*-(2-fluorophenyl)-5-(methylsulfonyl)pyridine-2-carbimidothioate was prepared according to the General Procedure **C** as a yellow glassy solid (282 mg, 98% yield). LC/MS (ESI) m/z for $C_{14}H_{13}FN_2O_2S_2$ = 324 (calculated) 325 ($[M+H]^+$, found). ^{1}H NMR (400 MHz, chloroform-d) δ 9.15 (br s, 1H), 8.17 (very br s, 2H), 7.04 (very br s, 4H), 3.12 (s, 3H), 2.44 (very br d, 3H).

Step (d): tert-butyl ((1S,3r)-3-(4-(2-fluorophenyl)-5-(5-(methylsulfonyl)pyridin-2-yl)-4H-1,2,4-triazol-3-yl)cyclobutyl)carbamate was prepared according to the General Procedure **D** as a white solid (343 mg, 81% yield). LC/MS (ESI) m/z for $C_{23}H_{26}FN_5O_4S$ = 487 (calculated) 488 ($[M+H]^+$, found). ^{1}H NMR (400 MHz, chloroform-d) δ 8.71 (d, J = 2.2 Hz, 1H), 8.53 (dd, J = 8.4, 0.8 Hz, 1H), 8.27 (dd, J = 8.4, 2.4 Hz, 1H), 7.56 – 7.47 (m, 1H), 7.29 – 7.14 (m, 3H), 4.73 (br s, 1H), 4.35 (h, J = 7.1 Hz, 1H), 3.38 – 3.26 (m, 1H), 3.07 (s, 3H), 2.97 – 2.87 (m, 1H), 2.83 (br s, 1H), 2.29 (apparent br d, J = 30.5 Hz, 2H), 1.42 (s, 9H).

Step (e): the crude title compound (**Ga-2**) was obtained according to the General Procedure **E** as a white solid (319 mg, 100% yield). LC/MS (ESI) m/z for $C_{18}H_{18}FN_5O_2S$ = 387 (calculated) 388 ($[M+H]^+$, found). ^{1}H NMR (400 MHz, DMSO-d6) δ 8.71 (s, 1H), 8.44 (d, J = 2.3 Hz, 2H), 8.32 (br s, 3H, NH_2 + HCl), 7.68 – 7.61 (m, 1H), 7.59 (td, J = 7.8, 1.5 Hz, 1H), 7.49 (dd, J = 9.9, 8.5 Hz, 1H), 7.38 (t, J = 7.6 Hz, 1H), 3.87 (apparent h, J = 6.3 Hz, 1H), 3.56 (apparent tt, J = 9.9, 5.8 Hz, 1H), 3.33 (s, 3H), 2.86 – 2.75 (m, 1H), 2.66 – 2.55 (m, 1H), 2.45 – 2.28 (m, 2H).

Preparation of (1S,3r)-3-(5-(5-ethoxypyridin-2-yl)-4-(2-fluorophenyl)-4H-1,2,4-triazol-3-yl)cyclobutan-1-amine dihydrochloride (Ga-3)

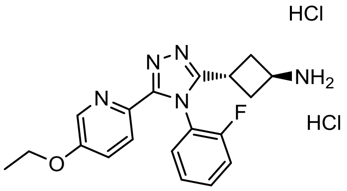

Step (a): 5-ethoxy-N-(2-fluorophenyl)picolinamide was prepared according to the General Procedure **A**, method a) as an off-white solid (747 mg, 80% yield). LC/MS (ESI) m/z for $C_{14}H_{13}FN_2O_2$ 260 (calcd) 261 ($[M+H]^+$, found). ^1H NMR (400 MHz, chloroform-d) δ 10.15 (s, 1H), 8.57 (td, J = 8.1, 1.6 Hz, 1H), 8.28 (d, J = 2.8 Hz, 1H), 8.22 (d, J = 8.7 Hz, 1H), 7.32 (dd, J = 8.7, 2.8 Hz, 1H), 7.23 – 7.08 (m, 2H), 7.10 – 7.03 (m, 1H), 4.17 (q, J = 7.0 Hz, 2H), 1.49 (t, J = 7.0 Hz, 3H).

Step (b): 5-ethoxy-N-(2-fluorophenyl)pyridine-2-carbothioamide was prepared following the General Procedure **B** as a yellow solid (546 mg, 100% yield). LC/MS (ESI) m/z for $C_{14}H_{13}FN_2OS$ 276 (calcd) 277 ($[M+H]^+$, found). ^1H NMR (400 MHz, chloroform-d) δ 12.02 (s, 1H), 9.04 – 8.97 (m, 1H), 8.73 (d, J = 8.8 Hz, 1H), 8.21 (d, J = 2.8 Hz, 1H), 7.30 (dd, J = 8.9, 2.9 Hz, 1H), 7.25 – 7.17 (m, 3H), 4.17 (q, J = 6.9 Hz, 2H), 1.49 (t, J = 7.0 Hz, 3H).

Step (c): methyl 5-ethoxy-N-(2-fluorophenyl)pyridine-2-carbimidothioate was prepared according to the to the General Procedure **C** as a yellow oil (222 mg, 91% yield). LC/MS (ESI) m/z for $C_{15}H_{15}FN_2OS$ 290 (calcd) 291 ($[M+H]^+$, found). ^1H NMR (400 MHz, chloroform-d) δ 8.30 (s, 1H), 7.20 – 6.63 (br m, 6H), 4.08 (br s, 2H), 2.49 (very br s, 3H), 1.43 (t, J = 6.8 Hz, 3H).

Step (d): tert-butyl ((1S,3r)-3-(5-(5-ethoxypyridin-2-yl)-4-(2-fluorophenyl)-4H-1,2,4-triazol-3-yl)cyclobutyl)carbamate was prepared according to the General Procedure **D** as a yellow foam (258 mg, 73% yield). LC/MS (ESI) m/z for $C_{24}H_{28}FN_5O_3$ 453 (calcd) 454 ($[M+H]^+$, found).

Step (e): the title compound (**Ga-3**) was prepared crude according to the General Procedure **E** as a purple glass (121 mg, 100% yield). LC/MS (ESI) m/z for $C_{19}H_{20}FN_5O{\cdot}353$ (calcd) 354 ($[M+H]^+$, found).

Preparation of (1S,3r)-3-(4-(2-fluorophenyl)-5-(pyridin-2-yl)-4H-1,2,4-triazol-3-yl)cyclobutan-1-amine dihydrochloride (Ga-4)

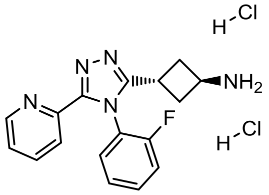

Step (a): N-(2-fluorophenyl)picolinamide was prepared according to the General Procedure **A**, method a) as an off-white solid (825 mg, 76% yield). LC/MS (ESI) m/z for $C_{12}H_9FN_2O$ 216 (calcd) 217 ($[M+H]^+$, found).

Step (b): N-(2-fluorophenyl)pyridine-2-carbothioamide was prepared according to the General Procedure **B** as an orange solid (792 mg, 89% yield). LC/MS (ESI) m/z for $C_{12}H_9FN_2S$ 232 (calcd) 233 ($[M+H]^+$, found).

Step (c): methyl N-(2-fluorophenyl)pyridine-2-carbimidothioate was prepared according to the General Procedure **C** as a yellow oil (809 mg, 97% yield). LC/MS (ESI) m/z for $C_{13}H_{11}FN_2S$: 246 (calcd) 247 ($[M+H]^+$, found).

Step (d): tert-butyl ((1S,3r)-3-(4-(2-fluorophenyl)-5-(pyridin-2-yl)-4H-1,2,4-triazol-3-yl)cyclobutyl)carbamate was prepared according to the General Procedure **D** as a light brown foam (653 mg, 77% yield). LC/MS (ESI) m/z for $C_{22}H_{24}FN_5O_2$ 409 (calcd) 410 ($[M+H]^+$, found). ^{1}H NMR (400 MHz, CDCl3) δ 8.23 (dt, J = 8.1, 1.1 Hz, 1H), 8.20 (dt, J = 4.8, 1.4 Hz, 1H), 7.74 (td, J = 7.8, 1.8 Hz, 1H), 7.46 (tdd, J = 7.9, 5.0, 2.2 Hz, 1H), 7.25 – 7.15 (m, 4H), 4.73 (br s, 1H), 4.40 – 4.26 (sym. m, 1H), 3.38 – 3.26 (m, 1H), 2.96 – 2.76 (m, 2H), 2.42 – 2.08 (m, 2H), 1.42 (s, 9H).

Step (e): the title amine salt (**Ga-4**) was prepared following the General Procedure **E** and obtained as a purple glass (661 mg, ~90% purity, ~100% yield). LC/MS (ESI) m/z for $C_{17}H_{16}FN_5$ 309 (calcd) 310 ($[M+H]^+$, found). ^{1}H NMR (400 MHz, DMSO-d6) δ 8.36 (br s, 3H, NH2+HCl), 8.26 (d, J = 4.6 Hz, 1H), 8.12 (d, J = 7.9 Hz, 1H), 7.94 (td, J = 7.7, 1.8 Hz, 1H), 7.64 – 7.50 (m, 2H), 7.45 (ddd, J = 9.8, 8.3, 1.4 Hz, 1H), 7.41 – 7.30 (m, 2H), 3.91 – 3.78 (m, 1H), 3.54 (tt, J = 9.5, 5.4 Hz, 1H), 2.78 (dt, J = 12.2, 6.9 Hz, 1H), 2.64 – 2.53 (m, 1H), 2.44 – 2.26 (m, 2H).

Preparation of (1S,3r)-3-(4-(2-fluorophenyl)-5-(thiazol-2-yl)-4H-1,2,4-triazol-3-yl)cyclobutan-1-amine dihydrochloride (Ga-5)

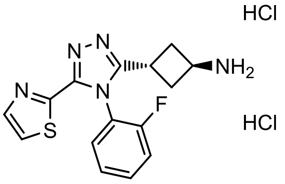

Step (a): N-(2-fluorophenyl)thiazole-2-carboxamide was prepared according to the General Procedure **A**, method b) as an off-white solid (1.80 g, 79% yield). LC/MS (ESI) m/z for $C_{10}H_7FN_2OS$ 222 (calcd) 223 ($[M+H]^+$, found).

Step (b): N-(2-fluorophenyl)thiazole-2-carbothioamide was prepared according to the General Procedure **B** as a yellow solid (1.63 g, 86% yield). LC/MS (ESI) m/z for $C_{10}H_7FN_2S_2$ 238 (calcd) 239 ($[M+H]^+$, found).

Step (c): methyl N-(2-fluorophenyl)thiazole-2-carbimidothioate was prepared according to the General Procedure **C** as a yellow oil (1.65 g, 95% pure, 91% yield) solidifying upon standing. LC/MS (ESI) m/z for $C_{11}H_9FN_2S_2$: 252 (calcd) 253 ($[M+H]^+$, found). ^{1}H NMR (400 MHz, chloroform-d, all signals very broad) δ 7.91 (pseudo d, J = 32.8 Hz, 1H), 7.47 (pseudo d, J = 40.9 Hz, 1H), 7.17 – 7.06 (m, 3H), 6.95 (pseudo d, J = 55.5 Hz, 1H), 2.52 (pseudo d, J = 36.5 Hz, 3H).

Step (d): tert-butyl ((1S,3r)-3-(4-(2-fluorophenyl)-5-(thiazol-2-yl)-4H-1,2,4-triazol-3-yl)cyclobutyl)carbamate was prepared according to the General Procedure **D** as a white solid (102 mg, 48% yield). LC/MS (ESI) m/z for $C_{20}H_{22}FN_5O_2S$ 415 (calcd) 416 ($[M+H]^+$, found). ^{1}H NMR (400 MHz, chloroform-d) δ 7.63 (d, J = 3.2 Hz, 1H), 7.54 (tdd, J = 7.5, 5.0, 2.0 Hz, 1H), 7.36 (d, J = 3.2 Hz, 1H), 7.33 – 7.21 (m, 3H), 4.75 (br s, 1H), 4.40 – 4.11 (m, 1H), 3.32 (br s, 1H), 2.95 – 2.85 (m, 1H), 2.83 (br s, 1H), 2.28 (br s, 2H), 1.42 (s, 9H).

Step (e): the title salt (**Ga-5**) was prepared following the General Procedure **E** and obtained as an off-white solid (97 mg, 100% yield). LC/MS (ESI) m/z for $C_{15}H_{14}FN_5S$ 315 (calcd) 316 ($[M+H]^+$, found).

3-(*trans*-3-(5-(5-ethoxypyridin-2-yl)-4-(2-fluorophenyl)-4H-1,2,4-triazol-3-yl)cyclobutyl)-1,5-naphthyridine-2-carboxamide (87). The title compound was prepared according to the General Procedure **F** as a white solid (17.0 mg, 66% yield). LC/MS (ESI) m/z for $C_{28}H_{24}FN_7O_2$ = 509 (calculated), 510 ($[M+H]^+$, found). 1H NMR (400 MHz, Chloroform-d) δ 9.06 (dd, J = 4.2, 1.7 Hz, 1H), 8.57 - 8.48 (m, 2H), 8.41 (dd, J = 8.4,1.7 Hz, 1H), 8.35 (d, J = 6.9 Hz, 1H), 8.17 (d, J = 8.7 Hz, 1H), 7.89 (d, J = 2.9 Hz, 1H), 7.71 (dd, J = 8.6, 4.2 Hz, 1H), 7.49 - 7.41 (m, 1H), 7.25 - 7.15 (m, 4H), 4.84 (h, J = 7.1 Hz, 1H), 4.04 (q, J = 7.0 Hz, 2H), 3.51 (tt, J = 9.8, 5.1 Hz, 1H), 3.14 - 3.01 (m, 2H), 2.62 - 2.46 (m, 2H), 1.40 (t, J = 7.0 Hz, 3H).

3-(*trans*-3-(5-(5-ethoxypyridin-2-yl)-4-(2-fluorophenyl)-4H-1,2,4-triazol-3-yl)cyclobutyl)-1,6-naphthyridine-2-carboxamide (88). The title compound was prepared according to the General Procedure **F** as a white solid (12.7 mg, 49% yield). LC/MS (ESI) m/z for $C_{28}H_{24}FN_7O_2$ 509 (calculated), 510 ($[M+H]^+$, found). 1H NMR (400 MHz, Chloroform-d) δ 9.36 (s, 1H), 8.82 (d, J = 5.9 Hz, 1H), 8.49 - 8.40 (m, 2H), 8.38 (d, J = 6.8 Hz, 1H), 8.17 (d, J = 8.7 Hz, 1H), 7.92 (d, J = 5.9 Hz, 1H), 7.89 (s, 1H), 7.50 - 7.40 (m, 1H), 7.25 - 7.15 (m, 4H), 4.86 (h, J = 7.3 Hz, 1H), 4.04 (q, J = 6.9 Hz, 2H), 3.50 (tt, J = 9.7, 5.2 Hz, 1H), 3.17 - 3.00 (m, 2H), 2.62 - 2.46 (m, 2H), 1.40 (t, J = 6.9 Hz, 3H).

1-(3-(4-(2-chlorophenyl)-5-(pyrimidin-4-yl)-4H-1,2,4-triazol-3-yl)bicyclo[1.1.1]pentan-1-yl)picolinamide (105). The title compound was prepared according to the General Procedure **F** as a white solid (15.2 mg, 67% yield). LC/MS (ESI) m/z for $C_{23}H_{18}ClN_7O$ 443 / 445 (calculated) 444 / 446 ($[M+H]^+$, found). 1H NMR (400 MHz, Chloroform-d) δ 8.83 (d, J = 1.3 Hz, 1H), 8.80 (d, J = 5.3 Hz, 1H), 8.51 (dq, J = 4.9, 0.8 Hz, 1H), 8.41 (br s, 1H), 8.26 (dd, J = 5.3, 1.3 Hz, 1H), 8.11 (d, J = 7.8 Hz, 1H), 7.84 (td, J = 7.7, 1.7 Hz, 1H), 7.56 (dd, J = 8.0, 1.6 Hz, 1H), 7.52 (td, J = 8.1, 7.6, 1.6 Hz, 1H), 7.48 - 7.40 (m, 2H), 7.38 (dd, J = 7.8, 1.4 Hz, 1H), 2.48 - 2.37 (m, 6H).

3-(*trans*-3-(4-(2-chlorophenyl)-5-(5-ethoxypyridin-2-yl)-4H-1,2,4-triazol-3-yl)cyclobutyl)-1,5-naphthyridine-4-carboxamide (106). The title compound was prepared according to the General Procedure **F** as a white solid (11.9 mg, 29% yield). LC/MS (ESI) m/z for $C_{28}H_{24}ClN_7O_2$ 525 / 527 (calculated) 526 / 528 ($[M+H]^+$, found). 1H NMR (400 MHz, Chloroform-d) δ 11.31 (d, J = 5.9 Hz, 1H), 9.14 (d, J = 4.5 Hz, IH), 8.98 (dd, J = 4.2, 1.8 Hz, 1H), 8.58 - 8.53 (m, 2H), 8.17 (d, J = 8.8 Hz, 1H), 7.86 (d, J = 2.8 Hz, 1H), 7.74 (dd, J = 8.5, 4.2 Hz, 1H), 7.48 (dd, J = 7.6, 1.9 Hz, 1H), 7.39 (dtd, J = 14.9, 7.3, 1.8 Hz, 2H), 7.32 (dd, J = 7.5, 2.0 Hz, 1H), 7.22 (dd, J = 8.8, 2.9 Hz, 1H), 4.84 (h, J = 7.2 Hz, 1H), 4.04 (q, J = 7.0 Hz, 2H), 3.47 (apparent tt, J = 9.5, 5.5 Hz, 1H), 3.18 - 2.99 (sym. m, 2H), 2.58 - 2.45 (sym. m, 2H), 1.40 (t, J = 7.0 Hz, 3H).

3-(*trans*-3-(5-(5-ethoxypyridin-2-yl)-4-phenyl-4H-1,2,4-triazol-3-yl)cyclobutyl)-1,5-naphthyridine-4-carboxamide (107). The title compound was prepared according to the General Procedure **F** as a white solid (19.9 mg, 31% yield). LC/MS (ESI) m/z for $C_{28}H_{25}N_7O_2$ 491 (calculated) 492 ($[M+H]^+$, found). 1H NMR (400 MHz, Chloroform-d) δ 11.31 (d, J = 6.0 Hz, 1H), 9.14 (d, J = 4.3 Hz, 1H), 8.97 (dd, J = 4.2, 1.9 Hz, 1H), 8.58 - 8.52 (m, 2H), 8.02 (d, J = 8.7 Hz, 1H), 7.94 (d, J = 2.8 Hz, 1H), 7.74 (dd, J = 8.6, 4.2 Hz, 1H), 7.43 (t, J = 3.1 Hz, 3H), 7.21 (dd, J = 8.7, 2.9 Hz, 1H), 7.19 - 7.15 (m, 2H), 4.85 (h, J = 7.2 Hz, 1H), 4.04 (q, J = 6.9 Hz, 2H), 3.56 (tt, J = 9.8, 5.4 Hz, 1H), 3.08 (ddd, J = 13.2, 8.2, 5.5 Hz, 2H), 2.54 (ddd, J = 12.7, 9.4, 6.1 Hz, 2H), 1.40 (t, J = 7.0 Hz, 3H).

HRMS, NMR and LC/MS spectra

Compound 13

title	**PINI21-96-2**
Method	AN_ACID.M
Date acquired	13-Mar-18, 17:58:01
FileName	Analysis\LCMS6_0313_137.D
Column	XSelect CSH C18 (50x2.1mm, 3.5μ) valve: 3
Flow	0.8 ml/min; Column temp: 35°C
Eluent A	0.1% Formic acid in acetonitrile
Eluent B	0.1% Formic acid in water
Gradient	t=0 min 5% A, t=3.5 min 98% A, t=6 min 98% A
Posttime	2 min
Detection	DAD (210, 220, 220-320nm)
Detection	PDA (210-320nm)
Detection	MSD (ESI pos/neg) mass range: 100-1000
Detection	ELSD gas temp: 40°C, flow 1.5 ml/min gain 1

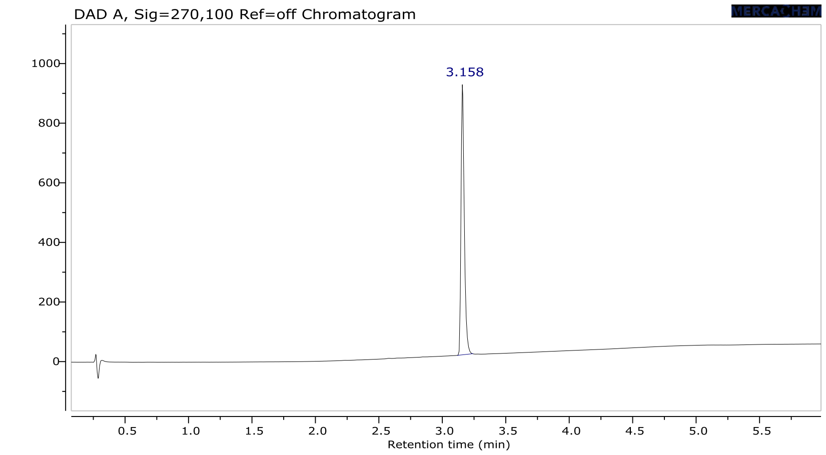

Integrals spectrum Chromatogram DAD A, Sig=270,100 Ref=off

rt (min)	height	area	area (%)
3.16	906.8	27.03	100.00

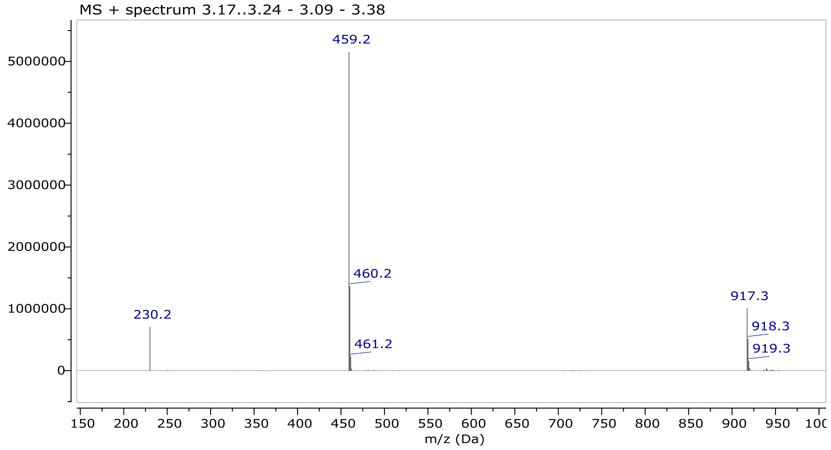

HRMS (m/z): C25H23FN6O2, [M+H]+ Calc: 459.19393; found: 459.19353, Δppm -0.87

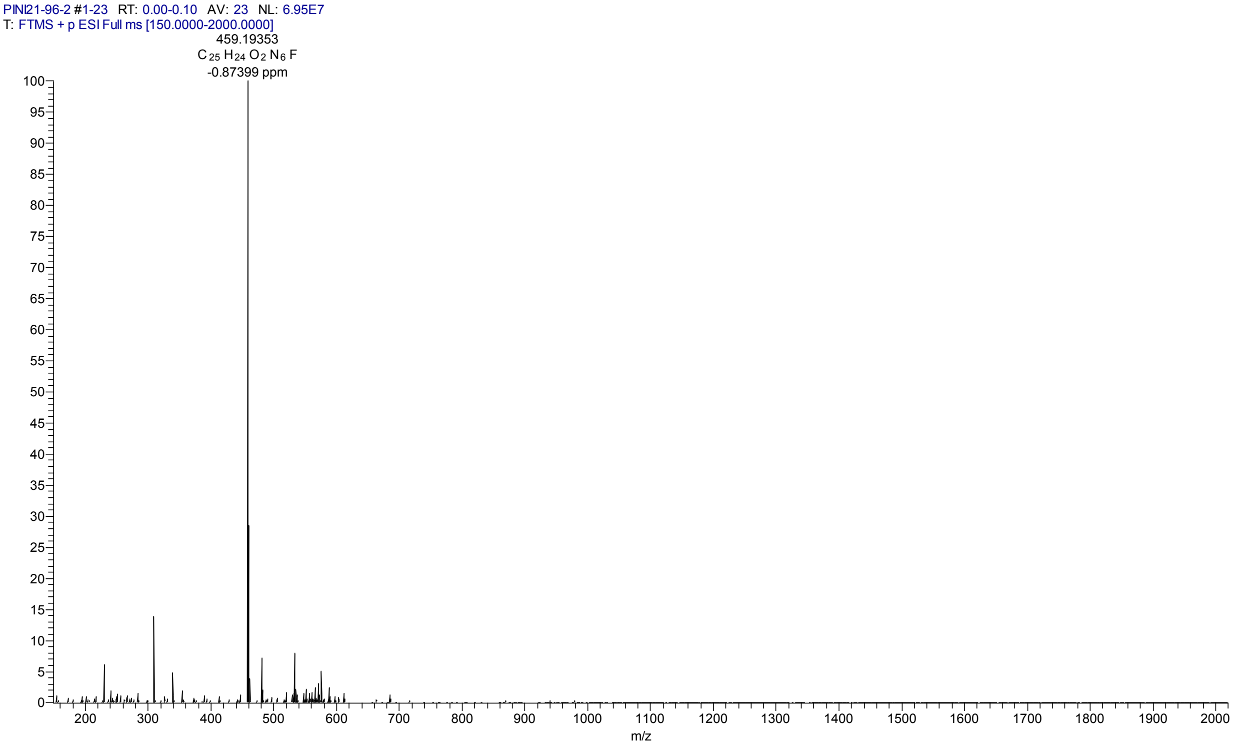

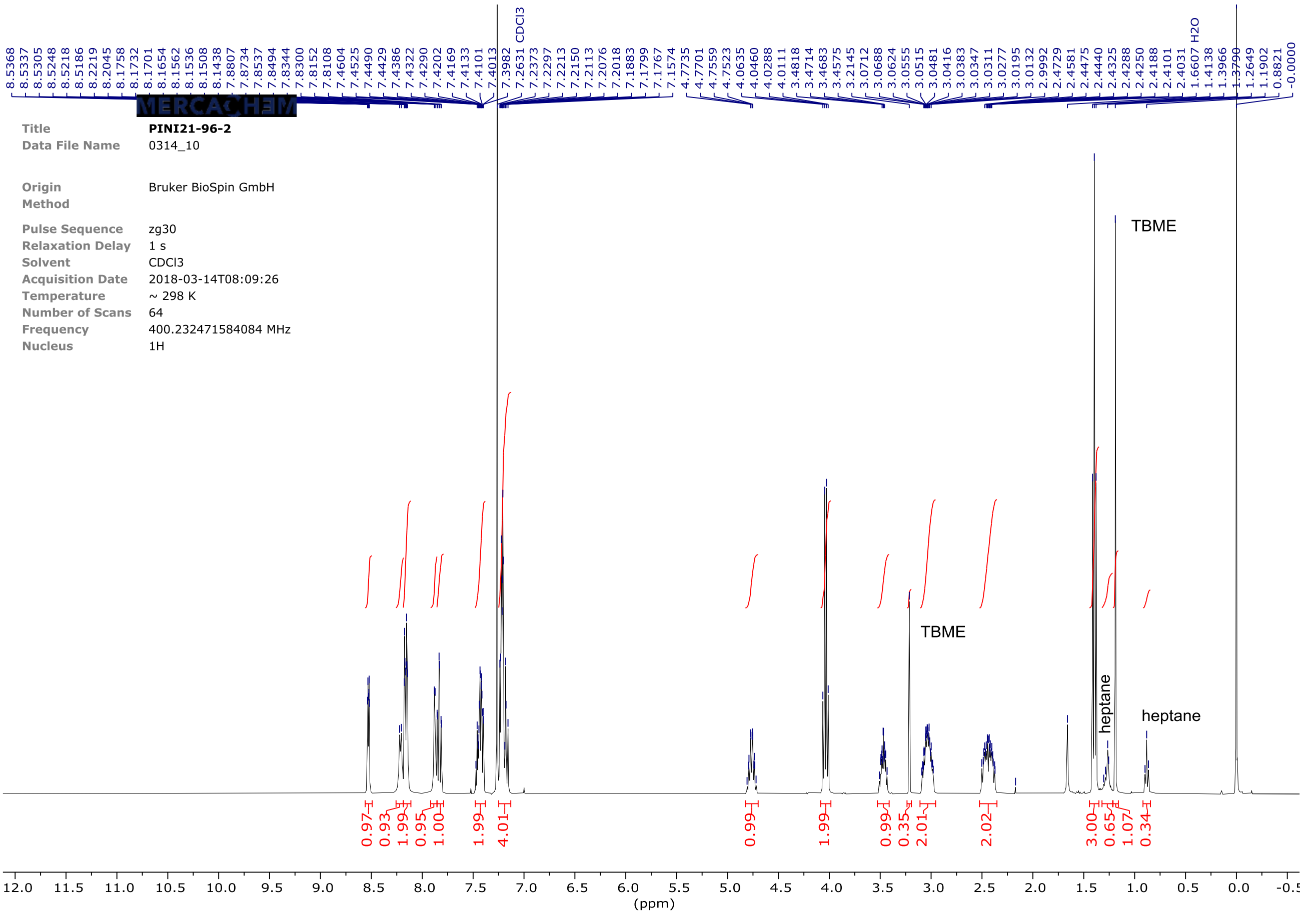

Compound 16

**title**	**PINI21-54-1**
**Method**	AN_ACID.M
**Date acquired**	15-Dec-17, 16:53:28
**FileName**	Analysis\LCMS6_1215_106.D
**Column**	XSelect CSH C18 (50x2.1mm, 3.5μ) valve:3
**Flow**	0.8 ml/min; Column temp: 35°C
**Eluent A**	0.1% Formic acid in acetonitrile
**Eluent B**	0.1% Formic acid in water
**Gradient**	t=0 min 5% A, t=3.5 min 98% A, t=6 min 98% A
**Posttime**	2 min
**Detection**	DAD (210, 220, 220-320nm)
**Detection**	PDA (210-320nm)
**Detection**	MSD (ESI pos/neg) mass range: 100-800
**Detection**	CAD Temp. 40°C, iontrap 20.2 V, chargervoltage 2.64 kV

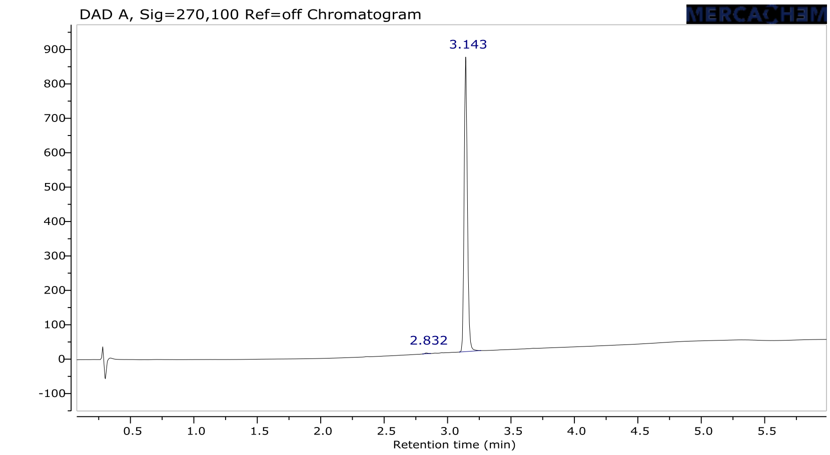

Integrals spectrum Chromatogram DAD A, Sig=270,100 Ref=off

rt (min)	height	area	area (%)
2.83	2.392	0.06006	0.24
3.14	856.5	25.42	99.76

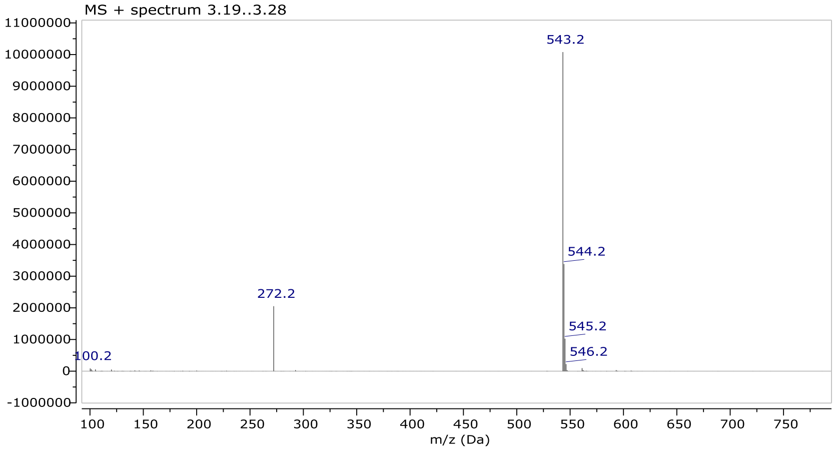

HRMS (m/z): C28H23FN6O3S, [M+H]+ Calc: 543.16091; found: 543.16007, Δppm -0.84

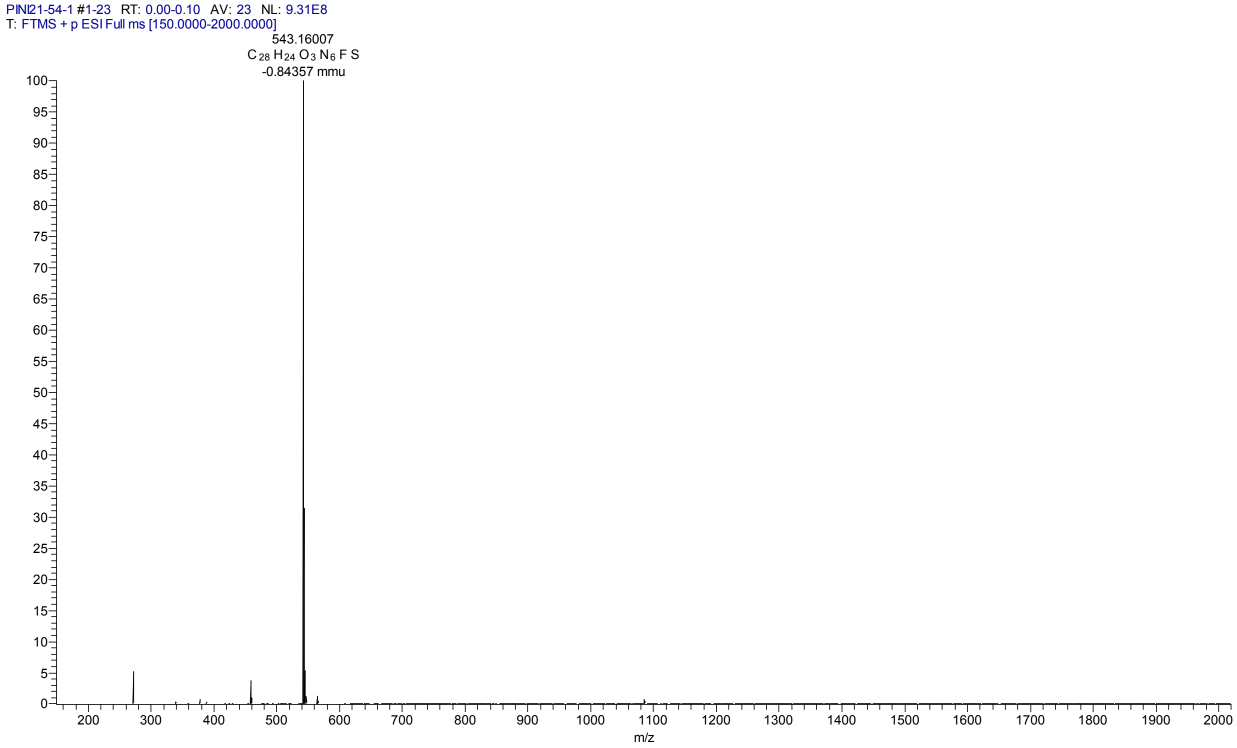

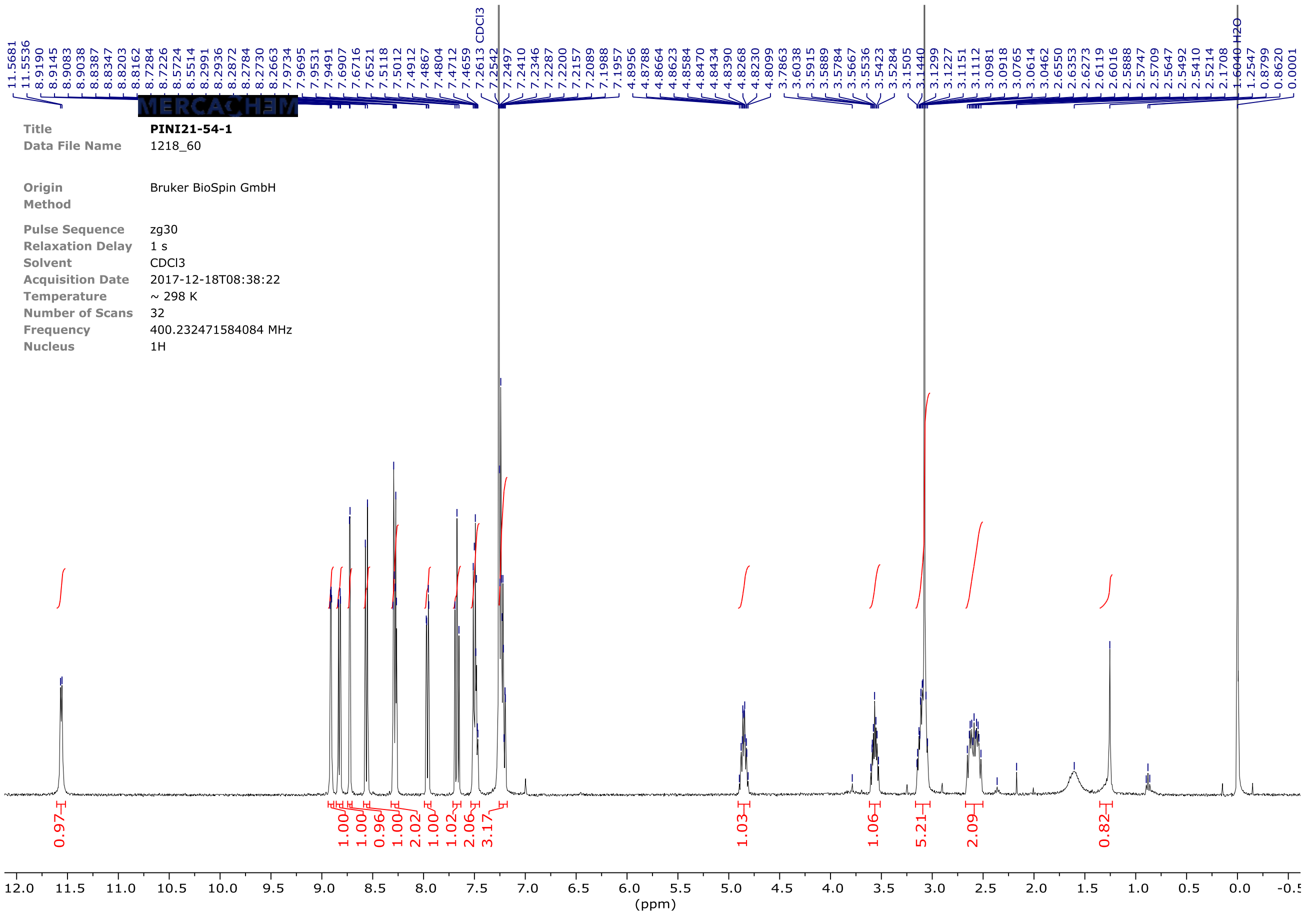

Compound 19

title	**PINI20-26-3**
Method	AN_ACID.M
Date acquired	12-May-17, 19:22:09
FileName	Analysis\LCMS19_0512_089.D
Column	Waters XSelect CSH C18 (50x2.1mm 3.5μ)
Flow	0.8 ml/min; Column Temp: 35°C
Eluent A	0.1% formic acid in acetonitrile
Eluent B	0.1% formic acid in water
Lin. Gradient	t=0 min 5% A, t=3.5 min 98% A, t=6 min 98% A
Posttime	2 min
Detection	DAD (220-320 nm)
Detection	PDA (200-400 nm)
Detection	MSD ESI pos/neg (mass range 100-800)

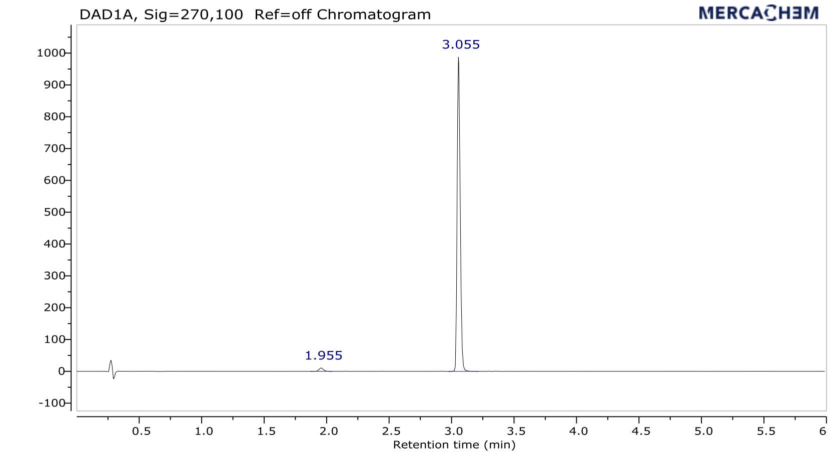

Integrals spectrum Chromatogram DAD1A, Sig=270,100 Ref=off

rt (min)	height	area	area (%)
1.96	10.79	0.4379	1.54
3.06	988.0	27.97	98.46

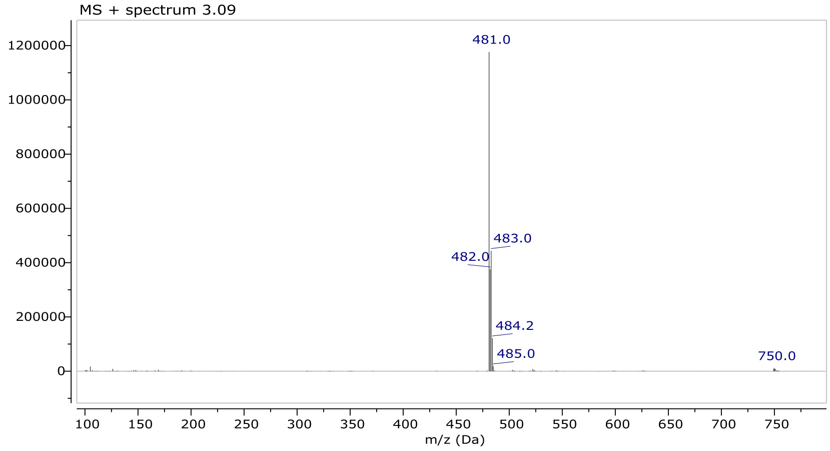

HRMS (m/z): C25H17ClN8O, [M+H]+ Calc: 481.12866; found: 481.12796, Δppm-1.46

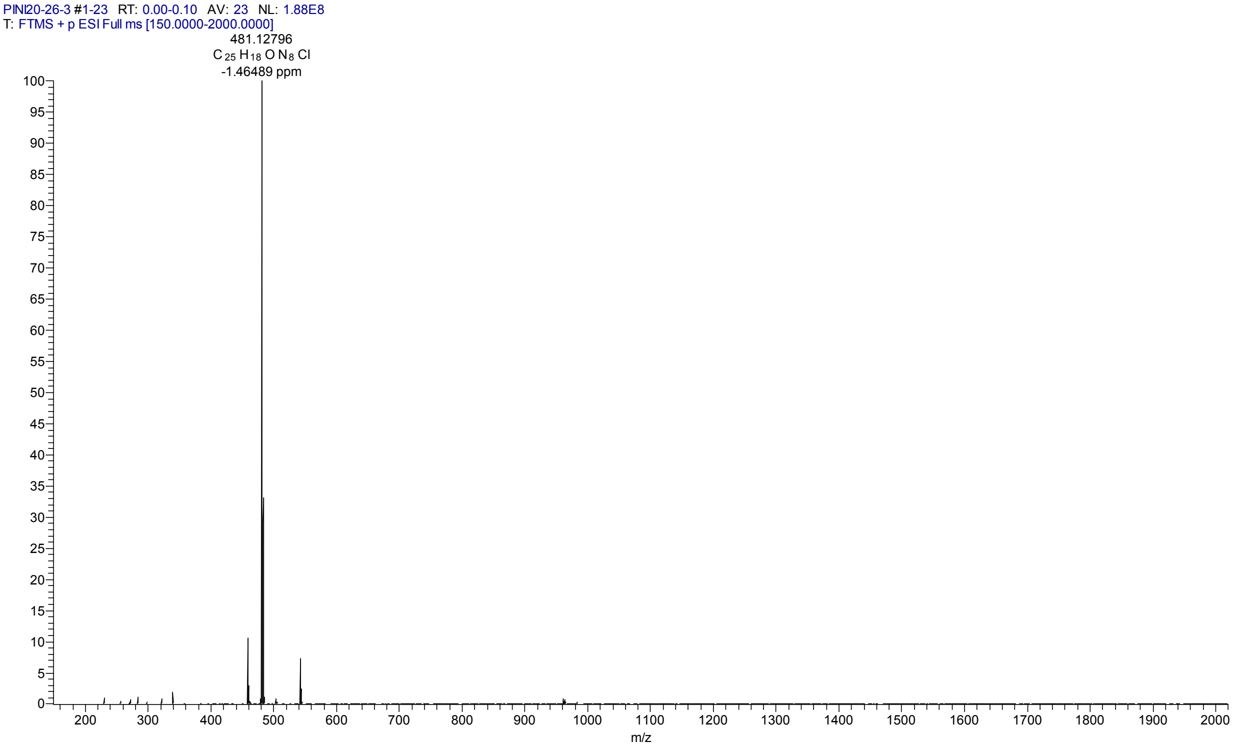

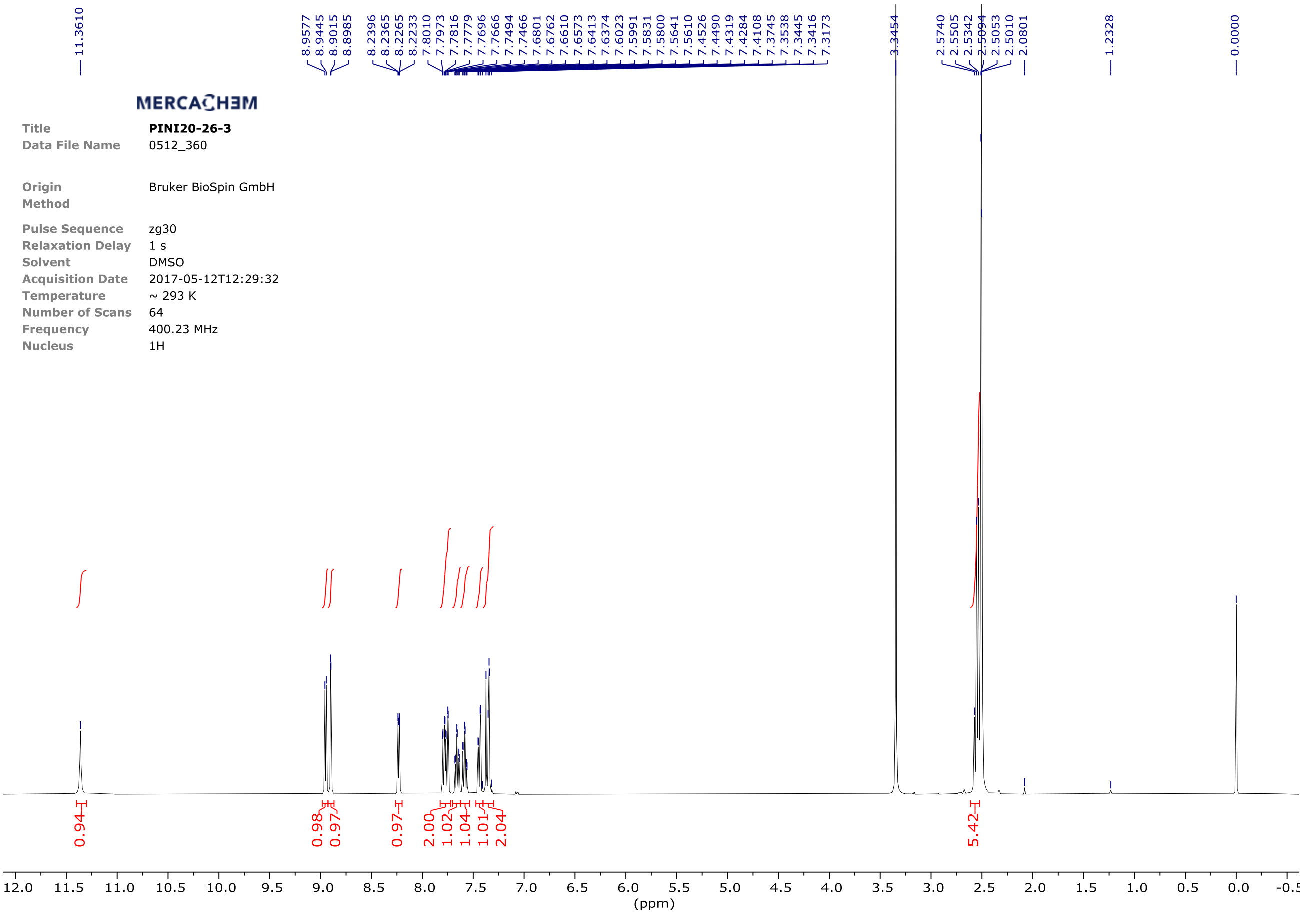

Compound 20

**title**	**PINI20-50-2**
**Method**	AN_BASE.M
**Date acquired**	28-Jun-17, 17:40:35
**FileName**	Analysis\LCMS5_0628_079.D
**Acq. method**	AN_BASE valve: 2
**Column**	Waters XSelect CSH C18 (50x2.1mm, 3.5µ)
**Flow**	0.8 ml/min; Column temp: 25°C
**Eluent A**	95% acetonitrile + 5% 10 mM ammoniumbicarbonate in water
**Eluent B**	10mM ammoniumbicarbonate in water
**Lin. Gradient**	t=0 min 5% A, t=3.5 min 98% A, t=6 min 98% A
**Posttime**	2 min
**Detection**	DAD (220-320 nm)
**Detection**	PDA (200-400 nm)
**Detection**	MSD (ESI pos/neg) mass range: 100-800

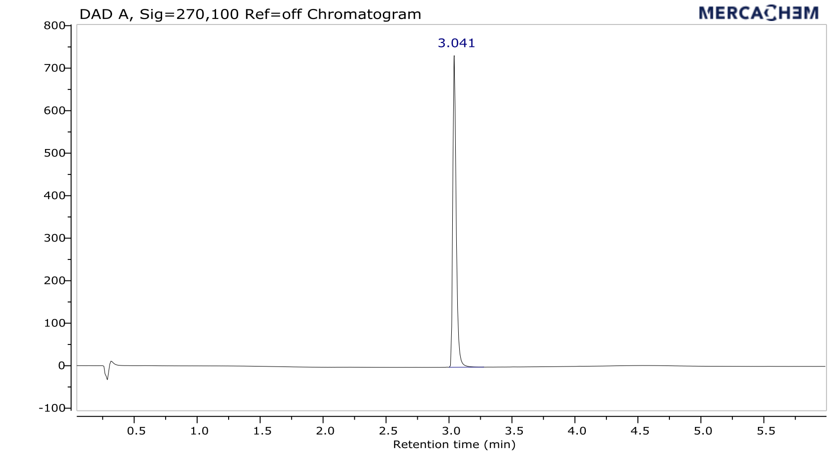

Integrals spectrum **Chromatogram DAD A, Sig=270,100 Ref=off**

rt (min)	height	area	area (%)
3.04	733.3	23.07	100.00

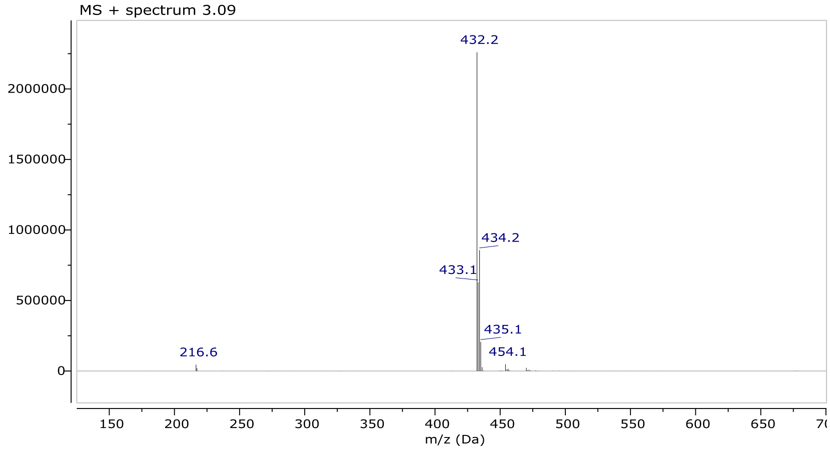

HRMS (m/z): C22H18ClN7O, [M+H]+ Calc: 432.13341; found: 432.13272, Δppm -1.61

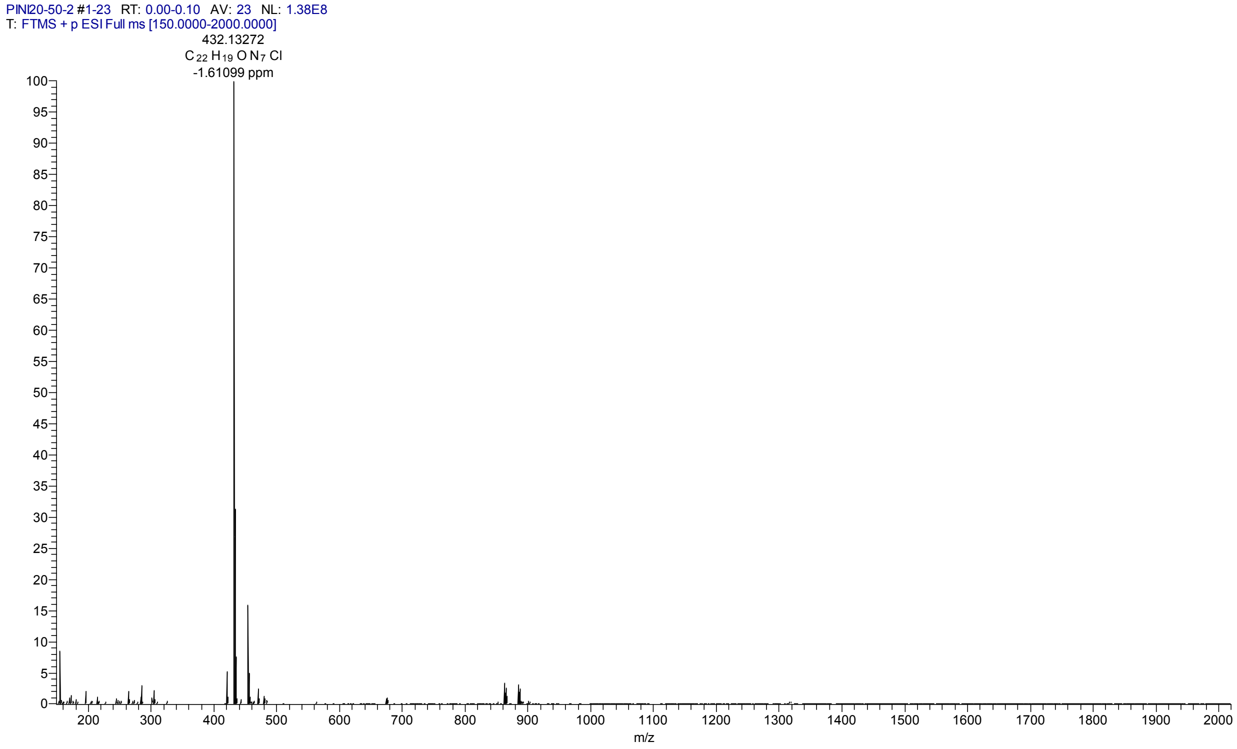

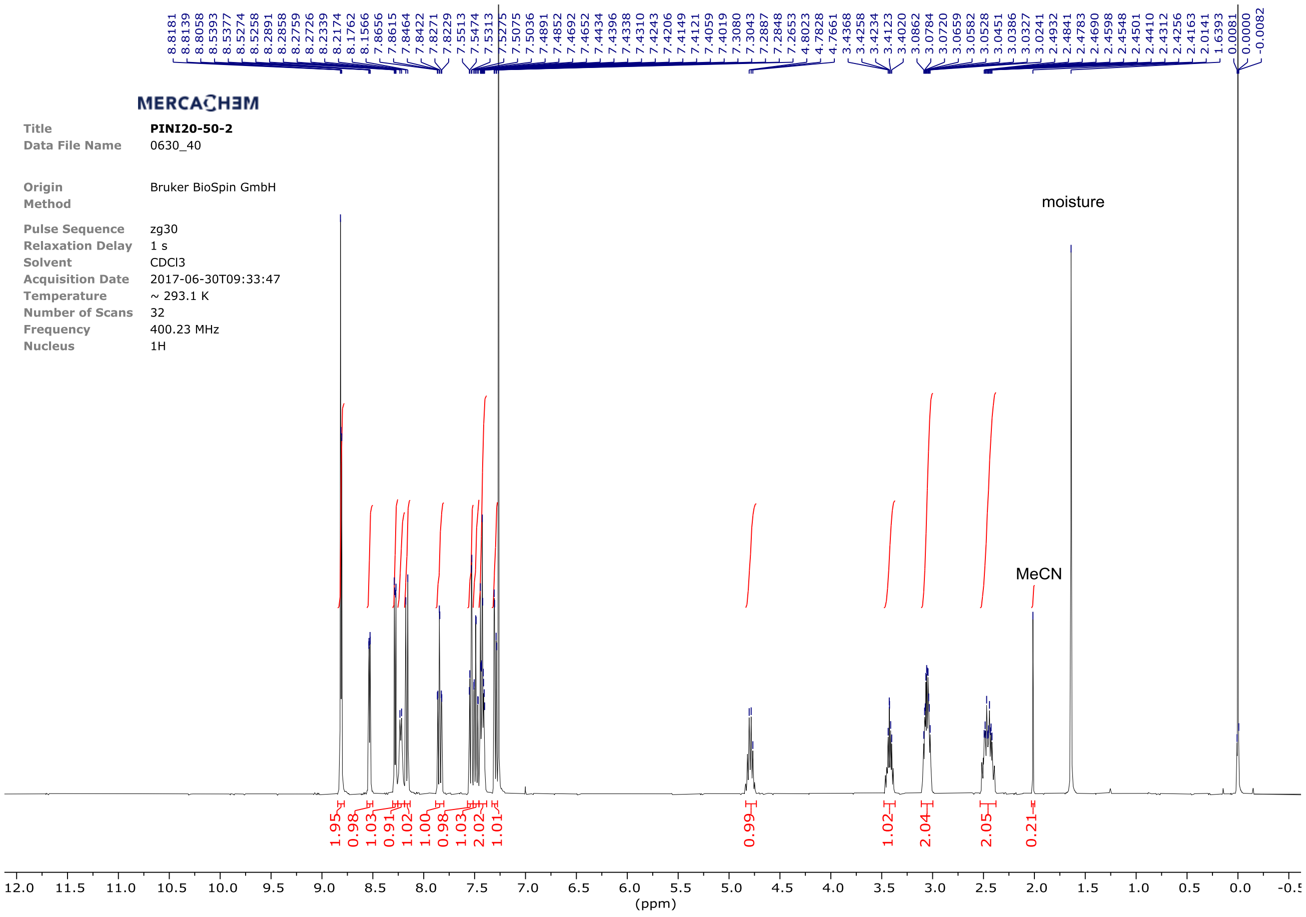

Compound 21

title	**PINI20-17-3**
Method	AN_ACID.M
Date acquired	25-Apr-17, 17:45:46
FileName	Analysis\LCMS19_0425_080.D
Column	Waters XSelect CSH C18 (50x2.1mm 3.5μ)
Flow	0.8 ml/min; Column Temp: 35°C
Eluent A	0.1% formic acid in acetonitrile
Eluent B	0.1% formic acid in water
Lin. Gradient	t=0 min 5% A, t=3.5 min 98% A, t=6 min 98% A
Posttime	2 min
Detection	DAD (220-320 nm)
Detection	PDA (200-400 nm)
Detection	MSD ESI pos/neg (mass range 100-800)

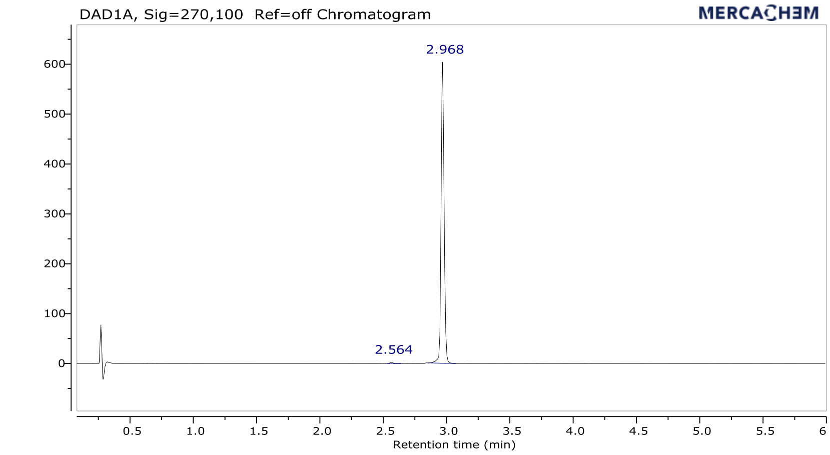

Integrals spectrum Chromatogram DAD1A, Sig=270,100 Ref=off

rt (min)	height	area	area (%)
2.56	2.934	0.07694	0.46
2.97	603.9	16.61	99.54

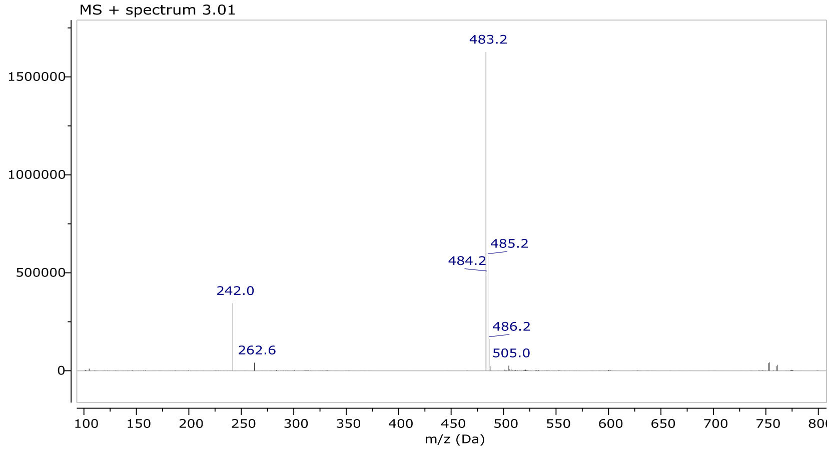

HRMS (m/z): C25H19ClN8O, [M+H]+ Calc: 483.14431; found: 483.14350, Δppm -0.81

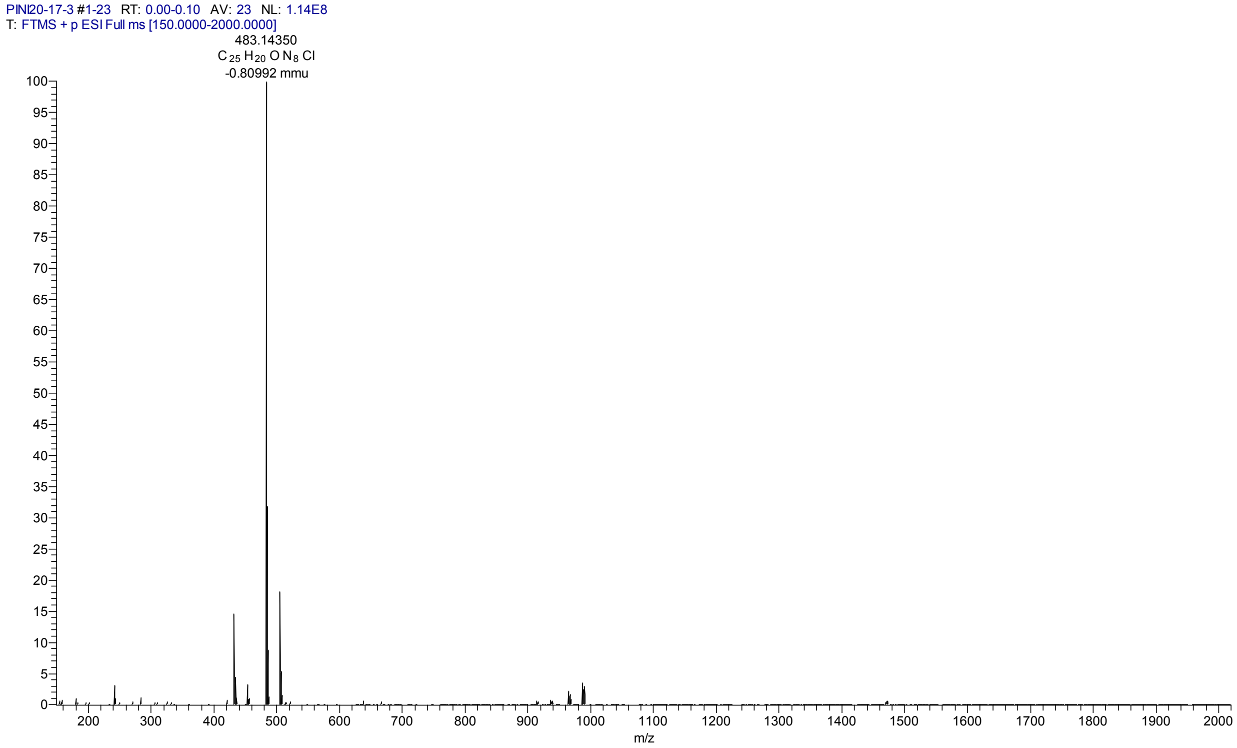

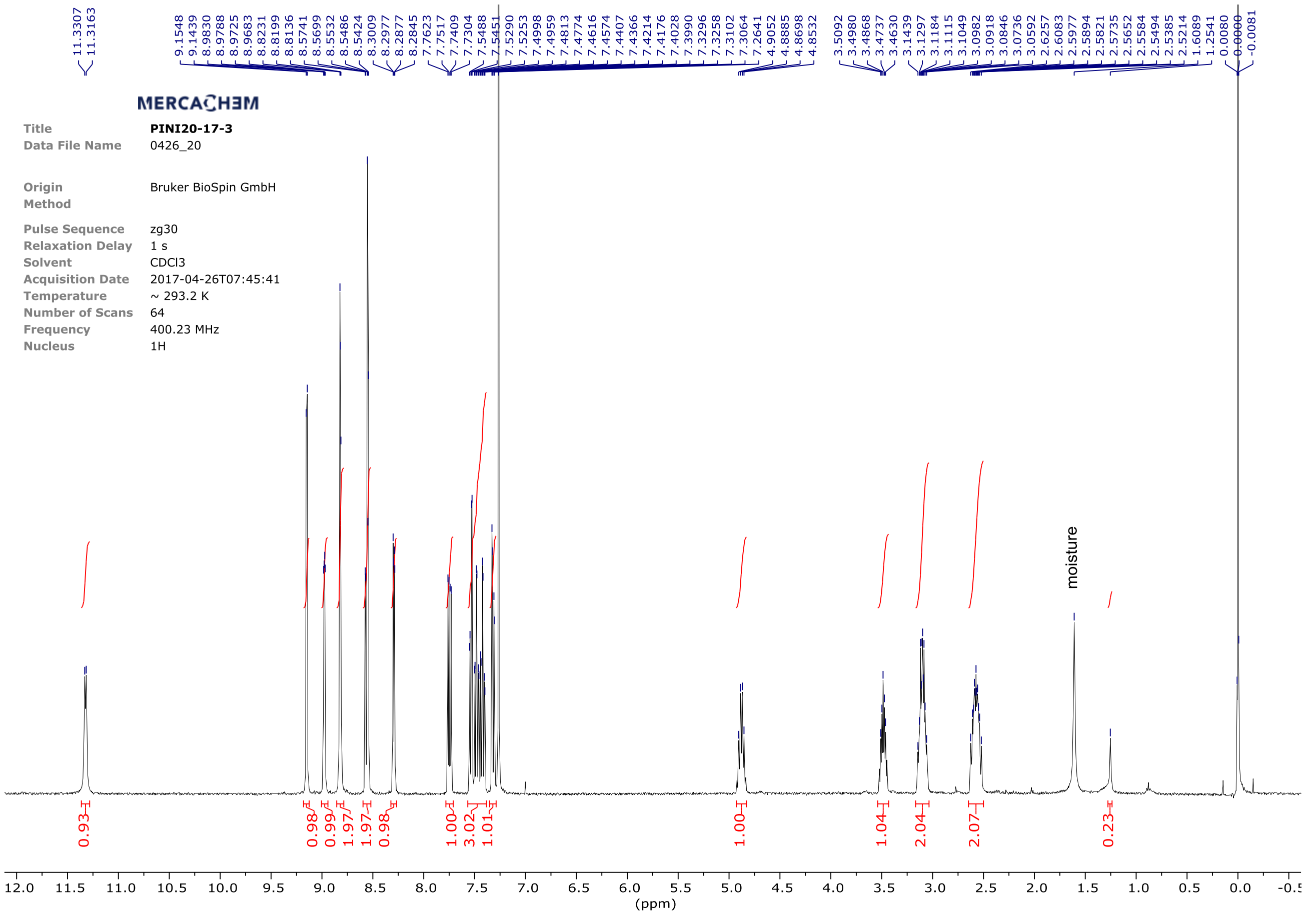

Compound 22

HRMS (m/z): C27H22ClN7O2, [M+H]+ Calc: 512.15963; found: 512.15900, Δppm-1.23

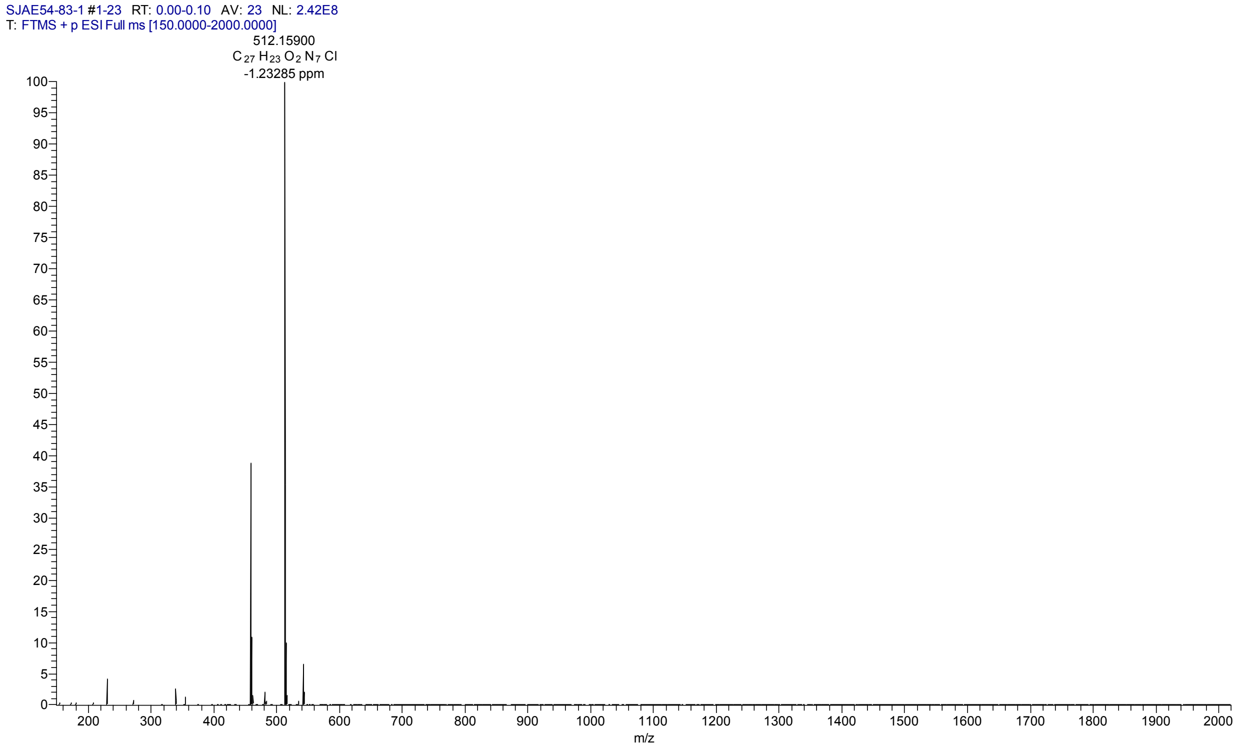

Compound 23

HRMS (m/z): C24H17FN8O, [M+H]+ Calc: 453.15821; found: 453.15769, Δppm -1.16

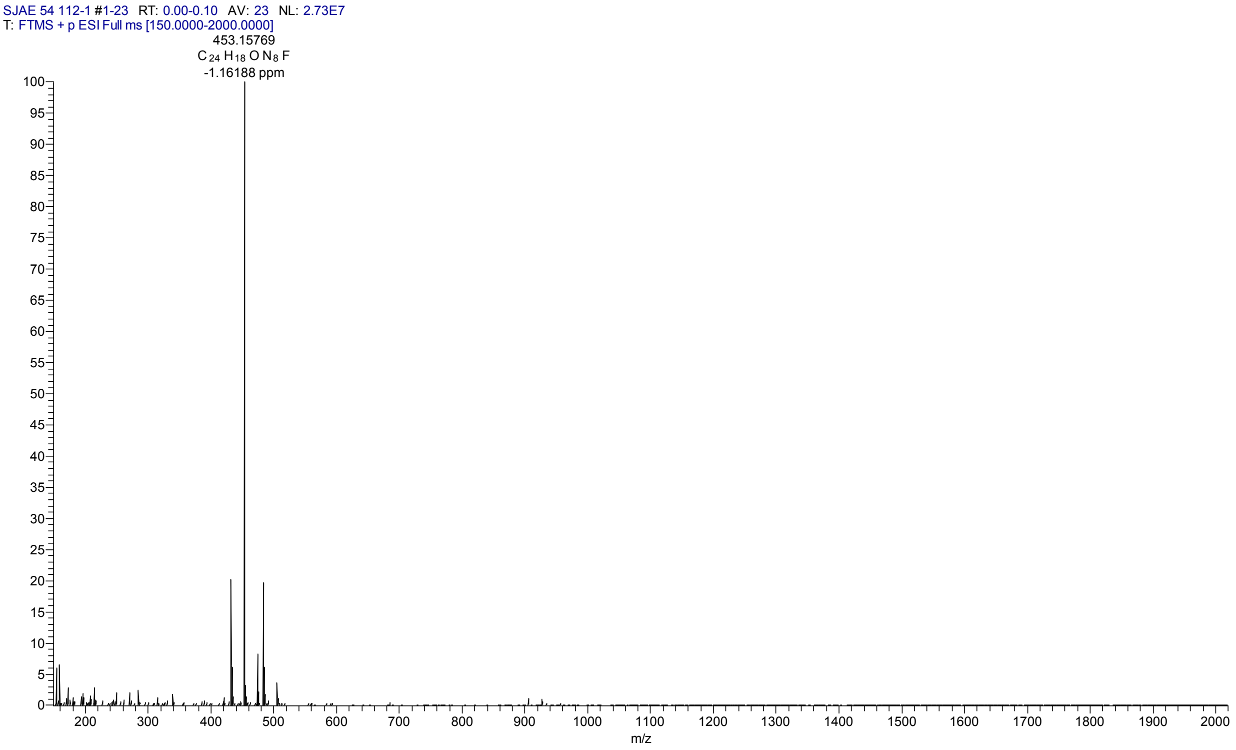

Compound 27

title	**PINI23-30-1**
Method	AN_ACID.M
Date acquired	14-Sep-18, 17:39:04
FileName	Analysis\LCMS6_0914_134.D
Column	XSelect CSH C18 (50x2.1mm, 3.5μ) valve:3
Flow	0.8 ml/min; Column temp: 35°C
Eluent A	0.1% formic acid in acetonitrile
Eluent B	0.1% formic acid in water
Gradient	t=0 min 5% A, t=3.5 min 98% A, t=6 min 98%A
Posttime	2 min
Detection	DAD(210, 220 and 220-320nm)
Detection	PDA(210-320nm)
Detection	MSD (ESI pos/neg) mass range: 100 - 1000
Detection	ELSD gas temp: 40°C, flow 1.5 ml/min, gain 1

Integrals spectrum — Chromatogram DAD A, Sig=270,100 Ref=off

rt (min)	height	area	area (%)
2.65	1.045	0.02621	0.15
3.03	546.0	16.97	99.63
3.26	1.445	0.03695	0.22

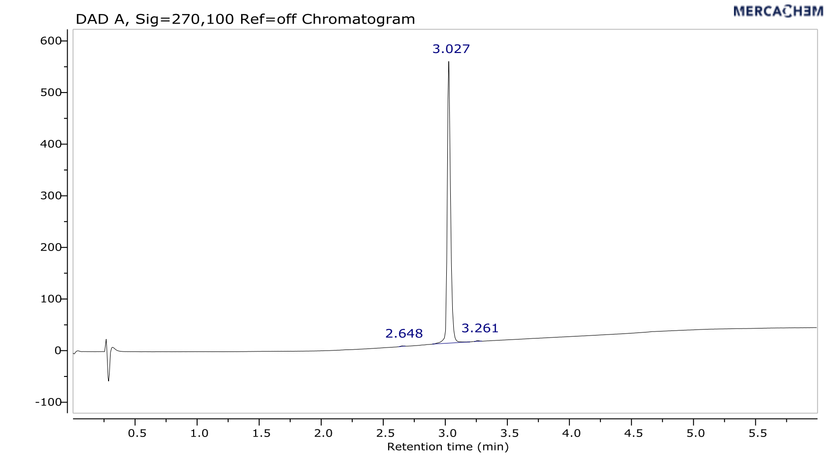

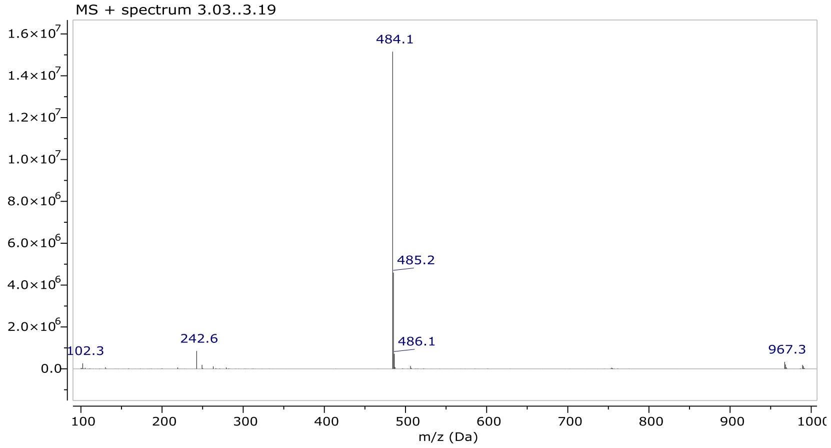

HRMS (m/z): C26H19F2N7O, [M+H]+ Calc: 484.16919; found: 484.16833, Δppm -1.78

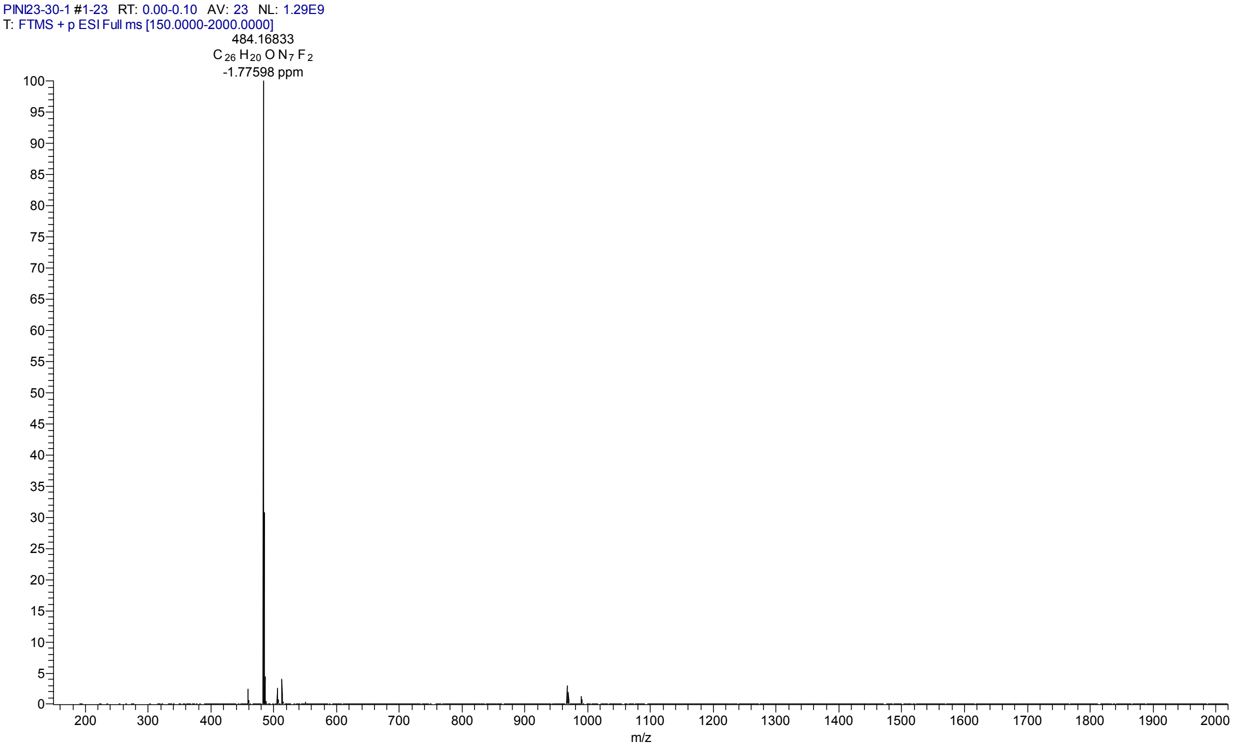

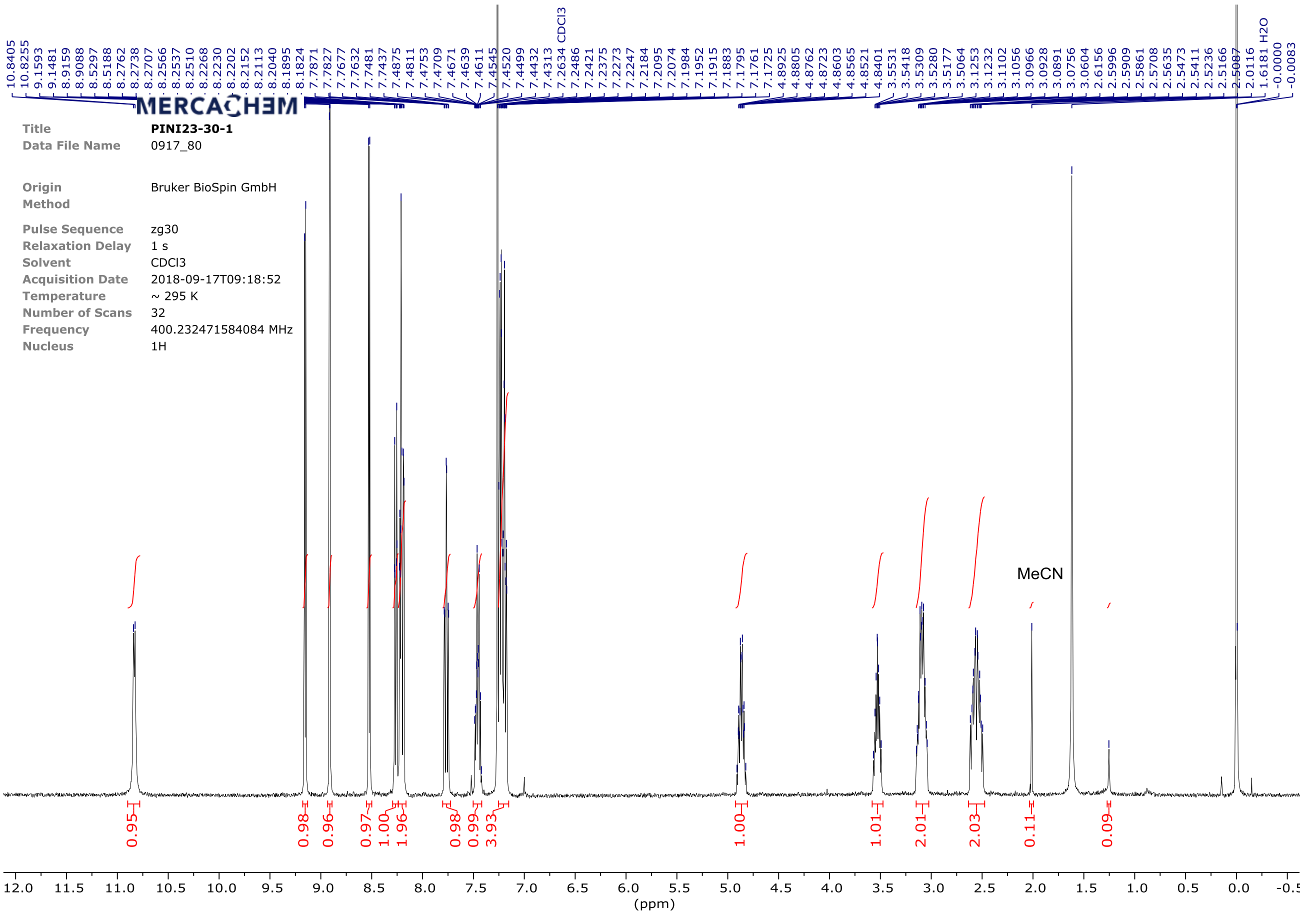

Supplement: Supplementary file 2 — jm0c00208_si_002.pdf [file jm0c00208_si_002.pdf]
